# The Preterm Brain Is Also What It Eats: A Systematic Review of Nutrition and Neuroimaging Outcomes in Preterm Infants

**DOI:** 10.3390/nu18203392

**Published:** 2026-10-16

**Authors:** Maria Ester Canepa, Federico Prefumo, Valentina Biagioli, Pasquale Striano, Andrea Calandrino, Luca Antonio Ramenghi

**Affiliations:** 1 Department of Neuroscience, Rehabilitation, Ophthalmology, Genetics and Mother-Child Health, University of Genoa, 16132 Genoa, Italy; valentina.biagioli@edu.unige.it (V.B.); strianop@gmail.com (P.S.);; 2 Obstetrics and Gynecology Unit, IRCCS Istituto Giannina Gaslini, 16147 Genoa, Italy; federicoprefumo@gaslini.org; 3 Pediatric Neurology and Muscular Diseases Unit, IRCCS Istituto Giannina Gaslini, 16147 Genoa, Italy; 4 Neonatal Intensive Care Unit, IRCCS Istituto Giannina Gaslini, 16147 Genoa, Italy; andrea.calandrino@edu.unige.it

**Keywords:** brain, neonate, nutrition, growth, development, imaging, MRI

## Abstract

**Background**: Nutrition is one of the most modifiable determinants of brain development in preterm infants. Although increasing evidence supports associations between early nutritional exposures and neurodevelopment, studies remain heterogeneous regarding nutritional interventions, imaging biomarkers, and long-term outcomes. This systematic review aimed to summarize contemporary evidence on the relationship between nutrition and brain development in preterm infants using neuroimaging as the primary assessment tool while proposing an integrated translational framework to guide future research. **Methods**: Following a PECO framework, a systematic literature search was conducted in PubMed, Scopus, and CINAHL: population, infants born before 37 completed weeks of gestation; exposure, any enteral or parenteral nutritional exposure, supplementation strategy, fortification approach, or individualized nutritional intervention; comparator, an alternative nutritional strategy, lower or absent exposure, standard care or, when relevant, a term-born reference group; outcomes, MRI- or CUS-based measures of brain growth, maturation, connectivity, or injury. Studies reporting volumetric, morphometric, diffusion, connectivity, spectroscopy, or quantitative ultrasound outcomes were eligible. The methodology was adapted from the PROSPERO-registered review by Ottolini et al. Original studies published between 2020 and 2026 (1 January 2020–4 September 2026) evaluating nutritional exposures or interventions in preterm infants with magnetic resonance imaging (MRI) and/or cranial ultrasound (CUS) outcomes were included. Risk of bias was assessed using the Newcastle–Ottawa Scale (NOS), ROBINS-I and RoB 2 according to study design. Quantitative imaging studies were screened for eligibility for ATLAS assessment. Given the substantial heterogeneity in nutritional exposures, imaging protocols, timing of assessments, and neurodevelopmental outcomes, meta-analysis was not performed. Certainty of evidence was assessed at the level of each causal question and neuroimaging outcome domain using the GRADE framework. Randomized intervention evidence and observational exposure evidence were evaluated according to the design and target contrast contributing to each body of evidence. Judgments considered risk of bias, inconsistency, indirectness, imprecision, publication bias, and, where applicable, factors that could increase certainty. Statistical significance was not used to determine certainty, and studies arising from shared or partially overlapping parent populations were not counted as independent replication. **Results**: We found 33 reports that met the inclusion criteria: 4 evaluated randomized treatment contrasts, 5 non-randomized intervention comparisons, and 24 observational nutritional exposure analyses. Greater exposure to human milk and selected strategies involving early protein and lipid provision, docosahexaenoic acid, omega-3 fatty acids, and individualized nutritional support were associated in several studies with favorable MRI measurements, including larger total or regional brain volumes, more advanced white-matter maturation, and differences in structural or functional connectivity. Some studies also reported associations with later neurodevelopment. However, findings were not uniform, and GRADE certainty across outcome-specific nutrition–neuroimaging questions ranged from very low to low, principally because of risk of bias, imprecision, heterogeneous exposure and imaging definitions, and limited independent replication. Although causal inference cannot be directly drawn, neuroimaging may provide quantitative measures of brain development that can be investigated alongside long-term neurodevelopmental outcomes. We propose the Roadmap for Imaging-Guided Enhancement of Neurodevelopment (RIGEL), an integrative research framework combining imaging, molecular biomarkers, standardized neurodevelopmental assessment, and epidemiological evidence across developmental stages. RIGEL should be regarded as a hypothesis-generating research framework that requires prospective validation before any clinical implementation.

## 1. Introduction

The German philosopher Ludwig Feuerbach is commonly associated with the aphorism “you are what you eat.” In preterm infants, whose brains undergo rapid growth and organization during a period that would normally occur in utero, this concept has particular biological and clinical relevance. Preterm birth remains one of the leading causes of neonatal mortality and long-term neurodevelopmental impairment worldwide. During the last trimester of gestation, the human brain undergoes an extraordinary period of growth characterized by rapid neurogenesis, dendritic arborization, synaptogenesis, gliogenesis and progressive myelination [1,2,3]. Infants born very preterm are suddenly deprived of the intrauterine environment during this critical developmental window, exposing the immature brain to nutritional deficits, inflammation, oxidative stress and intensive care interventions that may permanently influence cerebral maturation [4,5,6,7,8,9]. Consequently, optimizing early nutritional support has become a major component of neuroprotective strategies in neonatal intensive care units. Neonatal nutrition, antenatal Doppler abnormalities, microbiome composition, and intestinal morbidities such as necrotizing enterocolitis have each been associated with neurodevelopmental and behavioral outcomes [10,11,12,13,14,15,16,17,18,19,20,21]. These observations support continued attention to nutritional strategies beyond the neonatal intensive care unit, including the post-discharge period [22,23]. Particularly, Massirio et al. [22] underscore that nutrition and extrauterine growth delay are important dimensions in neonatal brain injury. Emerging lipidomic data further suggest that abnormal fetal Doppler patterns may be accompanied by alterations in cord-blood lipid composition, including lower concentrations of DHA-containing phospholipids and higher triglycerides [24]. Such findings raise the possibility that nutritional and metabolic vulnerability begins before birth and continues throughout neonatal adaptation. Clinical nutritional management must nevertheless balance potential neurodevelopmental benefits against gastrointestinal tolerance and complications, including spontaneous intestinal perforation and necrotizing enterocolitis [25]. Human milk may provide benefits extending beyond nutrient delivery, including analgesic effects during neonatal procedures [26]. Multiple clinical trials, consensus documents, and professional-society statements have attempted to define standards of nutritional care [27,28,29,30,31,32,33,34,35,36], but recommendations remain fragmented across gestational age groups and populations such as very-low-birth-weight and extremely preterm infants.

Neuroimaging offers an important means of examining how nutritional exposures may relate to brain growth, tissue maturation, connectivity, and injury. Advances in neonatal MRI, diffusion tensor imaging, magnetic resonance spectroscopy and quantitative cranial ultrasonography now allow investigators to identify structural and functional brain alterations long before neurodevelopmental deficits become clinically evident. Ottolini and Keller systematically reviewed the effects of nutrition on the neonatal brain using MRI [37]. MRI can characterize volumetric and morphometric differences associated with maternal and neonatal factors, including anemia, vitamin B12 deficiency, omega-3 intake, alcohol exposure, intraventricular hemorrhage, and other neonatal morbidities [38,39,40,41,42,43,44,45,46,47,48].

Although observational studies and randomized trials have increasingly investigated the relationship between neonatal nutrition and brain development, the evidence remains fragmented across nutritional exposures and interventions, imaging modalities, developmental time points, and outcome measures. Consequently, it remains difficult to determine which nutritional strategies are consistently associated with neuroimaging measures of preterm brain development [49,50,51].

We, therefore, aimed to update the evidence published from 2020 through to 2026 on nutritional exposures or interventions and brain outcomes in preterm infants assessed by MRI and/or CUS. We also sought to identify methodological gaps and to propose a structured translational framework—RIGEL—for integrating imaging with molecular, developmental, and epidemiological outcomes.

The framework is conceptually aligned with broader approaches to effective translation in neonatal neuroprotection [52,53,54,55] but is specifically tailored to nutrition-related research.

## 2. Methodology

### 2.1. Review Question and Eligibility Criteria

The review question was structured according to a Population, Exposure, Comparator, and Outcome (PECO) framework: population, infants born before 37 completed weeks of gestation; exposure, any enteral or parenteral nutritional exposure, supplementation strategy, fortification approach, or individualized nutritional intervention; comparator, an alternative nutritional strategy, lower or absent exposure, standard care or, when relevant, a term-born reference group; outcomes, MRI- or CUS-based measures of brain growth, maturation, connectivity, or injury. Studies reporting volumetric, morphometric, diffusion, connectivity, spectroscopy, or quantitative ultrasound outcomes were eligible. The full search strings for PubMed, CINAHL and Scopus are available in Supplementary Material. They were adapted with PolyGlot software 0.3.7 [56] accordingly. Zotero 10.0.4 [57], Elicit [58] and BOOTHIE 1.5 were chosen for reference management, data extraction, and preliminary risk-of-bias assessment, respectively. Data categories were aligned with Ottolini et al.’s review (population; study type; nutritional intervention; MRI outcome and key results considering, specifically, TEA, follow-up and neurodevelopmental outcomes), adding a further category (study country) to geographically contextualize the dynamics between neonatal nutrition and brain MRI morphometrics.

### 2.2. Protocol and Reporting

The present review was reported in accordance with PRISMA 2020 [59]. This review updated and expanded the methodology of the PROSPERO-registered review by Ottolini and Keller (CRD42019119577) [37]. Because the present work was not itself prospectively registered, this should be acknowledged as a methodological limitation.

### 2.3. Information Sources and Search Strategy

Search string. PubMed, Scopus, and CINAHL were searched for original studies published from 1 January 2020 to 4 September 2026. The PubMed strategy was adapted from Ottolini et al. and expanded to include terms related to CUS. Search strings were translated for Scopus and CINAHL using Polyglot Search Translator [56,60] and manually checked before execution (complete search string in Supplementary Material).

### 2.4. Study Selection

After duplicate removal, titles and abstracts were screened by the first author. Potentially eligible reports underwent full-text assessment. Uncertain eligibility decisions were discussed with the last author when necessary. Reasons for full-text exclusion were recorded. Reviews, meta-analyses, case reports, letters, animal studies, fetal-only or maternal-only studies, studies restricted to term-born populations, and reports without an eligible nutritional exposure or neuroimaging outcome were excluded.

Data were extracted into a prespecified form, including study country, design, sample size, gestational age, nutritional exposure or intervention, comparator, imaging modality, timing of imaging, imaging outcome, neurodevelopmental assessment, duration of follow-up, covariates, and principal findings. Automated extraction tools, when used, supported data collection but never replaced manual verification against each source article. First author executed the data extraction. Accordingly, data extraction was not duplicated.

### 2.5. Risk of Bias and Imaging Method Assessment

Risk of bias was assessed according to the target contrast evaluated in each report. Randomized treatment contrasts were assessed using RoB 2, non-randomized intervention comparisons using ROBINS-I, and observational nutritional exposure analyses using the Newcastle–Ottawa Scale (NOS). Risk-of-bias assessment was assisted by AI-supported tools, including BOOTHIE 1.5 and ChatGPT 5.6, to facilitate application of RoB 2, ROBINS-I, and NOS criteria. The first author reviewed the resulting judgments against the source reports where feasible. No independent duplicate human assessment was performed; this is acknowledged as a limitation. Studies involving artificial intelligence applied to imaging, radiomics, or comparable quantitative imaging pipelines were considered for ATLAS assessment. One included study met the predefined criteria for a full ATLAS evaluation [51].

### 2.6. Synthesis

Reports were grouped primarily by nutritional exposure and causal question and subsequently by study design, neuroimaging domain, and developmental time point. A random-effects meta-analysis was planned only if at least three studies were sufficiently comparable in population, exposure definition, imaging outcome, and assessment timing. Because these conditions were not met, results were synthesized narratively. Heterogeneity was characterized clinically and methodologically rather than inferred solely from study design.

### 2.7. Certainty Evidence Assessment (GRADE)

Certainty of evidence was assessed using the Grading of Recommendations Assessment, Development and Evaluation (GRADE) approach at the level of each nutrition–neuroimaging question and outcome domain. Randomized and non-randomized evidence was evaluated according to the design and target contrast contributing to each body of evidence. For non-randomized intervention studies assessed with ROBINS-I, certainty was initialized according to the GRADE approach for ROBINS-I-based assessments. Certainty judgments considered risk of bias, inconsistency, indirectness, imprecision, and publication bias, together with factors that may increase certainty where applicable. Reports arising from the same or partially overlapping parent populations were not considered independent replications.

Because quantitative pooling was not appropriate, certainty judgments were based on the study-level effect estimates and confidence intervals where available, sample size and information size, consistency in the direction and magnitude of effects across independent populations, and clinical and methodological directness to the review question. Statistical significance was not used as a criterion for rating certainty. When only one independent study contributed to an outcome-specific body of evidence, inconsistency was considered not assessable rather than automatically downgraded. Overall certainty was categorized as high, moderate, low, or very low. The certainty assessment was added during revision and was not prespecified in the original review protocol.

## 3. Results

Due to study heterogeneity, included papers were analyzed with a Synthesis Without Meta-Analysis approach (SWiM) [61] after providing general characteristics emerging from the present systematic review pipeline.

### 3.1. Study Selection and Characteristics

**Figure 1:**
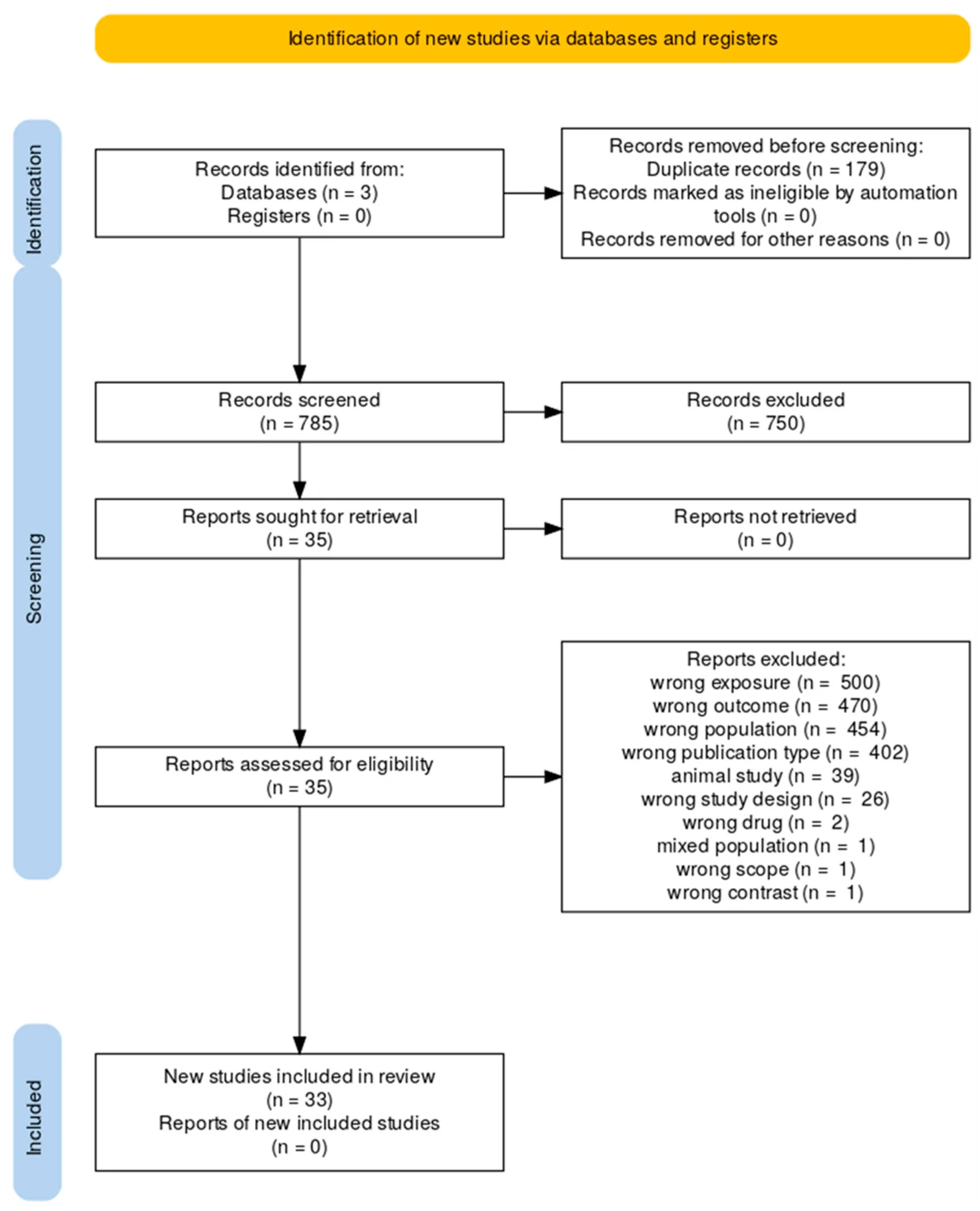
PRISMA 2020 flow diagram of study selection. Arrows connect every screening phase on Rayyan (identification, screening and included) leading to the included articles set (included). The reasons of excluded studies are reported in the dedicated square (**right**) connected with “reports assessed for eligibility” (**left**).

**Figure 2:**
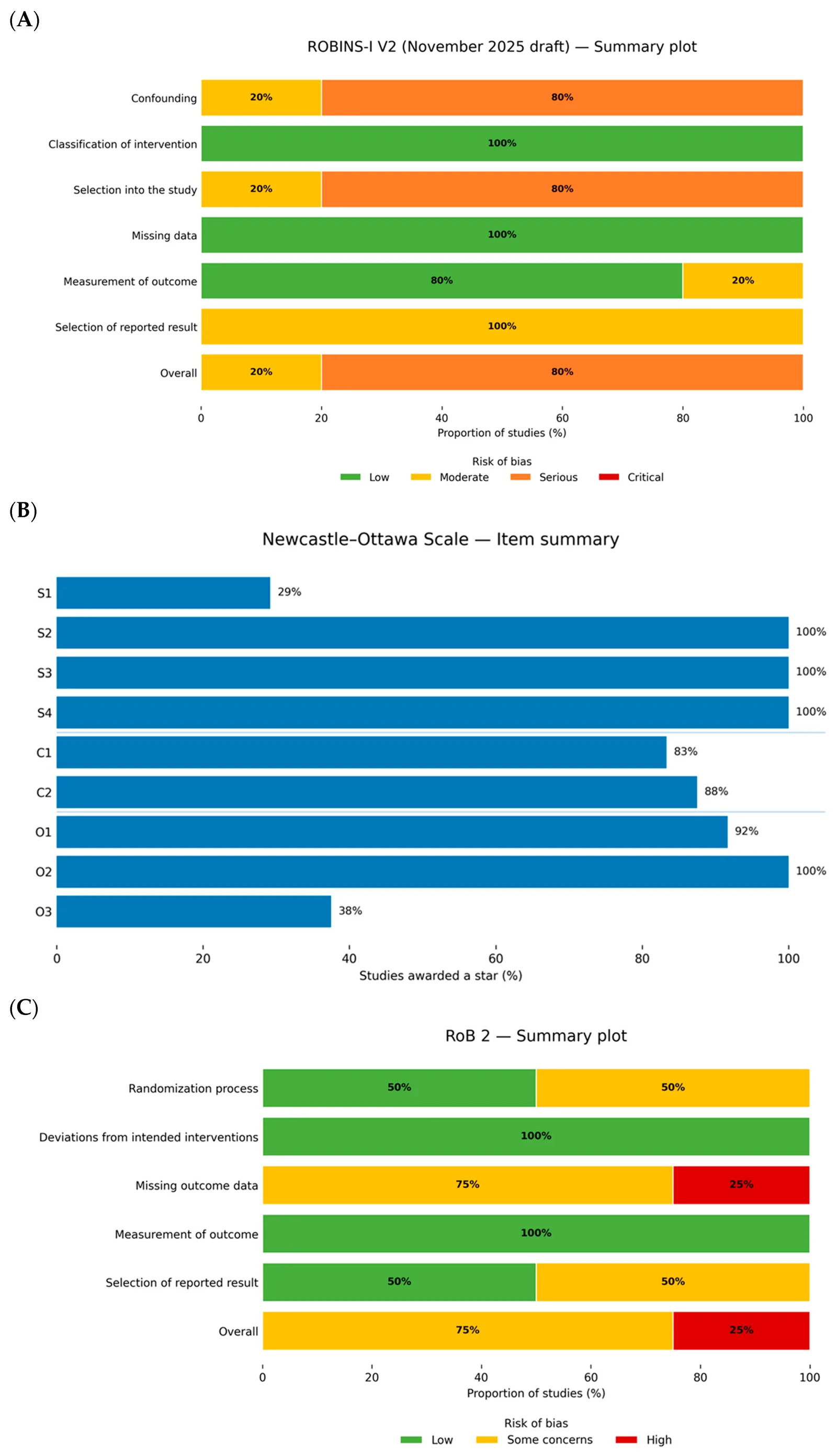
Risk-of-bias summaries: (**A**) ROBINS-I; (**B**) NOS; (**C**) RoB 2. Plots were generated using BOOTHIE from the final risk-of-bias judgments.

Thirty-five full-text reports were assessed for eligibility [51,62,63,64,65,66,67,68,69,70,71,72,73,74,75,76,77,78,79,80,81,82,83,84,85,86,87,88,89,90,91,92,93,94,95]. Two were excluded following full-text reassessment, one because it represented a neurodevelopmental follow-up without an eligible standalone neuroimaging outcome and one because it did not provide an eligible nutritional exposure/intervention contrast. Thirty-three reports, therefore, met the predefined eligibility criteria and were included in the systematic review [51,62,63,64,65,66,67,68,69,70,72,73,74,75,76,77,78,79,80,81,82,83,84,85,86,87,88,89,91,92,93,94,95]. Two studies were, respectively, retrieved with main citation path and snowballing [51,78]. The PRISMA flow diagram is shown in Figure 1 [96]. The 33 eligible reports did not represent 33 independent populations. Parent-study linkage in Table 1 approximately identified 24 study/cohort families among the 33 included reports; therefore, reports arising from the same or partially overlapping parent populations were not interpreted as independent replications apart from multiple multi-report families, including DoMINO, PIANO, the Utrecht nutritional-regimen cohort, the Brigham maternal-milk cohort, and a series of partially overlapping reports from the Children’s National prospective prematurity research program. Summary risk-of-bias assessments are presented in Figure 2 and in the Supplementary Material.

**Figure 3:**
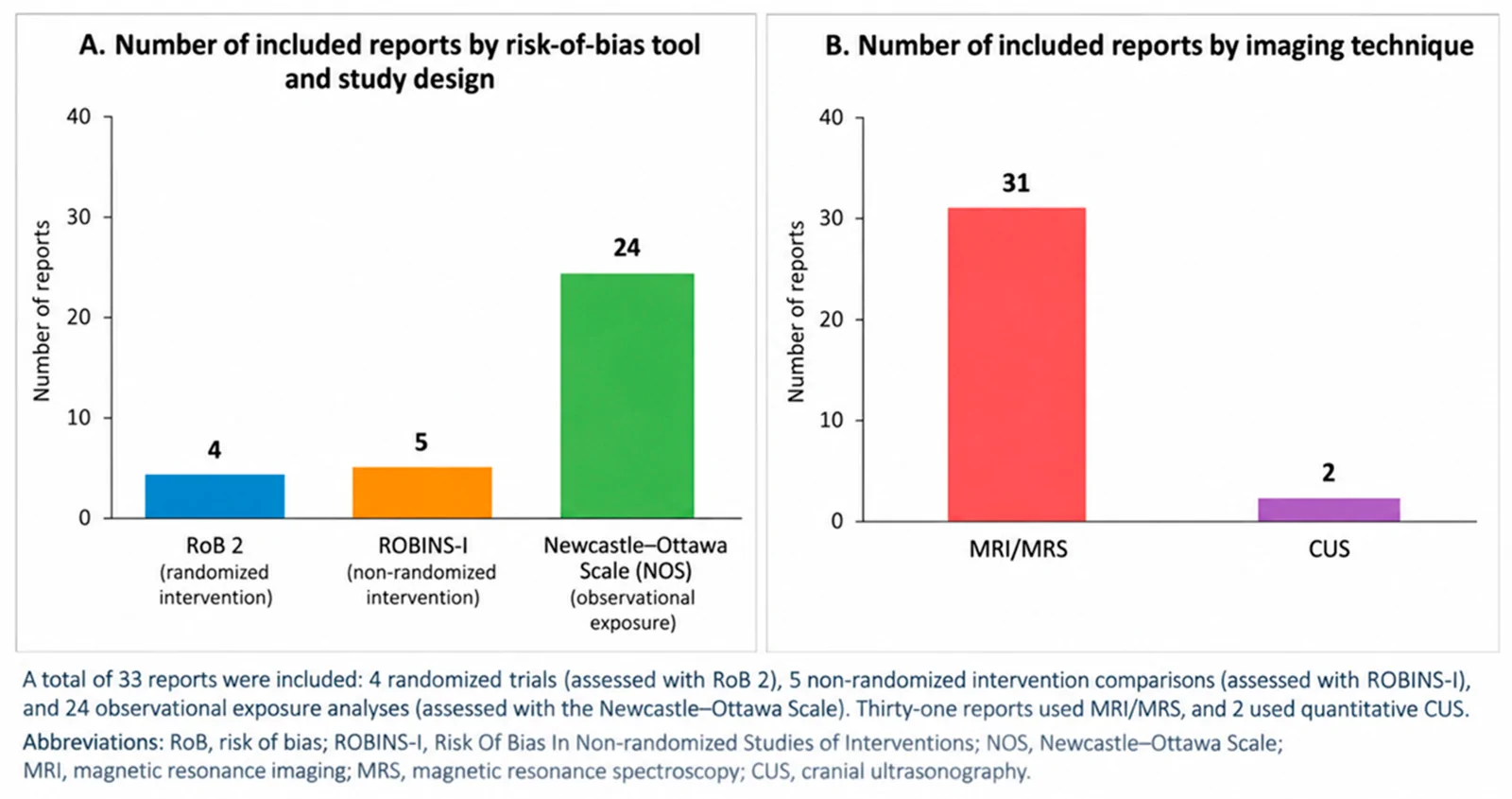
Distribution of included studies according to imaging modality and study design.

**Figure 4:**
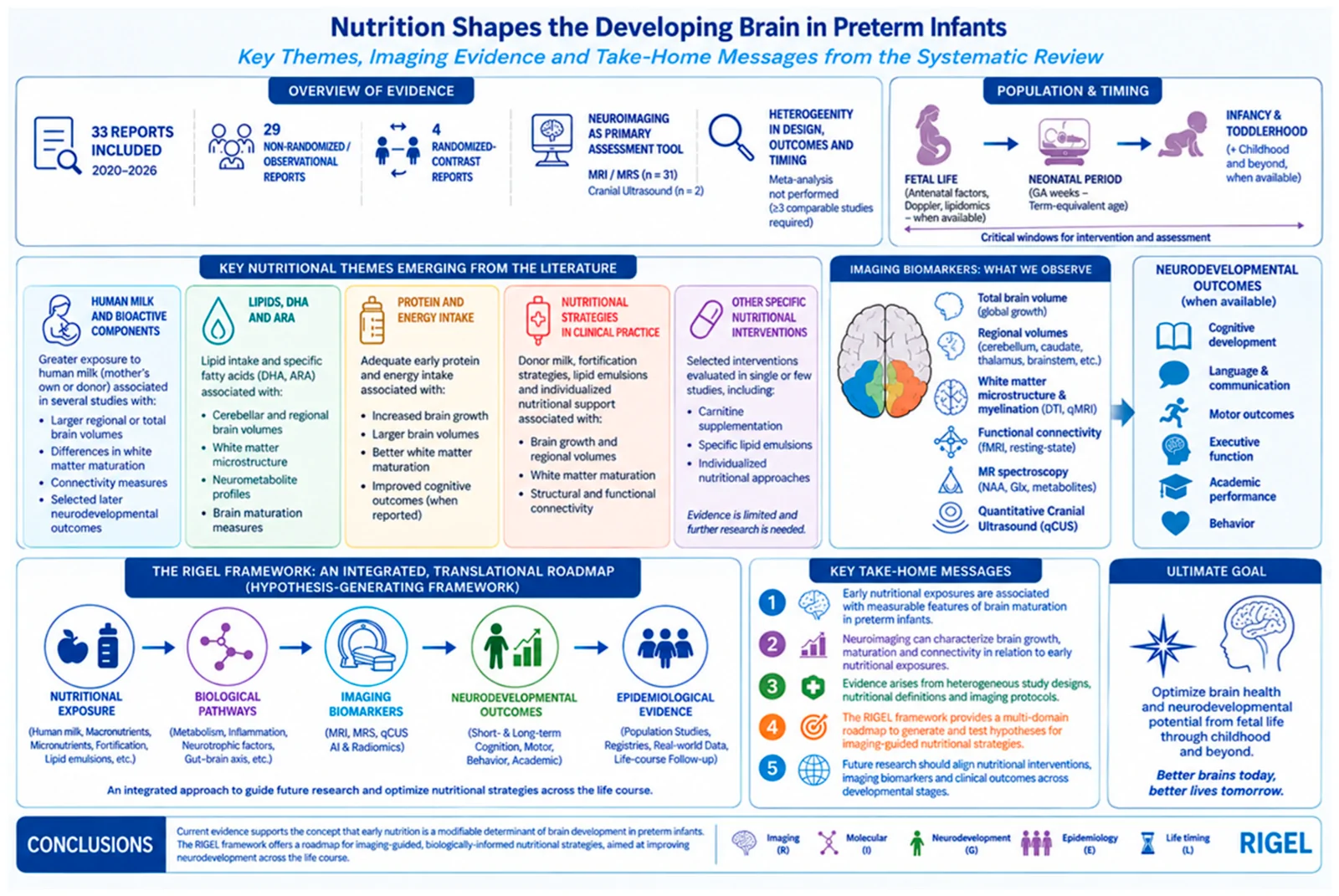
Summary of the principal nutritional exposures, neuroimaging domains, and reported neurodevelopmental associations across the 33 included reports.

Included studies were composed of 4 randomized contrasts, 5 non-randomized intervention comparisons and 24 observational exposure analyses. Further, 31 reports adopted MRI [62,63,64,65,66,68,69,70,71,72,73,74,75,76,77,78,79,80,81,82,83,84,85,86,87,88,89,91,92,93,94,95], while 2 reports chose cranial ultrasounds [51,67] as illustrated in Figure 3. Figure 4 shows the most salient topics emerging from this systematic review. Detailed study characteristics and extracted data are provided in the Supplementary Material’s “Extraction Data Document” with (a) population: sample size; gestational age, birth weight; neonatal comorbidities; (b) nutritional intervention (type, timing and number of sessions, differentiating additionally mother’s own milk, donor milk, formula and fortification strategy); (c) anthropometric features and growth trajectories (head circumference and growth trajectories, interpreting growth-adjusted models as direct rather than total effects; (d) MRI results (volumetrics; morphometrics; maturation); (e) key neurodevelopmental outcomes at follow-up and at TEA; (f) study country was also included to facilitate interpretation of geographical and health-system variations. One study was further analyzed with ATLAS Notebook [51]. Study country was included to facilitate the interpretation of geographical and health-system variation.

### 3.2. Risk of Bias

Four reports evaluating randomized treatment contrasts were assessed using RoB 2 [69,72,77,78]. Among the 29 reports evaluating non-randomized contrasts, 5 non-randomized intervention comparisons were assessed using ROBINS-I [62,68,84,85,93], whereas 24 observational exposure analyses were assessed using the Newcastle–Ottawa Scale (NOS) [51,63,64,65,66,67,70,71,73,74,75,76,80,81,82,83,87,88,89,91,92,94,95] for observational exposure studies according to the examined contrast. The principal concerns in observational studies were residual confounding, variation in exposure definition, and incomplete adjustment for illness severity, socioeconomic factors, growth, and co-interventions. In randomized studies, concerns most commonly related to missing outcome data, secondary imaging analyses, or absence of a prespecified imaging endpoint.

### 3.3. SWiM Framework

Results were synthesized according to the Synthesis Without Meta-Analysis (SWiM) reporting guidelines [61]. Reports were grouped primarily by nutritional exposure or intervention and subsequently by study design, neuroimaging domain, and developmental time point. Where multiple reports originated from the same parent study or potentially overlapping cohort, they were treated as multiple reports from a shared population rather than as independent replications. Effect direction was interpreted together with effect magnitude and uncertainty where these were reported, rather than according to statistical significance alone. Quantitative pooling was considered within each exposure group but was undertaken only when at least three reports were sufficiently comparable in population, exposure definition, imaging endpoint, and assessment timing. No exposure group fulfilled these criteria.

#### 3.3.1. Human Milk and Milk-Derived Nutritional Exposure

Human milk was the most frequently investigated nutritional exposure, although its operational definition differed substantially across reports. Exposures included predominant or cumulative maternal milk intake, breastfeeding versus formula feeding, donor human milk, and proportions of human milk received during neonatal hospitalization. Accordingly, these reports were synthesized as related but non-equivalent nutritional exposures. Among neonatal MRI studies, Ottolini et al. [81] reported that predominantly breast-milk-fed very-low-birth-weight infants had larger total brain, amygdala–hippocampal, and cerebellar volumes at term-equivalent age, together with differences in white-matter microstructural measures, compared with predominantly formula-fed infants. Sullivan et al. [88] similarly identified associations between breast-milk exposure and measures of cortical maturation at term-equivalent age. At the functional-network level, Niu et al. [80] reported differences in dynamic functional connectivity and temporal network organization between breastfed and formula-fed preterm infants. Longer-term MRI evidence was provided by reports derived from the DoMINO parent trial/follow-up cohort. Sato et al. [87], Xu et al. [94], and Bando et al. [64] evaluated different nutritional exposures and imaging outcomes within the same parent population and were, therefore, not interpreted as three independent replications. Collectively, these reports examined relationships between early feeding exposure and later white-matter microstructure, cortical measures, and brain regions relevant to processing speed. Not all comparisons indicated more favorable imaging findings with greater or exclusive human-milk exposure. In the randomized NEOVASC ancillary analysis, Mitterer et al. [77] found no clear differences in structural brain growth or diffusion measures between a prolonged exclusive human milk-based diet and earlier introduction of bovine-derived products. Thus, the randomized contrast did not reproduce a generalized structural or microstructural advantage of prolonged exclusive human-milk-based feeding.

#### 3.3.2. Measured Nutrient and Bioactive Composition of Maternal Milk

Two reports from the same Brigham maternal-milk cohort investigated measured milk composition rather than human-milk exposure as a feeding proxy. Bell et al. [66] related repeated point-of-care measurements of milk macronutrient content to neonatal MRI and reported positive associations of higher measured energy and protein intake with total, cortical gray-matter, and white-matter volumes. Atayde et al. [63], using the same parent cohort, evaluated naturally occurring lactoferrin intake and reported positive associations with total and gray-matter volumes. Because these reports originated from a shared prospective cohort, their findings were considered complementary analyses of different milk components rather than independent confirmation of a generalized human-milk effect. Accordingly, quantitative pooling was not considered defensible for human-milk exposures because exposure definitions differed in source (maternal versus donor milk), dose and duration, comparator feeding, measured nutrient composition, fortification practices, imaging domain, and assessment age. In addition, several reports originated from shared parent cohorts, further reducing the number of independent populations available for any single contrast.

#### 3.3.3. Protein, Energy and Overall Nutritional Adequacy

##### Protein and Energy Intake

Several observational reports evaluated protein and/or energy delivery in relation to structural, microstructural, or functional brain development. The exposure definitions were heterogeneous and included cumulative macronutrient intake, enteral-to-parenteral protein ratios, and nutritional intake during specific early postnatal windows. Duerden et al. [70], from the PIANO cohort, reported an association between higher early protein intake and later functional connectivity and neurocognitive measures. Henkel et al. [73], using the CINEPS cohort, examined the early enteral-to-parenteral protein ratio in relation to brain volumes and somatic growth, thereby addressing route and proportional delivery rather than absolute protein exposure. Toppe et al. [89] examined early nutrition in relation to deep-gray-matter and lateral-ventricular volumes at term-equivalent age. Because these reports differed in exposure metrics, developmental timing, imaging outcome, and age at MRI, their effect estimates were not considered directly exchangeable.

##### Multinutrient Regimens and Nutritional Status

Two reports from the Utrecht nutrition-regimen cohort evaluated the consequences of implementation of a more intensive nutritional regimen and were treated as related analyses of a shared parent population. Van Beek et al. [93] reported larger brain volumes following implementation of the revised regimen, whereas Hortensius et al. [75] evaluated nutritional intake in relation to white-matter integrity and neurodevelopment. These findings, therefore, represent complementary structural and microstructural analyses within the same cohort rather than independent replication. Other observational reports evaluated broader nutritional adequacy or growth-related exposures, including Naseh et al., Valdes et al., and Qing et al. [79,86,92]. Because growth can lie on the causal pathway between nutrient intake and brain development, associations based on growth or nutritional status were interpreted separately from direct nutrient exposure contrasts. Pooling was not undertaken because no three independent reports shared a sufficiently comparable protein or energy exposure, comparator, imaging endpoint, and developmental time point. In particular, total brain volume, regional volumetry, diffusion measures, functional connectivity, and later-childhood MRI were not treated as interchangeable outcomes.

#### 3.3.4. DHA, ARA and Fatty-Acid Status

##### Observational Fatty-Acid Status

Observational evidence relating to fatty-acid status to brain development was distinguished from randomized supplementation effects. Hortensius et al. [74] reported positive associations between serum DHA concentrations and several regional brain volumes in extremely preterm infants. Because circulating DHA concentrations may reflect maternal supply, clinical condition, enteral tolerance, and concurrent parenteral and enteral nutrition, this analysis was interpreted as an observational exposure–outcome association rather than evidence of a treatment effect.

##### Randomized ARA/DHA Supplementation

Two included reports evaluated randomized ARA/DHA supplementation but arose from different parent trials and assessed different imaging domains. In the ImNuT trial, Moltu et al. [78] reported higher fractional anisotropy and lower radial diffusivity in several white-matter tracts among supplemented infants, consistent with differences in microstructural maturation at term-equivalent age. In the Mega Donna Mega trial, Hellström et al. [72] reported differences in brain volumes at term, including greater white-matter volume following combined ARA and DHA supplementation. Although both randomized contrasts concerned ARA/DHA provision, differences in supplementation regimen, parent trial, and imaging endpoint precluded direct quantitative pooling.

#### 3.3.5. Parenteral Lipid-Emulsion Strategies

Four included reports compared or evaluated parenteral lipid-emulsion strategies using neuroimaging outcomes [62,68,69,82]. Costa et al. [69] evaluated a randomized treatment contrast and reported greater cerebellar volume at term-equivalent age with a multicomponent lipid emulsion than with a soybean-based emulsion (24.0 ± 9.6 versus 18.2 ± 6.2 cm^3^; *p* = 0.045); after correction for postmenstrual age, corresponding values were 22.3 ± 6.5 versus 16.9 ± 7.2 cm^3^ (*p* = 0.027). The RoB 2 overall judgment for this contrast was high risk of bias.

Ottolini et al. [82], in a non-randomized historical comparison, also assessed term-equivalent-age brain development after multicomponent versus soybean-based intravenous lipid exposure. Mean cerebellar volumes were 20.7 ± 4.6 and 19.5 ± 5.2 cm^3^, respectively; the adjusted estimate for cerebellar volume did not demonstrate a clear between-group difference [β = 0.6 cm^3^, 95% CI −1.3 to 2.4]. The report was judged to be at serious risk of bias using ROBINS-I.

The remaining two historical comparisons assessed different imaging constructs. Al-Mouqdad et al. [62] used the Kidokoro scoring system and reported less favorable term-equivalent-age MRI findings among infants exposed to SMOFlipid, including differences in maturation/injury-related components; the study was judged at serious risk of bias. Caruggi et al. [68] assessed Total Maturation Score at term-equivalent age. Although the unadjusted comparison favored the soybean-emulsion group [median TMS 13 (3) versus 12 (2), *p* = 0.007], this difference was not retained after false-discovery-rate correction (*p* = 0.09); the study was also judged at serious risk of bias.

Thus, effect direction was not consistent across the four reports and depended partly on the imaging construct examined. Costa et al. and Ottolini et al. [69,82] were the only reports providing sufficiently similar cerebellar volumetric outcomes; because only two studies met the prespecified comparability criteria, meta-analysis was not performed. Al-Mouqdad et al. and Caruggi et al. measured Kidokoro and Total Maturation Scores [62,68], respectively, and these outcomes were not considered quantitatively commensurable with regional volumetry.

#### 3.3.6. Other Specific Nutritional Exposures

A smaller number of reports examined nutritional exposures that could not be grouped into sufficiently populated causal categories. Manninen et al. [76] evaluated carnitine intake and circulating levels in relation to postnatal growth and brain size at term. Other reports addressed individualized or route-specific nutritional exposures that were not directly comparable with the human-milk, fatty-acid, or lipid-emulsion groups described above. These findings were retained in the synthesis but were not combined across exposure classes because the underlying nutritional constructs and target effects differed.

#### 3.3.7. Quantitative Cranial Ultrasound Evidence

Two independent observational cohorts evaluated nutritional exposure using quantitative cranial ultrasonography. Boscarino et al. [67] (n = 109) assessed cerebral growth between days 3–7 and day 28. Enteral energy intake was positively associated in multivariable analyses with growth of the left caudate head (β = 0.227) and cerebellar vermis height (β = 0.415), whereas parenteral energy intake was inversely associated with growth of the right and left caudate heads (β = −0.164 and −0.228, respectively) and transverse cerebellar diameter (β = −0.849).

Laccetta et al. [51] (n = 42) evaluated term-equivalent-age white-matter echogenicity using quantitative pixel-brightness analysis. Parenteral energy intake was positively correlated with right and left relative echogenicity of the choroid plexus (RECP) measures (r = 0.413, *p* = 0.007 and r = 0.422, *p* = 0.005), as was parenteral amino-acid intake (r = 0.438, *p* = 0.004 and r = 0.446, *p* = 0.003); these relationships persisted in multivariable analyses.

Despite both studies using CUS, quantitative pooling was not appropriate because Boscarino et al. measured serial anatomical growth during the neonatal period, whereas Laccetta et al. quantified term-equivalent-age white-matter echogenicity. The two studies, therefore, addressed different imaging domains and target outcomes.

#### 3.3.8. Neurodevelopmental Follow-Up and Relationship to Imaging

A subset of included reports assessed neurodevelopment in addition to neuroimaging. Follow-up age and instruments varied substantially across reports, ranging from assessments in early childhood to school-age outcomes. Because this review required an eligible neuroimaging outcome, neurodevelopmental assessments were treated as complementary clinical outcomes rather than as a separate eligibility domain.

Three distinct relationships were, therefore, distinguished in the synthesis: nutritional exposure–imaging associations, imaging–neurodevelopment associations, and studies in which both imaging and neurodevelopment were reported following the same nutritional exposure. The latter items were not interpreted as demonstrating mediation unless a formal mediation analysis had been performed. Similarly, an association between a nutritional exposure and both an imaging measure and a developmental outcome was not considered evidence that the imaging measure constituted a validated surrogate endpoint for neurodevelopment.

Quantitative synthesis of neurodevelopmental outcomes was not undertaken because follow-up ages, assessment instruments, nutritional contrasts, parent cohorts, and analytic approaches were insufficiently comparable.

### 3.4. Certainty of Evidence

Overall certainty of evidence ranged from very low to low across the nutrition–neuroimaging questions evaluated. No outcome-specific body of evidence was judged to provide moderate- or high-certainty evidence. Certainty was limited principally by risk of bias, imprecision, sparse independent replication, and, for several nutritional exposures, heterogeneity in exposure definition and neuroimaging outcome.

Among randomized intervention evidence, ARA/DHA supplementation provided low-certainty evidence for differences in term-equivalent-age white-matter microstructure and brain volumetry, respectively. The randomized comparison of a prolonged exclusive human-milk-based diet also provided low-certainty evidence for structural and diffusion MRI outcomes. The randomized lipid-emulsion comparison provided very-low-certainty evidence for cerebellar volume, principally because of high risk of bias and imprecision.

Certainty was generally very low for observational nutritional exposures and for non-randomized intervention comparisons when serious risk of bias, limited information size, or lack of independent replication reduced confidence in the estimated associations. In particular, the evidence concerning parenteral lipid-emulsion strategies remained very uncertain because the randomized and non-randomized evidence differed in methodological limitations and imaging constructs, and the direction of findings was not uniform. Quantitative CUS evidence was also of very low certainty, with only two independent observational cohorts addressing different outcome domains—serial anatomical growth and term-equivalent-age tissue echogenicity—and, therefore, not constituting direct replication of a common imaging outcome. Detailed outcome-specific GRADE judgments and reasons for rating certainty are provided in Table S2.

As nutritional exposures, MRI time scans (birth, first days of life, TEA, childhood, adolescence), MRI outcomes and study designs (observationals/RCTs) are too heterogenous, adopting or not neurodevelopmental scales (Bayley, etc.), meta-analysis was not feasible per all outcomes (i.e., neurodevelopmental outcomes at 7 years of age; brain morphometrics and maturational outcomes in case of protein supplementation). Parenteral lipid emulsions represented the closest candidate for quantitative synthesis. Costa et al. and Ottolini et al. [69,84] reported cerebellar volume at term-equivalent age using comparable volumetric outcomes. However, only two studies met the predefined comparability criteria for this outcome. Al-Mouqdad et al. and Caruggi et al. [62,68] assessed brain development using the Kidokoro score and Total Maturation Score, respectively, and were, therefore, not quantitatively pooled with volumetric data. Accordingly, lipid-emulsion studies were synthesized using a structured Synthesis Without Meta-Analysis (SWiM).

## 4. Discussion

### 4.1. Principal Findings

This systematic review identifies a growing but methodologically heterogeneous literature linking early nutrition with brain development in preterm infants. Human milk exposure, early macronutrient provision, lipid composition, and selected supplementation strategies were associated in several studies with measures of brain volume, white-matter maturation, and connectivity. These findings are biologically plausible and clinically relevant, but they should not be interpreted as evidence that any single nutritional strategy directly improves brain structure or long-term neurodevelopment. The formal certainty assessment further qualifies these findings. Across outcome-specific nutrition–neuroimaging questions, certainty ranged from very low to low, indicating that apparently favorable imaging associations should not be interpreted as established nutritional effects. Randomized evidence for ARA/DHA supplementation and prolonged exclusive human-milk-based feeding provided comparatively greater confidence than most observational exposure evidence, although certainty remained low because of methodological limitations and imprecision. Thus, the principal contribution of the current evidence is the identification of biologically and clinically plausible signals that warrant confirmation rather than definitive evidence for a specific neuroprotective nutritional strategy.

### 4.2. Interpretation of Evidence

Human-milk-related evidence was directionally suggestive of associations with several dimensions of brain development, but its interpretation was limited by substantial heterogeneity in exposure definition. Maternal milk, donor milk, predominant breastfeeding, measured milk composition, and exclusive human-milk-based diets represent related but biologically and methodologically non-equivalent exposures. Moreover, several findings originated from shared parent populations, reducing the amount of independent replication. The absence of a generalized structural or microstructural advantage in the randomized NEOVASC contrast further cautions against attributing observational associations to human milk per se. Evidence concerning lipid provision and fatty-acid composition was similarly heterogeneous. Observational associations between circulating DHA or cumulative lipid exposure and brain measures cannot be interpreted as equivalent to randomized supplementation effects, because achieved nutrient status may reflect illness severity, feeding tolerance, maternal supply, and concurrent nutrition. Randomized ARA/DHA trials provided evidence of differences in selected structural and microstructural domains, but variation in supplementation regimens and imaging endpoints prevents inference of a uniform treatment effect. Findings for intravenous lipid formulations were additionally inconsistent across volumetric and maturation/injury-based imaging constructs. Overall, the literature supports the biological plausibility of associations between early lipid and energy provision and preterm brain maturation, but the magnitude and causal nature of these associations, as well as the optimal composition, dose, and timing of nutritional interventions, remain uncertain. Future trials should prespecify neuroimaging endpoints, distinguish total lipid dose from fatty-acid composition, report concurrent protein and energy intake, and measure circulating fatty-acid profiles. Longitudinal follow-up is also essential to determine whether early differences in regional volumes, diffusion metrics, or spectroscopy translate into durable neurodevelopmental benefit. The limited CUS evidence suggests that quantitative ultrasonography may extend bedside assessment beyond conventional lesion detection toward measures of cerebral growth and tissue echogenicity. However, the two included studies addressed different imaging constructs and nutritional questions, preventing any inference of a common nutrition-related CUS phenotype. Prospective validation and standardized acquisition and analysis pipelines are required before such quantitative measures can be interpreted across centers. Beyond the included evidence, recent methodological developments illustrate how quantitative CUS could evolve toward increasingly automated image analysis. For example, a recent study applied an active-contour (“snake”) model [97] to automatically delineate regions of interest and quantify grayscale features associated with white-matter damage in preterm infants. Although this study did not investigate nutritional exposures and was, therefore, not included in the present review, such approaches may complement the quantitative pixel-brightness methodology used by Laccetta et al. by improving reproducibility and standardization of CUS-derived imaging features. Whether these techniques can characterize nutrition-related trajectories of white-matter maturation requires prospective validation.

### 4.3. Why Results Remain Heterogeneous

As previously underscored by Gilbreath et al., 2024 [46] in *Nutrients*, human milk does have a slight positive impact on the structural development of the preterm infant brain. However, further diets with distinct macronutrient compositions are characterized by more conflicting results. Specifically, results remain heterogeneous because MRI can detect differences in brain growth, microstructure, and connectivity before neurodevelopmental testing becomes stable and sufficiently predictive. The MRI effect size depends on the design, population, MRI time scan and protocols as well as assessed outcomes. Especially when including DTI and connectivity dimensions, studies are more center-dependent and less generalizable. Conversely, CUS offers complementary bedside information and allows repeated evaluation, although its sensitivity and standardization differ from MRI. Heterogeneity may result in multiple definitions of human milk (maternal breast milk, donor milk, etc.), MRI timing, different nutrients, gestational age, statistical adjustments, clinical severity and different follow-ups. This variation limits direct comparison. Similarly, studies of DHA and omega-3 fatty acids differed in formulation, dose, treatment window, background nutrition, and primary endpoint. Favorable findings in selected imaging domains, therefore, require confirmation in adequately powered trials with prespecified imaging and developmental outcomes.

### 4.4. Biological Mechanisms

Nutrition may influence brain maturation through substrate availability, membrane composition, growth-factor signaling, energy metabolism, inflammation, oxidative stress, and the gut–brain axis. Observations in infants with extrauterine head-growth restriction or delayed feeding suggest that metabolic and inflammatory biomarkers may help characterize vulnerability. However, mediation pathways remain incompletely defined, and mechanistic claims should be tested in longitudinal multimodal studies. External mechanistic evidence also suggests that perinatal omega-3 exposure may modulate oxidative-stress pathways; for example, Bruschi et al. reported differences in glutathione-related proteins and redox status in cord blood from pregnancies complicated by threatened preterm labor. Although this study did not assess neonatal nutrition or neuroimaging outcomes and was, therefore, outside this review’s eligibility criteria, it provides biological context for a potential link between fatty-acid exposure and oxidative vulnerability. For instance, Bruschi et al. [98] conducted a proteomic analysis for evaluating the fatty acid omega-3 supplementation in women with diagnosed threatening preterm labor. The ELISA array highlighted that the glutathione synthetase was more abundant in cord blood from untreated preterm infants compared to the other conditions. Assay of free SH-groups showed that serum of preterm subjects was oxidized. Data suggested that preterm subjects suffered from oxidative stress, which was lower in the treated cohort.

Nonetheless, causal inference cannot be directly established. Accordingly, we propose the RIGEL (Roadmap for Imaging-Guided Enhancement of Neurodevelopment) framework considering the current evidence summarized in this review.

### 4.5. Future Directions. The RIGEL Framework: Enhancing Brain Development and Maturation

**Figure 5:**
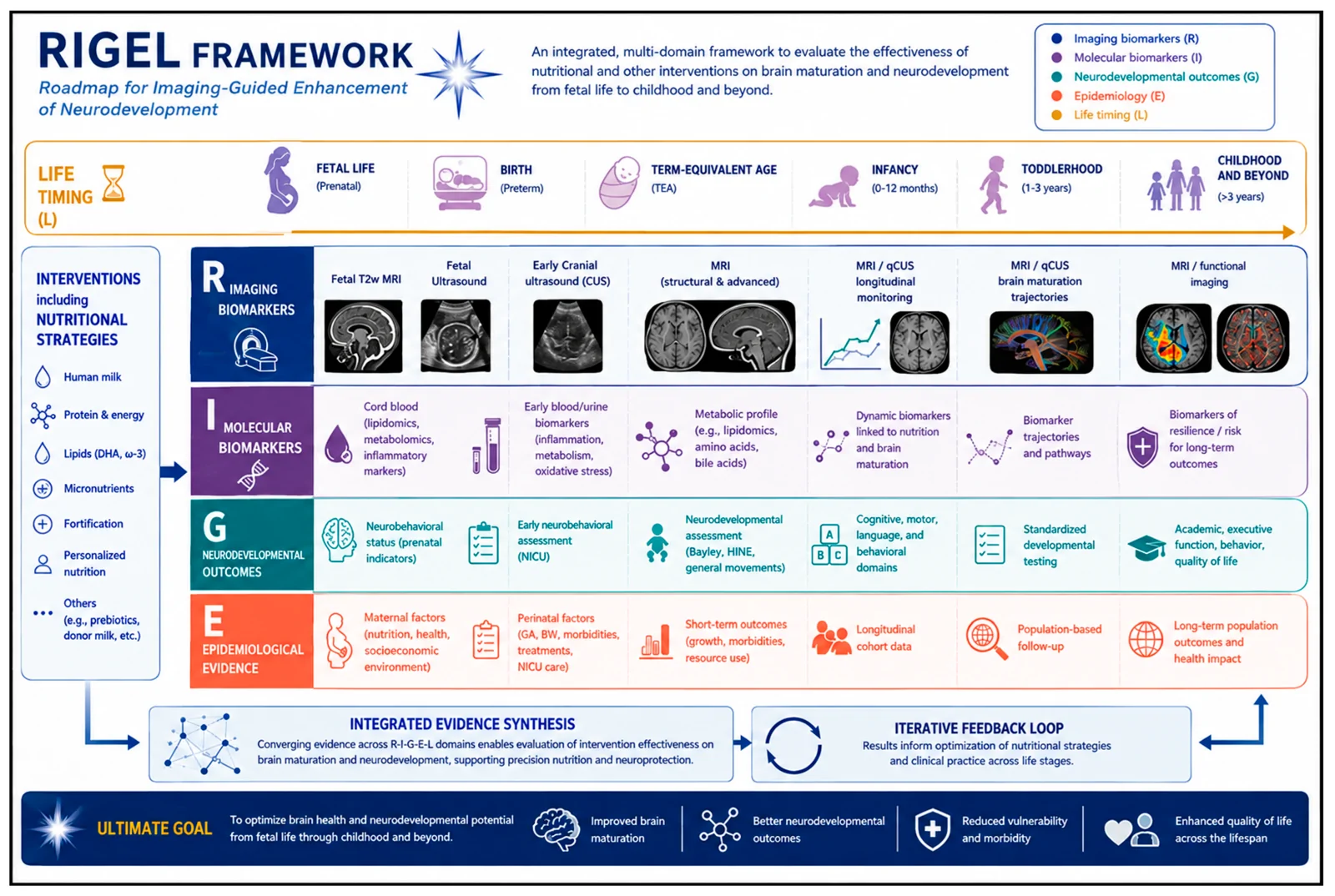
The RIGEL framework.

Neonatal nutritional care remains to be further optimized and standardized. The Italian Society of Neonatology (SIN), the Italian Society of Pediatric Surgery (SICP), and the Italian Society of Pediatric Nutrition (SINUPE) underscore the importance of personalizing nutritional strategies according to available evidence, careful monitoring, and expert consensus [99]. Building on previous neonatal and neuroimaging studies [70,100,101,102], we introduce the RIGEL framework (Roadmap for Imaging-Guided Enhancement of Neurodevelopment) in Figure 5. RIGEL is a hypothesis-generating framework, in which neuroimaging measures are investigated alongside molecular biomarkers and neurodevelopmental outcomes to determine whether convergent multimodal measures can improve the characterization of nutrition-related developmental trajectories in preterm infants. Imaging outcomes should not replace functional endpoints; rather, their relationship with clinically meaningful developmental outcomes requires prospective longitudinal validation. Current nutritional studies often evaluate growth, neuroimaging, and neurodevelopment separately, whereas RIGEL proposes their prospective integration within the same study design.

The broader extension of RIGEL from fetal life to childhood and beyond should be regarded as conceptual rather than as a conclusion derived from the studies included in this systematic review. Such a life-course application would require independent validation across developmental stages and populations. Nutrition may influence brain maturation not only through substrate availability but also through modulation of inflammatory and metabolic pathways, suggesting that molecular and metabolic biomarkers may complement neuroimaging in characterizing brain vulnerability and nutrition-related developmental trajectories [8,103,104].

## 5. Strengths and Limitations

This review provides an updated synthesis of MRI and quantitative CUS evidence published from 2020 to 2026, incorporating parent-cohort linkage, target-contrast-specific risk-of-bias assessment, and a formal assessment of the certainty of evidence. Several limitations should nevertheless be acknowledged. First, quantitative meta-analysis was not performed because of substantial heterogeneity in nutritional exposures and interventions, neuroimaging outcomes, developmental time points, and study populations, together with limited independent replication across sufficiently comparable studies. Second, the review protocol was not prospectively registered; rather, the methodology was adapted and expanded from the PROSPERO-registered review by Ottolini and Keller [37]. Third, study screening and data extraction were primarily performed by a single reviewer, which may have increased the risk of selection or extraction errors. Fourth, the literature search was restricted to three databases, and relevant studies indexed elsewhere may, therefore, have been missed. Fifth, risk-of-bias assessment was not performed independently by two human reviewers. Although AI-supported tools were used to structure and cross-check judgments, the absence of duplicate human assessment may have introduced classification error or interpretive bias. Sixth, the GRADE certainty-of-evidence assessment was added during revision and was not prespecified in the original review methodology. Finally, several eligible reports originated from the same or partially overlapping parent cohorts; although these relationships were explicitly identified and overlapping reports were not treated as independent replication in the synthesis, the effective amount of independent evidence was, therefore, smaller than the number of included reports might suggest.

## Figures and Tables

**Table 1:** Parent studies with included reports.

Family ID	Parent Study/Cohort	Included Reports
F01	Previous randomized lipid-emulsion trial	Costa 2024 [69]
F02	DoMINO	Sato 2021; Xu 2022; Bando 2024 [64,87,94]
F03	Utrecht nutrition-regimen cohort	van Beek 2020; Hortensius 2021b [77,95]
F04	Uppsala very-preterm cohort	Naseh 2025 [79]
F05	Boscarino neonatal nutrition/CUS cohort	Boscarino 2021 [67]
F06	Sapienza quantitative-CUS cohort	Laccetta 2024 [51]
F07	PIANO Study	Duerden 2021; Poppe 2025 [70,85]
F08	Gaslini historical lipid cohort	Caruggi 2026 [68]
F09	PreBaby/Kuopio cohort	Manninen 2022 [76]
F10	ImNuT	Moltu 2024 [78]
F11	NEOVASC	Mitterer 2026 [77]
F12	Essen preterm cohort	Toppe 2023 [89]
F13	Brigham maternal-milk cohort	Bell 2022; Atayde 2024 [63,66]
F14	CINEPS	Henkel 2024 [73]
F15	Children’s National umbrella	Ottolini 2020; Ottolini 2021; Ottolini 2024; Ottolini 2026; Basu 2025 [65,81,82,83,84]
F16	Sullivan preterm/term imaging cohort	Sullivan 2023 [88]
F17	NCT02760472 lipid RCT	Hortensius 2021a [74]
F18	Mega Donna Mega	Hellström 2026 [72]
F19	Zhejiang cohort A	Niu 2020 [80]
F20	Zhejiang cohort B	Zhang 2022 [95]
F21	KSMC historical lipid cohort	Al-Mouqdad 2024 [62]
F22	Granada VLBW longitudinal cohort	Uberos 2026 [91]
F23	University of Florida very-preterm cohort	Valdes 2024 [92]
F24	Xiangya preterm cohort	Qing 2025 [86]

## Data Availability

All data are reported in the article body and Supplementary Material. Further information is available in the respective articles (References Section).

## Supplementary Materials

The following supporting information can be downloaded at: https://www.mdpi.com/article/10.3390/nu18203392/s1, Figure S1: ATLAS preliminary assessment; Figure S2: Traffic light plots (RoB assessment for ROBINS-I, RoB 2 and NOS); Table S1: Data Extraction of 33 included articles; Table S2: GRADE Evidence Profil, GRADE Rules & Sources and RoB Inputs. References [51,62,63,64,65,66,67,68,69,70,72,73,74,75,76,77,78,79,80,81,82,83,84,85,86,87,88,89,91,92,93,94,95] are cited in the Supplementary Materials.
