# Supplementary material for: The Preterm Brain Is Also What It Eats: A Systematic Review of Nutrition and Neuroimaging Outcomes in Preterm Infants
Source: Nutrients. 2026 Oct 16;18(20):3392. doi: 10.3390/nu18203392 (PMC13708710; doi:10.3390/nu18203392)
Supplement: Supplementary file 1 [file nutrients-18-03392-s001.zip › SUPPLEMENTARY MATERIAL - Nutrients I Review 2026_REVISED2.pdf]

SUPPLEMENTARY MATERIAL

First scan date: 20th July 2025 16:35 (PubMed, Scopus and CINAHL); second update: 24th July 2026 (PubMed, Scopus and CINAHL). Third update: 28th July 2026 (PubMed, Scopus and CINAHL. Fourth update: 4th September 2026 (PubMed, Scopus and CINAHL).

Note that search strings should be run on the respective databases as follows without modifying them in order to visualize bibliographic outputs correctly.

PubMed
("Breast Feeding"[Mesh] OR "Breast Feeding" OR "Infant Nutritional Physiological Phenomena"[Mesh] OR "Milk, Human"[Mesh] OR "Milk, Human" OR "Lipids"[Mesh] OR Lipids OR "Fatty Acids, Unsaturated"[Mesh] OR "Fatty Acids, Unsaturated" OR "Arachidonic Acid"[Mesh] OR "Arachidonic Acid" OR "Dietary Supplements"[Mesh] OR "Dietary Supplements" OR "Dietary Fats"[Mesh] OR "Dietary Fats" OR "Dietary Fats, Unsaturated"[Mesh] OR "Dietary Fats, Unsaturated" OR "Nutritive Value"[Mesh] OR "Nutritive Value" OR "Nutritional Support"[Mesh] OR "Nutrition Therapy"[Mesh] OR "Nutritional Status"[Mesh] OR "Nutrition Assessment"[Mesh] OR macronutrients[tiab] OR nutrients[tiab] OR "long chain polyunsaturated fatty acids"[tiab] OR "docosahexaenoic acid"[tiab] OR diet[tiab]) AND ("Magnetic Resonance Imaging"[Mesh] OR "Diffusion Magnetic Resonance Imaging"[Mesh] OR "Image Processing, Computer-Assisted"[Mesh] OR MRI[tiab] OR "magnetic resonance imaging"[tiab] OR "resting-state MRI"[tiab] OR "resting state magnetic resonance imaging"[tiab] OR "diffusion tensor imaging"[tiab] OR ("volume analysis"[tiab] AND "magnetic resonance imaging"[tiab]) OR "Ultrasonography"[Mesh] OR "cranial ultrasound"[tiab] OR "cranial ultrasonography"[tiab] OR "cranial sonography"[tiab] OR "head ultrasound"[tiab] OR CUS[tiab] OR cUS[tiab]) AND ("Premature Birth"[Mesh] OR "Infant, Extremely Premature"[Mesh] OR "preterm birth"[tiab] OR "periviable birth"[tiab] OR "premature babies"[tiab] OR preemie*[tiab] OR "term equivalent"[tiab] OR premature[tiab] OR neonat*[tiab] OR infant*[tiab] OR preterm[tiab]) AND ("Brain"[Mesh] OR brain[tiab])

CINAHL SEARCH STRING

(
(MH "Breast Feeding+")
OR (MH "Infant Nutritional Physiological Phenomena+")
OR (MH "Milk, Human+")
OR (MH Lipids+)
OR (MH "Fatty Acids, Unsaturated+")
OR (MH "Arachidonic Acid+")
OR (MH "Dietary Supplements+")
OR (MH "Dietary Fats+")
OR (MH "Dietary Fats, Unsaturated+")
OR (MH "Nutritive Value+")
OR (MH "Nutritional Support+")
OR (MH "Nutrition Therapy+")
OR (MH "Nutritional Status+")
OR (MH "Nutrition Assessment+")
OR (TI lipids OR AB lipids)
OR (TI macronutrients OR AB macronutrients)
OR (TI nutrients OR AB nutrients)
OR (TI "long chain polyunsaturated fatty acids" OR AB "long chain polyunsaturated fatty acids")
OR (TI "docosahexaenoic acid" OR AB "docosahexaenoic acid")
OR (TI "dietary fats" OR AB "dietary fats")
OR (TI diet OR AB diet)
)
AND

(
(MH "Magnetic Resonance Imaging+")
OR (MH "Diffusion Magnetic Resonance Imaging+")
OR (MH "Image Processing, Computer-Assisted+")
OR (TI MRI OR AB MRI)
OR (TI "magnetic resonance imaging" OR AB "magnetic resonance imaging")
OR (TI "resting-state MRI" OR AB "resting-state MRI")
OR (TI "resting state magnetic resonance imaging" OR AB "resting state magnetic resonance imaging")
OR (TI "diffusion tensor imaging" OR AB "diffusion tensor imaging")
OR ((TI "volume analysis" OR AB "volume analysis") AND (TI "magnetic resonance imaging" OR AB "magnetic resonance imaging"))
OR (MH Ultrasonography+)
OR (TI "cranial ultrasound" OR AB "cranial ultrasound")
OR (TI "cranial ultrasonography" OR AB "cranial ultrasonography")
OR (TI "cranial sonography" OR AB "cranial sonography")
OR (TI "head ultrasound" OR AB "head ultrasound")
OR (TI CUS OR AB CUS)
OR (TI cUS OR AB cUS)
)
AND
(
(MH "Premature Birth+")
OR (MH "Infant, Extremely Premature+")
OR (TI "preterm birth" OR AB "preterm birth")
OR (TI "periviable birth" OR AB "periviable birth")
OR (TI "premature babies" OR AB "premature babies")
OR (TI preemie* OR AB preemie*)
OR (TI "term equivalent" OR AB "term equivalent")
OR (TI premature OR AB premature)
OR (TI neonat* OR AB neonat*)
OR (TI infant* OR AB infant*)
OR (TI preterm OR AB preterm)
)
AND
(
(MH Brain+)
OR (TI brain OR AB brain)
)

SCOPUS (BASIC SEARCH)

(
"Breast Feeding"
OR "Infant Nutritional Physiological Phenomena"
OR "Milk, Human"
OR Lipids
OR "Fatty Acids, Unsaturated"
OR "Arachidonic Acid"
OR "Dietary Supplements"
OR "Dietary Fats"
OR "Dietary Fats, Unsaturated"
OR "Nutritive Value"
OR "Nutritional Support"
OR "Nutrition Therapy"
OR "Nutritional Status"

OR "Nutrition Assessment"
OR lipids
OR macronutrients
OR nutrients
OR "long chain polyunsaturated fatty acids"
OR "docosahexaenoic acid"
OR "dietary fats"
OR diet
)
AND
(
"Magnetic Resonance Imaging"
OR "Diffusion Magnetic Resonance Imaging"
OR "Image Processing, Computer-Assisted"
OR MRI
OR "magnetic resonance imaging"
OR "resting-state MRI"
OR "resting state magnetic resonance imaging"
OR "diffusion tensor imaging"
OR ("volume analysis" AND "magnetic resonance imaging")
OR Ultrasonography
OR "cranial ultrasound"
OR "cranial ultrasonography"
OR "cranial sonography"
OR "head ultrasound"
OR CUS
OR cUS
)
AND
(
"Premature Birth"
OR "Infant, Extremely Premature"
OR "preterm birth"
OR "periviable birth"
OR "premature babies"
OR preemie*
OR "term equivalent"
OR premature
OR neonat*
OR infant*
OR preterm
)
AND
(
Brain
OR brain
)

SCOPUS (ADVANCED SEARCH)

INDEXTERMS("Breast Feeding")
OR INDEXTERMS("Infant Nutritional Physiological Phenomena")
OR INDEXTERMS("Milk, Human")
OR INDEXTERMS(Lipids)
OR INDEXTERMS("Fatty Acids, Unsaturated")

```
OR INDEXTERMS("Arachidonic Acid")
OR INDEXTERMS("Dietary Supplements")
OR INDEXTERMS("Dietary Fats")
OR INDEXTERMS("Dietary Fats, Unsaturated")
OR INDEXTERMS("Nutritive Value")
OR INDEXTERMS("Nutritional Support")
OR INDEXTERMS("Nutrition Therapy")
OR INDEXTERMS("Nutritional Status")
OR INDEXTERMS("Nutrition Assessment")
OR TITLE-ABS(lipids)
OR TITLE-ABS(macronutrients)
OR TITLE-ABS(nutrients)
OR TITLE-ABS("long chain polyunsaturated fatty acids")
OR TITLE-ABS("docosahexaenoic acid")
OR TITLE-ABS("dietary fats")
OR TITLE-ABS(diet)
)
AND
(
INDEXTERMS("Magnetic Resonance Imaging")
OR INDEXTERMS("Diffusion Magnetic Resonance Imaging")
OR INDEXTERMS("Image Processing, Computer-Assisted")
OR TITLE-ABS(MRI)
OR TITLE-ABS("magnetic resonance imaging")
OR TITLE-ABS("resting-state MRI")
OR TITLE-ABS("resting state magnetic resonance imaging")
OR TITLE-ABS("diffusion tensor imaging")
OR (TITLE-ABS("volume analysis") AND TITLE-ABS("magnetic resonance imaging"))
OR INDEXTERMS(Ultrasonography)
OR TITLE-ABS("cranial ultrasound")
OR TITLE-ABS("cranial ultrasonography")
OR TITLE-ABS("cranial sonography")
OR TITLE-ABS("head ultrasound")
OR TITLE-ABS(CUS)
OR TITLE-ABS(cUS)
)
AND
(
INDEXTERMS("Premature Birth")
OR INDEXTERMS("Infant, Extremely Premature")
OR TITLE-ABS("preterm birth")
OR TITLE-ABS("periviable birth")
OR TITLE-ABS("premature babies")
OR TITLE-ABS(preemie*)
OR TITLE-ABS("term equivalent")
OR TITLE-ABS(premature)
OR TITLE-ABS(neonat*)
OR TITLE-ABS(infant*)
OR TITLE-ABS(preterm)
)
AND
(
INDEXTERMS(Brain)
OR TITLE-ABS(brain)
)
```

Table S1: Data Extraction of 33 included articles

Included papers [1–33]

1. Al-Mouqdad MM, Alshaikh B, Sumaily HH, Alodhaidan NA, AlMahmoud L, Almotiri AA, et al. Association between SMOFlipid and impaired brain development on term-equivalent age brain magnetic resonance imaging in very preterm infants. BMC Pediatr. 2024 Oct 29;24(1):686. doi:10.1186/s12887-024-05153-8

2. Atayde AMP, Kapoor NR, Cherkerzian S, Olson I, Andrews C, Lee ACC, et al. Lactoferrin intake from maternal milk during the neonatal hospitalization and early brain development among preterm infants. Pediatr Res. 2024 Jul;96(1):159–64. doi:10.1038/s41390-023-03002-5

3. Bando N, Sato J, Vandewouw MM, Taylor MJ, Tomlinson C, Unger S, et al. Early nutritional influences on brain regions related to processing speed in children born preterm: A secondary analysis of a randomized trial. J Parenter Enteral Nutr. 2024 Oct;48(7):778–86. doi:10.1002/jpen.2669

4. Basu SK, Ottolini KM, Kapse KJ, Ngwa J, Hui SCN, Andescavage N, et al. Early parenteral lipid intake supports cerebellar neurometabolism at term-age in preterm infants. J Perinatol. 2025 Dec;45(12):1735–44. doi:10.1038/s41372-025-02336-8

5. Bell KA, Cherkerzian S, Drouin K, Matthews LG, Inder TE, Prohl AK, et al. Associations of Macronutrient Intake Determined by Point-of-Care Human Milk Analysis with Brain Development among very Preterm Infants. Children. 2022 Jun 29;9(7):969. doi:10.3390/children9070969

6. Boscarino G, Di Chiara M, Cellitti R, De Nardo MC, Conti MG, Parisi P, et al. Effects of early energy intake on neonatal cerebral growth of preterm newborn: an observational study. Sci Rep. 2021 Sep 16;11(1):18457. doi:10.1038/s41598-021-98088-4

7. Caruggi S, Calandrino A, Corradi B, Battaglini M, Massirio P, Vinci F, et al. Do different lipid emulsions affect brain development? A single-center retrospective analysis. Front Nutr. 2026 Feb 4;13:1723062. doi:10.3389/fnut.2026.1723062

8. Costa S, Cocca C, D'Apolito G, De Gisi A, Fattore S, Tataranno ML, et al. Effects of a Multicomponent Lipid Emulsion on Brain Volumes in Extremely Low Birth Weight Infants. Am J Perinatol. 2024 May;41(S 01):e1813–9. doi:10.1055/a-2077-2551

9. Duerden EG, Thompson B, Poppe T, Alsweiler J, Gamble G, Jiang Y, et al. Early protein intake predicts functional connectivity and neurocognition in preterm born children. Sci Rep. 2021 Feb 18;11(1):4085. doi:10.1038/s41598-021-83125-z

10. Hellström W, Lundgren P, Nilsson AK, Nilsson S, Hård AL, Sjöbom U, et al. Effect of enteral arachidonic acid and docosahexaenoic acid supplementation on brain volumes at term in preterm infants: a secondary outcome analysis of a randomised controlled trial. Arch Dis Child Fetal Neonatal Ed. 2026 Jul;111(4):F344–51. doi:10.1136/archdischild-2024-328292

11. Henkel RD, Fu TT, Barnes-Davis ME, Sahay RD, Liu C, Hill CD, et al. Effects of Early Enteral to Parenteral Protein Ratios on Brain Volume and Somatic Growth in Very Low Birth Weight Infants. The Journal of Pediatrics. 2024 Dec;275:114253. doi:10.1016/j.jpeds.2024.114253

12. Hortensius LM, Hellström W, Sävman K, Heckemann RA, Björkman-Burtscher IM, Groenendaal F, et al. Serum docosahexaenoic acid levels are associated with brain volumes in extremely preterm born infants. Pediatr Res. 2021 Dec;90(6):1177–85. doi:10.1038/s41390-021-01645-w

13. Hortensius LM, Janson E, Van Beek PE, Groenendaal F, Claessens NHP, Swanenburg De Veye HFN, et al. Nutritional Intake, White Matter Integrity, and Neurodevelopment in Extremely Preterm Born Infants. Nutrients. 2021 Sep 27;13(10):3409. doi:10.3390/nu13103409

14. Laccetta G, De Nardo MC, Cellitti R, Di Chiara M, Tagliabracci M, Parisi P, et al. Quantitative Evaluation of White Matter Injury by Cranial Ultrasound to Detect the Effects of Parenteral Nutrition in Preterm Babies: An Observational Study. J Imaging. 2024 Sep 10;10(9):224. doi:10.3390/jimaging10090224

15. Manninen S, Silvennoinen S, Bendel P, Lankinen M, Schwab US, Sankilampi U. Carnitine Intake and Serum Levels Associate Positively with Postnatal Growth and Brain Size at Term in Very Preterm Infants. Nutrients. 2022 Nov 9;14(22):4725. doi:10.3390/nu14224725

16. Mitterer W, Hochmayr C, Waltner-Romen M, Sappler M, Hammerl M, Gatterer L, et al. Effects of a Prolonged Exclusive Human Milk-Based Diet on Structural and Functional Brain Maturation in Very Preterm Infants: An Ancillary Analysis of the NEOVASC Trial. Nutrients. 2026 Apr 22;18(9):1321. doi:10.3390/nu18091321

17. Moltu SJ, Nordvik T, Rossholt ME, Wendel K, Chawla M, Server A, et al. Arachidonic and docosahexaenoic acid supplementation and brain maturation in preterm infants; a double blind RCT. Clinical Nutrition. 2024 Jan;43(1):176–86. doi:10.1016/j.clnu.2023.11.037

18. Naseh N, Vaz TF, Ferreira H, Moreira NC, Hellström-Westas L, Ahlsson F, et al. Impact of early nutrition on brain development and neurocognitive outcomes in very preterm infants. Pediatr Res. 2025 Aug;98(2):593–8. doi:10.1038/s41390-025-03964-8

19. Niu W, Xu X, Zhang H, Zhang Y, Ni S, Liu T, et al. Breastfeeding improves dynamic reorganization of functional connectivity in preterm infants: a temporal brain network study. Med Biol Eng Comput. 2020 Nov;58(11):2805–19. doi:10.1007/s11517-020-02244-3

20. Ottolini KM, Andescavage N, Kapse K, Jacobs M, Limperopoulos C. Improved brain growth and microstructural development in breast milk–fed very low birth weight premature infants. Acta Paediatrica. 2020 Aug;109(8):1580–7. doi:10.1111/apa.15168

21. Ottolini KM, Andescavage N, Kapse K, Jacobs M, Murnick J, VanderVeer R, et al. Early Lipid Intake Improves Cerebellar Growth in Very Low-Birth-Weight Preterm Infants. J Parenter Enteral Nutr. 2021 Mar;45(3):587–95. doi:10.1002/jpen.1868

22. Ottolini KM, Basu SK, Ngwa J, Kapse K, Murnick J, Cook KM, et al. Donor human milk and structural brain development in very preterm infants. Pediatr Res. 2026 Jun;99(7):2640–8. doi:10.1038/s41390-025-04539-3

23. Ottolini KM, Ngwa J, Basu SK, Kapse K, Liggett M, Murnick J, et al. Brain development using a multicomponent intravenous lipid emulsion in preterm infants. BMC Pediatr. 2024 Dec 30;24(1):847. doi:10.1186/s12887-024-05330-9

24. Poppe T, Tottman AC, Gamble GD, Jiang Y, Silva AE, Nguyen L, et al. Neonatal Nutrition and Brain Structure at 7 Years in Children Born Very Preterm. JAMA Netw Open. 2025 Jan 24;8(1):e2456080. doi:10.1001/jamanetworkopen.2024.56080

25. Qing Z, Wan L, He X, Chen P. The influence of early nutrition intake and clinical factors on the brain development of preterm infants with intrauterine growth restriction. Transl Pediatr. 2025 Oct;14(10):2520–32. doi:10.21037/tp-2025-432

26. Sato J, Vandewouw MM, Bando N, Ng DVY, Branson HM, O’Connor DL, et al. Early nutrition and white matter microstructure in children born very low birth weight. Brain Communications. 2021 Apr 5;3(2):fcab066. doi:10.1093/braincomms/fcab066

27. Sullivan G, Vaher K, Blesa M, Galdi P, Stoye DQ, Quigley AJ, et al. Breast Milk Exposure is Associated With Cortical Maturation in Preterm Infants. Annals of Neurology. 2023 Mar;93(3):591–603. doi:10.1002/ana.26559

28. Toppe F, Rasche T, Weiss C, Schock A, Felderhoff-Müser U, Müller H. Relationship between early nutrition and deep gray matter and lateral ventricular volumes of preterm infants at term-equivalent age. World J Pediatr. 2023 May;19(5):460–8. doi:10.1007/s12519-022-00657-8

29. Uberos J, Carrasco-Solis M, Laynez-Rubio C, Nieto-Ruiz A, Ruiz-López A, Contreras-Chova F, et al. Impact of early neonatal nutrition on neurodevelopment and the limbic system in very low birth weight infants. Br J Nutr. 2026 Feb 14;135(3):286–96. doi:10.1017/S0007114525105874

30. Valdes C, Nataraj P, Kisilewicz K, Simenson A, Leon G, Kang D, et al. Impact of Nutritional Status on Total Brain Tissue Volumes in Preterm Infants. Children. 2024 Jan 18;11(1):121. doi:10.3390/children11010121

31. Van Beek PE, Claessens NHP, Makropoulos A, Groenendaal F, De Vries LS, Counsell SJ, et al. Increase in Brain Volumes after Implementation of a Nutrition Regimen in Infants Born Extremely Preterm. The Journal of Pediatrics. 2020 Aug;223:57-63.e5. doi:10.1016/j.jpeds.2020.04.063

32. Xu J, Shin J, McGee M, Unger S, Bando N, Sato J, et al. Intake of mother’s milk by very-low-birth-weight infants and variation in DNA methylation of genes involved in neurodevelopment at 5.5 years of age. The American Journal of Clinical Nutrition. 2022 Oct;116(4):1038–48. doi:10.1093/ajcn/nqac221

33. Zhang Y, Deng Q, Wang J, Wang H, Li Q, Zhu B, et al. The impact of breast milk feeding on early brain development in preterm infants in China: An observational study. Duerden E, editor. PLoS ONE. 2022 Nov 21;17(11):e0272125. doi:10.1371/journal.pone.0272125

ATLAS Eligibility criteria

To perform an ATLAS Score Assessment, studies have to be explicitly adopting a quantitative analysis approach (radiomics; AI pipelines applied to imaging – possibly in integration with clinical biomarkers) in accordance with the population, exposure, comparator and outcome defined in this systematic review (see Methodology section of the main paper). Being composed of four domains (A1 – AI applied to imaging; A2 – Radiomic pipeline and clinical integration. A3 Dataset and Resources; A4 Clinical Synthesis (i.e. what the concerning study adds to the current clinical and radiological knowledge). The ATLAS score is computed automatically through its developed Google Colab Notebook extrapolating from eligible PDFs relevant expressions in compliance or not with the current standard checklists (CLAIM; METRICS; RQS, CLEAR; etc.)

Figure S1: ATLAS preliminary assessment

Reference of ATLAS framework

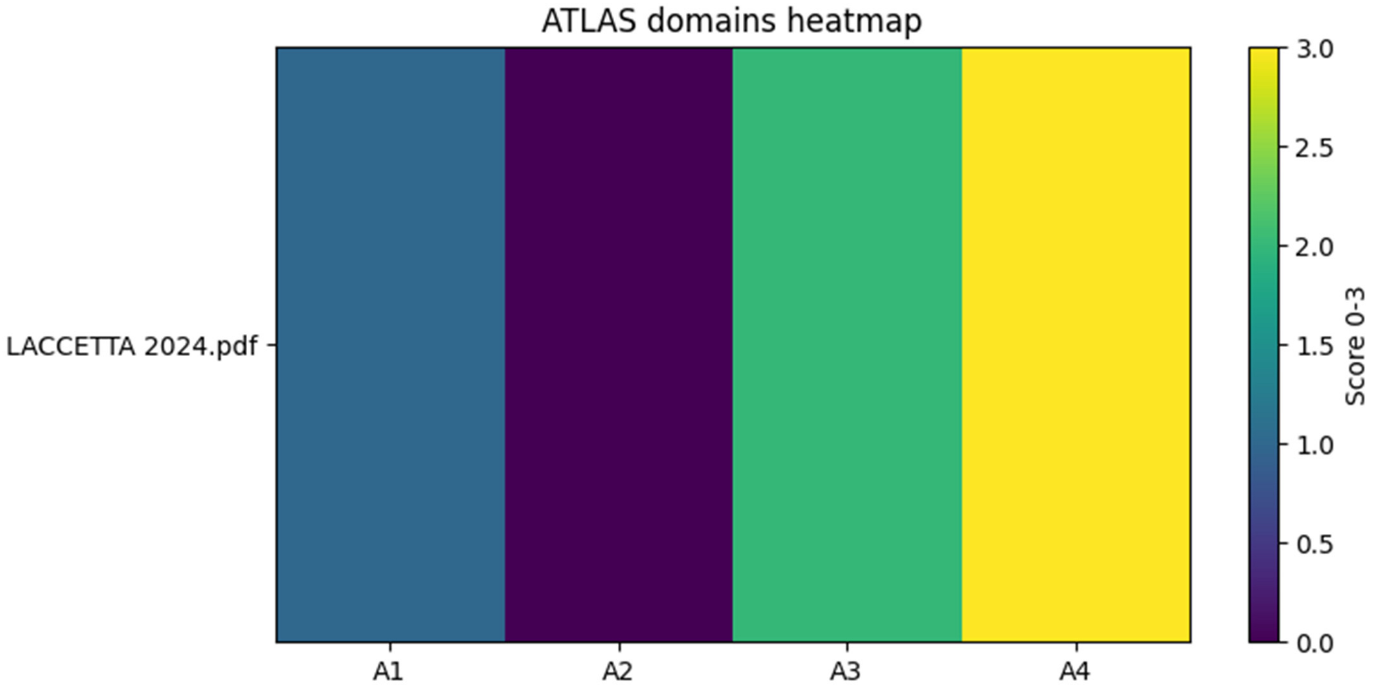

Risk of bias plots (Traffic Light type). Generated with BOOTHIE tool and checked manually reading the original PDF full-texts.

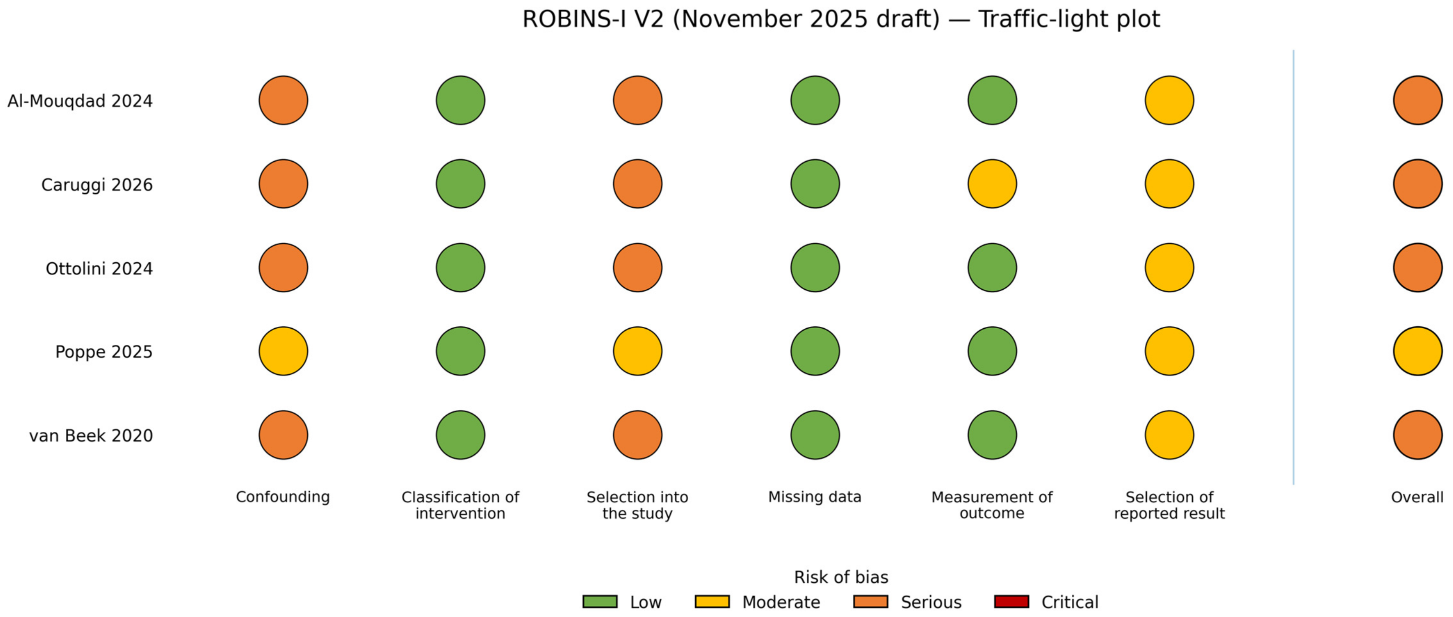

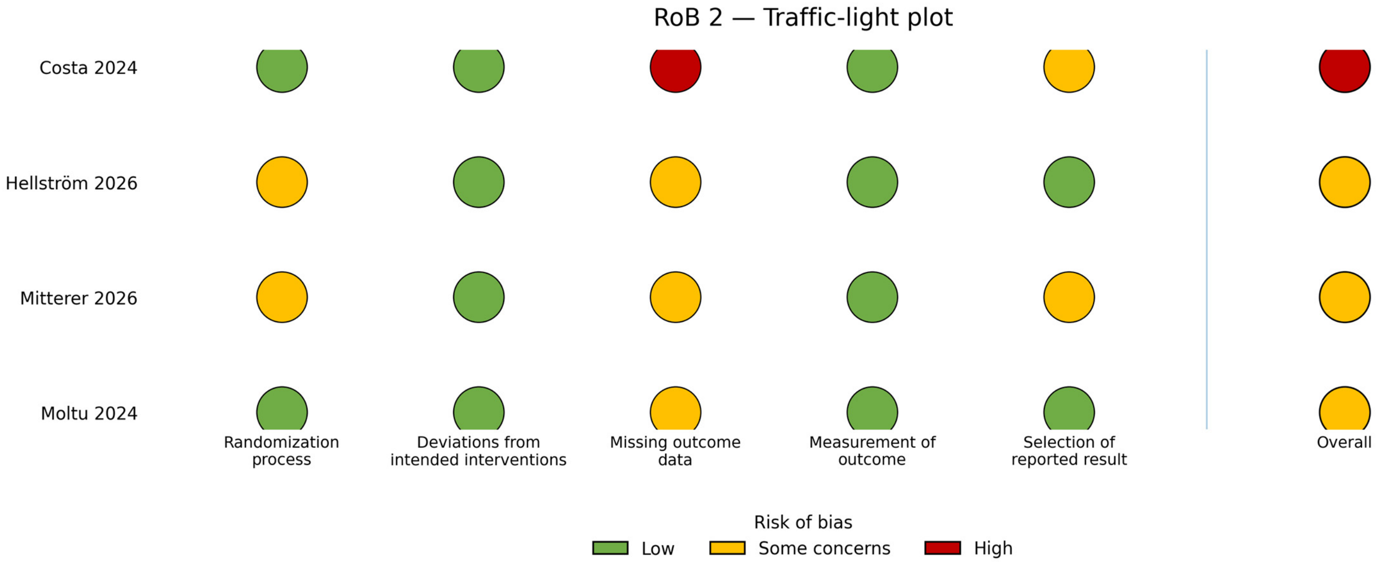

Newcastle–Ottawa Scale — Star matrix (traffic-light style)

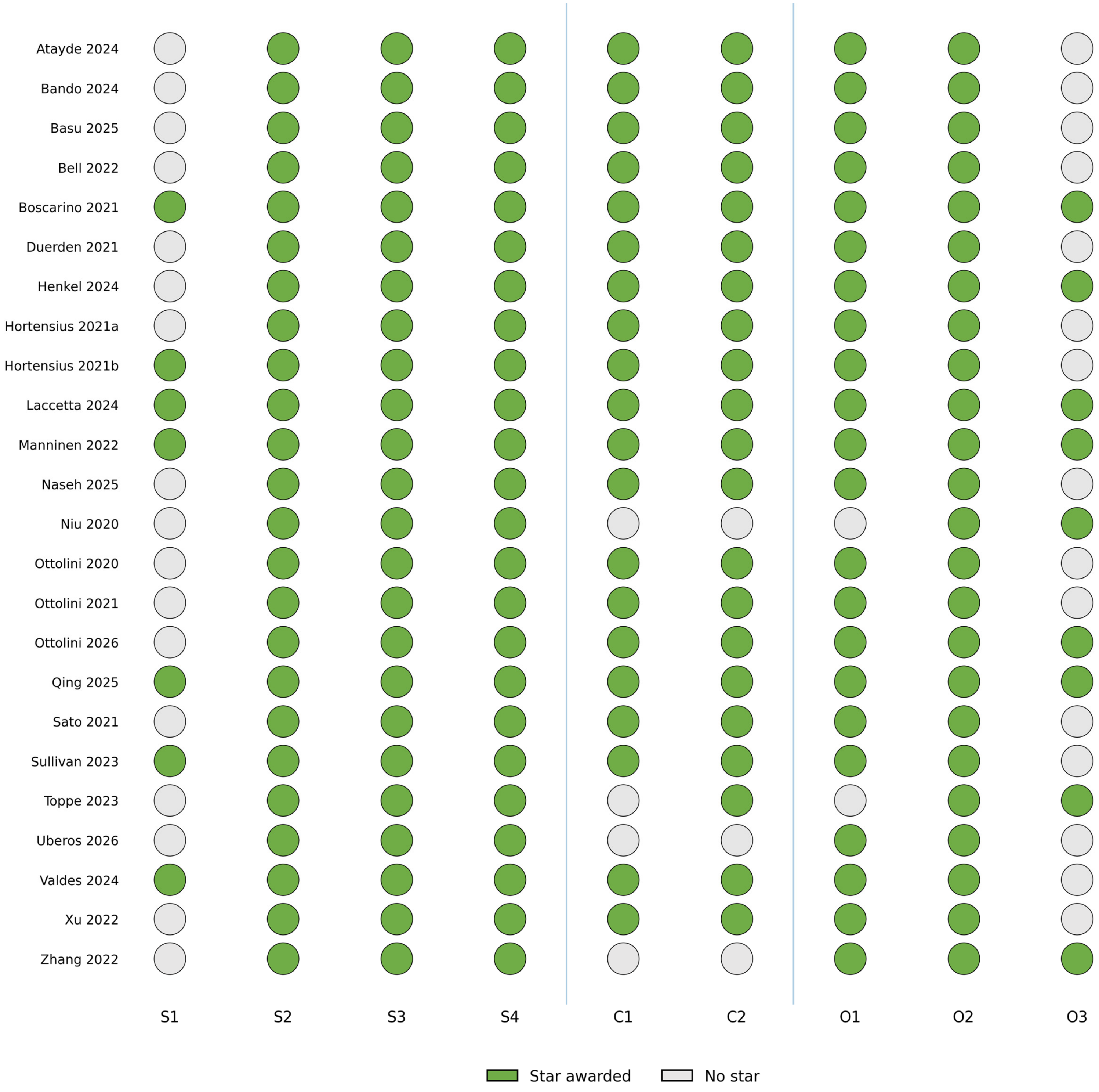

Data Extraction Document

33 records

Fields included: first author; year of publication; Nutritional interventions; Anthropometric features and growth trajectories; MRI results; Key neurodevelopmental outcomes; Country.

Precision Note for "Key neurodevelopmental outcomes" is reported in full the contents of the CSV column "Key neurodevelopmental results". Missing values in the CSV remain empty. The first author is extracted from the first element of the Authors field, without changing its spelling.

1. Lisa M Hortensius (2021)

**First author**	Lisa M Hortensius
**Year of publication**	2021
**Nutritional interventions**	- Parenteral lipid intervention:<br><br>- SMOFlipid: fish-oil-containing emulsion with 15% fish oil and omega-3 LCPUFAs; 22/48 infants (46%)<br><br>- Clinoleic: olive-oil-based emulsion; 26/48 infants<br><br>- Timing/dose: started as soon as possible after birth; final lipid dose 2–3 g/kg/day<br><br>- Enteral nutrition:<br><br>- Timing: minimal enteral feeding started within 3 hours after birth and volume was gradually increased<br><br>- Mother's own milk: used as an enteral milk source<br><br>- Donor milk: used as an enteral milk source<br><br>- Formula: not mentioned as an enteral feeding source<br><br>- Fortification: individual fortification was used<br><br>- Advancement: full enteral feeding (>150 ml/kg/day) was reached after a median of 16.0 days (IQR 9.0)<br><br>- Number of sessions: not mentioned; the paper describes ongoing parenteral nutrition and gradually advanced enteral feeding rather than a count of discrete nutritional sessions
**Anthropometric features and growth trajectories**	- Anthropometric features: Mean birth weight was 787 g (SD 212); median birth-weight SDS was −0.69 (IQR 1.0); median change in weight SDS from birth to MRI was −0.38 (IQR 1.4); and median weight SDS at MRI was −1.0 (IQR 1.4)<br><br>- Head circumference: The parent trial reported no between-treatment differences in weight, length, or head circumference, but this paper does not report head-circumference values or a head-circumference growth trajectory for the analyzed cohort

	- Growth trajectories: Change in weight SDS from birth to MRI was examined as a growth trajectory measure but was excluded from all final brain-volume models because it was not significant<br><br>- Growth-adjusted models: Models for total brain, cortical gray matter, and cerebellar volumes adjusted for postmenstrual age at MRI, significant brain injury, and weight SDS at MRI; deep gray matter and white matter models retained weight SDS at MRI, while ventricular models did not retain a significant growth covariate<br><br>- Interpretation: The positive DHA–brain-volume associations were estimated after accounting for concurrent weight status in the applicable models; they should not be interpreted as evidence of a DHA effect on weight gain or head growth
**MRI results**	- MRI acquisition: 3-Tesla MRI at term-equivalent age; median postmenstrual age 43.0 weeks (IQR 2.93)<br><br>- MRI analysis population: 48 infants in the final analysis<br><br>- Volumetrics: Six regions were measured—total brain, white matter, cortical gray matter, deep gray matter, cerebellum, and lateral ventricles<br><br>- Mean volumes:<br><br>- Total brain: 383 (53) cm³<br><br>- White matter: 151 (18) cm³<br><br>- Cortical gray matter: 169 (32) cm³<br><br>- Deep gray matter: 23 (2.7) cm³<br><br>- Cerebellum: 29 (5.7) cm³<br><br>- Ventricles: 12 (4.9) cm³<br><br>- DHA AUC associations:<br><br>- Total brain volume: B = 7,966; 95% CI, 1,837–14,095; P = 0.012<br><br>- Cortical gray matter volume: B = 3.653; 95% CI, 0.251–7.055; P = 0.036<br><br>- Deep gray matter volume: B = 0.439; 95% CI, 0.091–0.787; P = 0.014<br><br>- Cerebellar volume: B = 0.932; 95% CI, 0.333–1.530; P = 0.003<br><br>- White matter volume: B = 3.373; 95% CI, 0.504–6.241; P = 0.022<br><br>- Ventricular volume: No association; B = −0.341; 95% CI, −1.231 to 0.549; P = 0.444<br><br>- AA AUC: No associations with any measured brain volume<br><br>- Adjustment: Regression models were corrected for postmenstrual age at MRI

**Key neurodevelopmental outcomes**	- Direct neurodevelopmental outcome/follow-up: Not reported; the study assessed MRI-derived brain volumes at TEA, which were described only as potential biomarkers for future development<br><br>- TEA brain-volume results: Higher serum DHA during the first 28 postnatal days was associated with larger total brain, cortical gray matter, deep gray matter, cerebellar, and white matter volumes; no association was found with ventricular volume<br><br>- AA and neurodevelopment-related brain volumes: No significant associations between AA AUC and any brain volumes<br><br>- Interpretation: The findings support an association between early postnatal DHA status and MRI brain volumes, but do not establish improved neurodevelopment or causality
**Country**	Sweden

2. Pauline and Van Beek

**First author**	Pauline and Van Beek
**Year of publication**	
**Nutritional interventions**	- Old regimen (cohort A): parenteral nutrition began on day 2 after birth; protein and lipids were increased to maximums of 2.6 g/kg and 1.7 g/kg, respectively, by day 5.old_protocol<br><br>- New regimen (cohort B): parenteral nutrition began as soon as possible after NICU admission; protein and lipids started at 1 g/kg/day on admission and increased to maximums of 3.5 g/kg/day and 4 g/kg/day, respectively, by day 3, conditional on triglycerides remaining below 116 mg/dL.new_protocol<br><br>- Enteral feeding: minimal enteral feeding was introduced shortly after birth, with daily increases beginning 24–48 hours after birth if tolerated.enteral_feeding<br><br>- Intervention duration and frequency: nutritional intake was administered and calculated daily during the first 28 postnatal days; a number of discrete intervention sessions was not reported.protocol_timing<br><br>- Mother's own milk: not separately reported; the paper refers to breast milk and used a calculated composition of 68 kcal, 1.0 g protein, and 4.0 g fat per 100 mL.milk_composition<br><br>- Donor milk: not mentioned.milk_type_reporting<br><br>- Formula: not mentioned.milk_type_reporting<br><br>- Fortification: breast milk was fortified with additional carbohydrates and proteins at 120 mL/kg/day in cohort A and at 100 mL/kg/day in cohort B.fortification_strategy<br><br>- Reported achieved intake: cohort B had higher protein intake (daily median

	3.5 vs 2.7 g/kg) and calorie intake (109 vs 104 kcal/kg/day), while lipid intake was similar (4.8 vs 4.8 g/kg/day).nutritional_intake
**Anthropometric features and growth trajectories**	- Head circumference: Not mentioned (the reported anthropometric variables were gestational age, birth weight, birth-weight z-score, and small-for-gestational-age status; head circumference was not provided)<br><br>- Baseline anthropometrics: Birth weight: 865 g in cohort A vs 875 g in cohort B, P = .87; birth-weight z-score: 0.31 ± 0.90 vs 0.23 ± 0.91, P = .56; small for gestational age: 21% vs 27%, P = .51<br><br>- Weight-growth trajectory: Relative somatic growth from birth to 30 weeks PMA was 13.1 g/kg/day in cohort B vs 10.0 g/kg/day in cohort A, P < .001<br><br>- Weight z-score trajectory: The decrease from birth was smaller in cohort B than cohort A at 30 weeks PMA (−0.61 vs −0.88, P = .002) and at TEA (−0.90 vs −1.21, P = .036)<br><br>- Growth trajectory metric: Growth velocity was calculated from net weight gain, the interval's starting weight, and the number of days; growth was also reported as change in weight z-score<br><br>- Growth-adjusted models: Not mentioned (somatic growth was compared using an independent t test, with no growth-adjusted model described)<br><br>- Interpretation: The enriched nutrition regimen was associated with improved weight gain and less decline in weight z-score, although infants remained underweight at TEA
**MRI results**	- At 30 weeks PMA, cohort B had larger total brain volume than cohort A 151.415 ± 16.380 vs 142.914 ± 16.666; mean difference 8.500 (95% CI 1.698 to 15.302); P = .015 and larger grey-matter volume 48.298 ± 6.722 vs 44.319 ± 6.275; mean difference 3.980 (95% CI 1.323 to 6.637); P = .004. Larger volumes were observed in 14 of 16 grey-matter regions and 7 subcortical regions, whereas white-matter volume was generally comparable between cohorts.<br><br>- At TEA, total brain, grey-matter, and white-matter volumes did not differ between cohorts.<br><br>- Caudate nucleus: cohort B was larger at TEA [3,658 ± 428 vs 3,507 ± 391; mean difference 152 (95% CI 20 to 283); P = .024].<br><br>- Serial MRI: no cohort differences were found in growth of total brain, overall grey matter, or overall white matter; the anterior cingulate gyrus showed greater growth in cohort A than cohort B (P = .012).<br><br>- Maturation pattern: white-matter structures increased approximately 1.7-fold and grey-matter structures approximately 3.6-fold from 30 weeks PMA to TEA; the cerebellum increased approximately 3.8-fold.
**Key neurodevelopmental outcomes**	- Early MRI brain volumes: At 30 weeks PMA, the enriched-nutrition cohort had significantly larger total brain and grey-matter volumes, with larger volumes in 14 of 16 grey-matter regions and 7 subcortical regions; white-matter volume was largely comparable.<br><br>- TEA brain volumes: No significant cohort differences were found in total

	brain, grey-matter, or white-matter volume; only the caudate nucleus was significantly larger in the enriched-nutrition cohort (mean difference 152; 95% CI, 20 to 283; P = .024).<br><br>- Brain growth from 30 weeks PMA to TEA: No between-cohort differences were found in total brain or overall grey- and white-matter growth.<br><br>- Growth pattern: White matter increased approximately 1.7-fold and grey matter approximately 3.6-fold; the cerebellum increased approximately 3.8-fold.<br><br>- Somatic growth: The enriched-nutrition cohort had greater weight gain and less decline in weight z-score through 30 weeks PMA and TEA.<br><br>- Neurodevelopmental outcome/follow-up: Not reported; long-term neurodevelopmental follow-up was recommended for future studies.
**Country**	The Netherlands

3. Basu S.K. (2025)

**First author**	Basu S.K.
**Year of publication**	2025
**Nutritional interventions**	- Timing and duration: Parenteral nutrition began on NICU admission; lipid was started within 24 hours of admission, and nutrition exposure was assessed from admission through the first 4 weeks of life<br><br>- Parenteral nutrition intervention: Amino acids 3 g/kg/day and dextrose 4–8 mg/kg/min were initiated at admission; 20% lipid emulsion began at 1 g/kg/day and protein/lipid doses were advanced by 1 g/kg/day as tolerated toward 3.5–4 g/kg/day protein and 3 g/kg/day lipid<br><br>- Parenteral lipid formulation: Soybean-oil Intralipid® was used initially; from 2019, this was changed to SMOFlipid®, containing soybean, medium-chain triglycerides, olive, and fish oil<br><br>- Number of sessions/days: Median 17 days of parenteral nutrition (IQR 10–23) and 24 days of enteral nutrition (IQR 16–27) during the first 4 weeks<br><br>- Mother's own milk: Primary enteral source for 25/48 infants (52%)<br><br>- Donor milk: Used when parents consented; donor human milk was reported for 17/48 infants (35%)<br><br>- Formula: A 24-kcal/oz preterm formula was used when human milk was unavailable; formula was reported for 6/48 infants (13%)<br><br>- Enteral timing and progression: Trophic feeds of 10–20 mL/kg/day began within 24 hours of life or when clinically stable and continued for 3–5 days; feeds then advanced by 20–30 mL/kg/day to a target of 160 mL/kg/day

	- Fortification: When enteral intake reached 80–100 mL/kg/day, a cow-milk-based fortifier was added to human milk to achieve 24 kcal/oz
**Anthropometric features and growth trajectories**	- Birth anthropometrics: weight 1104 ± 355 g; length 36.1 ± 4.6 cm; head circumference 25.0 ± 2.9 cm<br><br>- Term-age anthropometrics: weight 2539 ± 497 g; length 45.4 ± 2.6 cm; head circumference 32.6 ± 2.0 cm<br><br>- Small for gestational age: 8/48 infants (17%)<br><br>- Extra-uterine growth failure: 32/48 infants (67%); defined as a decrease in weight z-score greater than 1 from birth to term-equivalent age<br><br>- Growth trajectories: No serial growth trajectories were reported; the study only compared birth and term-age anthropometrics and classified extra-uterine growth failure<br><br>- Growth-adjusted models: Regression models considered GA, SGA status, PMA at MRI, extra-uterine growth failure, sex, nutrition source, BPD, PDA, infection, and NEC; head circumference was not reported as a model covariate or outcome
**MRI results**	- MRI/1H-MRS cohort: 48 preterm infants without structural brain injury; MRI acquired at mean 38.9 ± 1.9 weeks PMA<br><br>- Cerebellar metabolite concentrations: NAA 3.13 ± 1.12 i.u.; Glx 5.60 ± 2.71; choline 2.86 ± 0.57; creatine 5.26 ± 1.24; NAA/choline 1.10 ± 0.35<br><br>- Volumetrics: cerebellar volume 18.2 ± 4.0 cc; total brain volume 324 ± 47 cc-<br><br>MRI metabolite maturation: PMA at MRI positively correlated with cerebellar NAA, Cr, and NAA/Cho; GA at birth did not correlate with metabolite measurements<br><br>- Parenteral lipid associations: NAA, Rho = 0.40, p = 0.005; Glx, Rho = 0.28, p = 0.07; NAA/choline, Rho = 0.32, p = 0.028<br><br>- Adjusted associations: 4-week parenteral lipid intake was associated with NAA, B = 0.89, p = 0.003, and Glx, B = 1.72, p = 0.045; parenteral caloric intake was positively associated with NAA, B = 0.03, p = 0.042, and Glx, B = 0.09, p = 0.007<br><br>- Cerebellar volume: no significant correlations with parenteral nutrition parameters; enteral macronutrient intake was not significantly associated with cerebellar volume or metabolites<br><br>- Lipid formulation comparison: multi-component lipid was associated with borderline lower NAA than soybean lipid, but this did not meet the adjusted significance threshold
**Key neurodevelopmental outcomes**	- At TEA, higher parenteral lipid intake during the first 4 weeks was associated with higher cerebellar NAA and Glx concentrations in preterm infants without structural brain injury; adjusted associations were NAA B = 0.89 (p = 0.003) and Glx B = 1.72 (p = 0.045). Parenteral caloric intake was also positively associated with NAA and Glx. These were neurometabolic findings rather than direct developmental test results.

	- Neurodevelopmental outcome: Not measured/reported; the authors only note that higher NAA and NAA/choline have been associated with favorable neurodevelopmental outcomes in other studies.<br><br>- Follow-up: No long-term cognitive, social-behavioral, or post-discharge developmental follow-up was reported; the long-term impact was identified as requiring further investigation.
**Country**	United States

4. Agate M P Atayde (2024)

**First author**	Agate M P Atayde
**Year of publication**	2024
**Nutritional interventions**	- Intervention type: Observational exposure to naturally varying lactoferrin concentrations in maternal milk; no randomized nutritional supplementation was administered<br><br>- Mother's own milk: Mothers intended to provide exclusive maternal milk; maternal milk comprised 93.8% ± 18.0% of enteral intake at day of life 28<br><br>- Donor milk: Some infants may have received donor milk during the neonatal hospitalization, but the amount and timing were not reported<br><br>- Formula: Some infants may have received formula during the neonatal hospitalization, but the amount and timing were not reported<br><br>- Fortification: Milk samples were collected immediately before fortification and infant feeding; the fortifier type, dose, schedule, and duration were not reported<br><br>- Timing: Milk samples were obtained as close as possible to chronologic days 14 and 28, and lactoferrin concentrations were averaged across these two time points<br><br>- Number of sessions: Mothers were instructed to express milk at least 8 times daily using a double-electric pump, with additional hand expression; the number of infant feeding sessions was not reported<br><br>- Estimated lactoferrin exposure: Approximately 16 mg/kg/day in the low-lactoferrin group and 43 mg/kg/day in the high-lactoferrin group, based on 160 mL/kg/day enteral intake
**Anthropometric features and growth trajectories**	- Anthropometric features: Mean gestational age was 28.0 ± 2.2 weeks; mean birth weight was 1053 ± 376 g; Table 3 also reports birth weight z-score, birth length, and birth length z-score<br><br>- Head circumference: Not mentioned in the provided paper text or tables.<br><br>- Growth trajectories: Not reported; the study provides baseline size measures and evaluates brain volumes at term-equivalent age rather than longitudinal

	weight, length, or head-circumference trajectories.<br><br>- Growth-adjusted models: Model 1 adjusted for sex, gestational age, birth weight z-score, and postmenstrual age at MRI; Model 2 additionally adjusted for mean protein intake
**MRI results**	- MRI timing and method: Brain MRI was performed without sedation at term equivalent age (35.9–41.3 weeks postmenstrual age) in 36 infants<br><br>- MRI volumetric measures: MANTiS quantified total brain, cortical gray matter, deep gray matter, white matter, hippocampus, and cerebellum volumes<br><br>- Mean MRI volumes at term equivalent age:<br><br>- Total brain volume: 324 ± 36 cc<br><br>- Cortical gray matter: 134 ± 25 cc<br><br>- Deep gray matter: 23 ± 2 cc<br><br>- White matter: 133 ± 17 cc<br><br>- Cerebellum: 23 ± 4 cc<br><br>- Hippocampus: 3 ± 0.9 cc<br><br>- High versus low lactoferrin exposure, adjusted for sex, gestational age, birth-weight z-score, and postmenstrual age:<br><br>- Total brain volume: 43.9 cc larger (95% CI, 7.6 to 80.4)<br><br>- Cortical gray matter: 48.3 cc larger (95% CI, 12.1 to 84.6)<br><br>- Deep gray matter: 3.8 cc larger (95% CI, 0.7 to 7.0)<br><br>- Other regional volumes: No statistically significant differences were reported for white matter, hippocampus, or cerebellum in Model 1<br><br>- Additional adjustment for mean protein intake:<br><br>- Deep gray matter: 3.6 cc larger in the high versus low tertile (95% CI, 0.2 to 7.0)<br><br>- Cerebellum: 5.3 cc larger in the middle versus low tertile (95% CI, 1.4 to 9.2); 6.0 cc larger in the high versus low tertile (95% CI, −1.0 to 12.9)<br><br>- MRI brain-injury findings: Punctate signal abnormalities occurred in the white matter and cerebellum in 3 infants each (8.3%); grade 3 intraventricular hemorrhage occurred in 3 infants (8.3%), and grade 4 hemorrhage was absent
**Key neurodevelopmental outcomes**	- TEA neurodevelopmental outcome: At term equivalent age, high versus low lactoferrin exposure was associated with 43.9 cc larger total brain volume, 48.3 cc larger cortical gray matter volume, and 3.8 cc larger deep gray matter volume.<br><br>- Other regional brain volumes: No statistically significant between-group differences were reported.

	- Follow-up: No post-discharge neurodevelopmental follow-up outcome was reported; the outcome was quantitative brain MRI at term equivalent age.
**Country**	United States

5. Uberos J. (2026)

**First author**	Uberos J.
**Year of publication**	2026
**Nutritional interventions**	- Intervention type: Observational exposure to enteral and/or parenteral nutrition, with liquid, energy, protein, carbohydrate and lipid intakes recorded during the first week of life<br><br>- Timing: From the first day/first days of life through the first week; enteral nutrition was supplemented with parenteral nutrition when full enteral feeding could not be established<br><br>- Number of sessions: Not reported as discrete sessions; nutrition was administered and recorded daily<br><br>- Mother's own milk: 44/74 children (59·4%) received mother's milk feeding<br><br>- Donor milk: Not mentioned<br><br>- Formula: Formula composition was assessed using commercial reports, but the number receiving formula and the formula-feeding schedule were not reported<br><br>- Fortification strategy: Not mentioned; the paper states that breast-milk composition was determined using the Standardised Neonatal Growth and Nutrition Report checklist but does not describe a fortifier, dose or protocol<br><br>- Parenteral nutrition: From April 2011, first-day parenteral nutrition included amino acids 2 g/kg and glucose 5 mg/kg/min; before then, amino acids were not given during the first hours of life<br><br>- Lipid emulsion: SMOFlipid replaced Intralipid as the standard parenteral-nutrition emulsion in July 2016
**Anthropometric features and growth trajectories**	- Birth anthropometry: Mean birth weight was 1234 (SD 346) g, with birth-weight z-score −0·31 (SD 0·88); mean gestational age was 29·2 (SD 1·9) weeks<br><br>- Head circumference: Recorded retrospectively and converted to z-scores using Fenton growth charts, but no numerical head-circumference results were reported<br><br>- Fetal growth restriction: 12/74 children (16·2%); defined by estimated fetal weight or abdominal circumference below the 3rd percentile, or below the 10th percentile with abnormal Doppler findings

	- Childhood anthropometry: At mean age 11·1 (SD 2·9) years, mean weight was 40·0 (SD 16·0) kg, height 145·1 (SD 18·2) cm and BMI 18·2 (SD 3·6) kg/m²<br><br>- Growth trajectories: Not reported; the paper provides birth and childhood anthropometric summaries but no serial measurements or trajectory analyses<br><br>- Growth-adjusted models: Structural MRI regressions were adjusted for child age and BMI. After adjustment, lipid intake remained associated with left and right thalamic and nucleus accumbens volumes
**MRI results**	- MRI volumetrics were available for 74 school-aged children born prematurely.<br><br>- Lipid intake and limbic-system volume:<br><br>- Left thalamus: b = 50·7; P = 0·014<br><br>- Right thalamus: b = 47·4; P = 0·018<br><br>- Left nucleus accumbens: b = 5·52; P = 0·023<br><br>- Right nucleus accumbens: b = 5·02; P = 0·031<br><br>- Energy intake: no statistically significant adjusted volumetric associations; left posterior cingulate cortex b = 1·09, P = 0·109<br><br>- Protein intake: no statistically significant adjusted volumetric associations; right isthmus cingulate cortex b = 8·46, P = 0·544<br><br>- Other regions: no significant macronutrient associations for the amygdala, parahippocampal cortex, hippocampus, or anterior/rostral cingulate regions.<br><br>- Thalamic volume and cognition: bilateral thalamic volumes correlated significantly with verbal comprehension and visuospatial scores.
**Key neurodevelopmental outcomes**	- Follow-up: School-age assessment at a mean age of 11•1 (SD 2•9) years; WISC-V was completed by 157 children and MRI by 74<br><br>- Neurodevelopmental outcome: Lower lipid intake in the first neonatal week was associated with lower verbal comprehension, visual-spatial scores and total IQ; lipid intake below 1•2 g/kg/d was associated with impaired verbal and visuospatial abilities<br><br>- Brain–cognition findings: Lipid intake was associated with larger bilateral thalamus and nucleus accumbens volumes; thalamic volumes correlated significantly with verbal comprehension and visuospatial scores<br><br>- TEA/autism: Not assessed as an outcome; severe autism spectrum disorder was an exclusion criterion
**Country**	Spain

6. Ottolini KM (2020)

**First author**	Ottolini KM
**Year of publication**	2020
**Nutritional interventions**	- Timing and duration: Daily parenteral and enteral nutrition intake was recorded from NICU admission until term-equivalent age; no discrete number of nutritional-intervention sessions was reported.<br><br>- Parenteral nutrition: Protein was started at 3 g/kg/day on NICU admission; 20% soybean-oil lipid emulsion was started at 1 g/kg/day within 24 hours of admission, with protein and lipid increased by 1 g/kg/day as tolerated to goals of 4 and 3 g/kg/day, respectively.<br><br>- Enteral feeding: Trophic feeds began at 10–20 mL/kg/day within 24 hours of life or once clinically stable, advanced daily by 20–30 mL/kg/day as tolerated, to a goal of 160 mL/kg/day.<br><br>- Mother's own milk: Maternal breast milk was used in 30/67 infants (44.8%).<br><br>- Donor milk: Donor breast milk was available from April 2015 when maternal supply was insufficient, with parental consent, until 34 weeks' corrected postmenstrual age; 13/67 infants (19.4%) received donor breast milk.<br><br>- Formula: Infants without available maternal breast milk were transitioned to formula at 34 weeks' corrected postmenstrual age; 24/67 infants (35.8%) received formula.<br><br>- Fortification: Breast milk was fortified with liquid bovine-based human milk fortifier to 22 kcal/oz at 80 mL/kg/day and to 24 kcal/oz at 100 mL/kg/day.
**Anthropometric features and growth trajectories**	- Cohort: 67 VLBW infants; mean gestational age 27.60 ± 2.51 weeks and mean birth weight 0.96 ± 0.31 kg<br><br>- Head circumference: Mean birth head circumference was 24.08 ± 2.63 cm<br><br>- Growth status: 7 infants (10.4%) were small for gestational age; average weight gain was 20.65 ± 4.65 g/day-<br><br>Growth trajectories: The study reported average daily weight gain rather than serial anthropometric growth trajectories; models treated average daily weight gain as a covariate<br><br>- Growth-adjusted interpretation: Associations between early lipid intake and cerebellar volume were estimated independently of gestational age at birth, postmenstrual age at MRI, and average daily weight gain<br><br>- Sensitivity to additional adjustment: Further adjustment for significant medical comorbidities attenuated the lipid–cerebellar associations to β = 0.025–0.07 with P = .05–.07
**MRI results**	- MRI assessment: Term-equivalent MRI was analyzed in 67 VLBW infants without structural brain injury; regional volumes included cortical gray matter, deep gray matter, white matter, amygdala-hippocampus, cerebellum, brainstem, and total brain volume

	- Cerebellar volume, 2-week lipid intake: positively associated; β = 0.096; P = .007, adjusted for gestational age at birth, postmenstrual age at MRI, and average daily weight gain<br><br>- Cerebellar volume, 4-week lipid intake: positively associated; β = 0.036; P = .006, with the same adjustments<br><br>- Brainstem volume, 4-week lipid intake: positively associated; β = 0.004; P = .049<br><br>- Total brain volume, 4-week lipid intake: positive but not statistically significant; β = 0.209; P = .08<br><br>- Cerebellar volume, 4-week energy intake: positively associated; β = 0.006; P = .02<br><br>- Carbohydrate and protein intake: no significant associations with total or regional brain volumes<br><br>- Additional medical-covariate adjustment: attenuated the lipid–cerebellar, energy–cerebellar, and lipid–brainstem associations
**Key neurodevelopmental outcomes**	- TEA brain MRI outcome: In 67 VLBW preterm infants without structural brain injury, greater cumulative lipid intake was associated with significantly larger cerebellar volume at term-equivalent age after adjustment for gestational age, postmenstrual age at MRI, and weight gain; 2-week intake: β = 0.096, P = .007, and 4-week intake: β = 0.036, P = .006. Four-week lipid intake was also associated with larger brainstem volume (β = 0.004, P = .049), while total brain volume showed only a nonsignificant trend. Greater cerebellar and brainstem volume at TEA with higher early lipid intake<br><br>- Other macronutrients: Four-week energy intake was associated with larger cerebellar volume (β = 0.006, P = .02); carbohydrate and protein intake were not significantly associated with total or regional brain volumes. Energy association; no carbohydrate/protein association<br><br>- Neurodevelopmental follow-up: Not mentioned; no later developmental or behavioral follow-up outcome was reported in the supplied paper. The paper calls for future research on long-term neurodevelopment
**Country**	Not mentioned (the paper identifies the study site as a level IV NICU and Children's National Medical Center but does not state the country)

7. Jingxiong Xu (2022)

**First author**	Jingxiong Xu
**Year of publication**	2022
**Nutritional interventions**	- Study scope: No new nutritional intervention was assigned in this 2022 follow-up; it analyzed in-hospital feeding exposure from participants in the original Donor Milk for Improved Neurodevelopmental Outcomes trial<br><br>- Timing: During the initial hospitalization of VLBW infants

	- Frequency/sessions: Feed type and volume were recorded daily; the total number of nutritional-feeding sessions was not reported<br><br>- Mother's own milk: Mother's milk was quantified as the percentage of total enteral intake; it excluded supplemental donor milk and preterm formula<br><br>- Donor milk: Supplemental donor milk was provided when mother's milk was unavailable in the original trial; one predefined feeding group received ≥20% of feeds as donor milk<br><br>- Preterm formula: Preterm formula was the comparator when mother's milk was unavailable in the original trial; one predefined feeding group received ≥20% of feeds as formula<br><br>- Fortification strategy: Not mentioned; no fortification type, dose, or protocol was reported<br><br>- Quantified exposure: Mean percentage of mother's milk was 72.0% ± 36.8% in the EWAS cohort and 68.2% ± 37.8% in the MRI cohort
**Anthropometric features and growth trajectories**	- Anthropometric features: At the 5.5-year visit, height and weight were measured and BMI was calculated; the follow-up also assessed growth and body composition.<br><br>- Birth and current growth information: Mean birth weight was 1008 ± 264 g, and at a mean age of 5.7 ± 0.2 years the children had a mean BMI z score of −0.27 ± 1.21.<br><br>- Additional baseline anthropometrics: The baseline table includes birth gestational age, birth weight, breastfeeding duration, and length of hospitalization for the EWAS and MRI cohorts.<br><br>- Head circumference: Not mentioned in the supplied paper text.<br><br>- Growth trajectories: Not mentioned; the paper reports growth/body-composition assessment at follow-up but no longitudinal growth trajectory analysis.<br><br>- Growth-adjusted models interpretation: Not mentioned; the supplied analyses do not describe models adjusted for growth trajectories or head circumference.
**MRI results**	- MRI corroboration subset: n = 41 children<br><br>- Cortical surface area: 100% versus 0% mother's milk was associated with 173.1 cm² larger total cortical surface area (95% CI, 73.6 to 272.5; P = 0.002), adjusted for infant sex and maternal education<br><br>- Cortical thickness: not significantly associated with mother's-milk percentage (P = 0.079)<br><br>- DNA methylation and surface area: 6 of the top 20 EWAS CpGs were associated with cortical surface area at P < 0.05; higher mother's-milk exposure was associated with lower DNAm, and lower DNAm with larger cortical surface area<br><br>- CpGs with nominal cortical-surface-area associations: cg07398661,

	cg01756553, cg13313522, cg14033590, and cg08145067 had P values from 0.012 to 0.036
**Key neurodevelopmental outcomes**	- Cortical surface area: Children fed 100% versus 0% mother's milk had a 173.1 cm^2 larger cortical surface area (95% CI, 73.6 to 272.5; P = 0.002), adjusted for sex and maternal education<br><br>- Full-Scale IQ: Children fed 100% versus 0% mother's milk were nearly 6 times more likely to have Full-Scale IQ ≥80 (OR, 5.8; 95% CI, 1.3 to 26.9; P = 0.02)<br><br>- Cortical thickness: The association with mother's milk intake was not statistically significant (P = 0.079)<br><br>- DNA methylation and neurodevelopment: Six top-ranked CpGs were associated with cortical surface area and one with Full-Scale IQ at nominal P < 0.05; higher mother's milk intake was associated with lower methylation, and lower methylation was associated with larger cortical surface area and higher IQ<br><br>- Neurodevelopmental pathways: EWAS-associated genes were enriched for nervous system development, generation of neurons, neurogenesis, neuron differentiation, and regulation of nervous system development<br><br>- Study scope: The EWAS included 143 children at 5.5-year follow-up; corroboration analyses included 41 children with MRI and 133 with Full-Scale IQ data
**Country**	Canada

8. Costa S (2022)

**First author**	Coast S
**Year of publication**	2022
**Nutritional interventions**	- Parenteral lipid intervention: MLE (SMOFlipid 20%) versus SLE (Intralipid 20%)<br><br>- Timing and dosing: started within the first 24 hours of life at 1.5 g/kg/day, increased by 0.5 g/kg/day, and reached 3 g/kg/day within the first week<br><br>- Duration/session frequency: No discrete number of sessions was reported; nutrition was administered daily. PN duration was 50.0 ± 29.5 days with MLE and 44.0 ± 28.6 days with SLE-<br><br>Enteral feeding: minimal enteral feeding started on day 1 of life and increased by 20–30 mL/kg/day<br><br>- Mother's own milk: provided as human milk, with no separate quantity reported<br><br>- Donor milk: provided as an alternative human-milk source, with no separate quantity reported

	- Formula: Not mentioned; the paper states that all infants received mother's own milk or donor milk<br><br>- Fortification: human milk was fortified with Aptamil BMF once enteral intake reached 100 mL/kg/day<br><br>- Monitoring/modification: parenteral lipid intake was reduced by 25–50% at triglycerides of 265–442 mg/dL and stopped above 442 mg/dL
**Anthropometric features and growth trajectories**	- Head circumference: At baseline, HC was 22.0 cm (IQR 21.0–24.1) in the MLE group and 23.1 cm (IQR 21.5–25.3) in the SLE group; HC z-scores were −0.81 ± 0.84 and −0.71 ± 1.06, respectively, with no significant differences. At MRI, HC was 30.5 ± 1.4 versus 29.6 ± 1.8 cm and HC z-score was −1.7 ± 0.94 versus −2.0 ± 0.95, also without significant differences.<br><br>- Growth trajectories: The current paper does not provide longitudinal HC measurements or a modeled HC growth trajectory. The authors cite their previous trial, in which MLE significantly reduced the loss of HC and length z-scores from birth to 36 weeks' PMA or discharge compared with SLE.<br><br>- Growth-adjusted model interpretation: No anthropometric growth-adjusted model is reported. Adjustments in this study apply to MRI brain volumes for PMA and total brain volume; PMA-corrected cerebellar volume was higher with MLE, whereas cerebellar volume corrected for total brain volume was similar between groups.
**MRI results**	- MRI timing: PMA at MRI was 43.5 ± 8 weeks in MLE versus 42.8 ± 10 weeks in SLE; P = 0.8<br><br>- Cerebellar volume: 24.0 ± 9.6 cm³ in MLE versus 18.2 ± 6.2 cm³ in SLE; P = 0.045<br><br>- PMA-corrected cerebellar volume: 22.3 ± 6.5 cm³ in MLE versus 16.9 ± 7.2 cm³ in SLE; P = 0.027<br><br>- Cerebellar volume corrected for total brain volume: 5.1% in both MLE and SLE; P = 0.87<br><br>- Supratentorial volume: 284.7 (253.8–407.9) cm³ in MLE versus 283.4 (233.1–297.8) cm³ in SLE; P = 0.16<br><br>- PMA-corrected supratentorial volume: 358.3 (179.0–396.8) cm³ in MLE versus 294.9 (191.5–345.8) cm³ in SLE; P = 0.25<br><br>- Brainstem volume: 6.0 ± 0.9 cm³ in MLE versus 5.3 ± 9.9 cm³ in SLE; P = 0.053<br><br>- PMA-corrected brainstem volume: 5.7 ± 9.6 cm³ in MLE versus 5.2 ± 1.1 cm³ in SLE; P = 0.11<br><br>- Total brain volume: 379.5 (208.6–419.6) cm³ in MLE versus 316.6 (211.6–364.7) cm³ in SLE; P = 0.21<br><br>- Overall MRI finding: Cerebellar volume and PMA-corrected cerebellar volume

	were significantly higher with MLE, while no significant differences were found for the other brain volumes
**Key neurodevelopmental outcomes**	- TEA neuroimaging: Among 34 infants, 17 received MLE and 17 received SLE.<br><br>- Cerebellar volume: MLE 24.0 ± 9.6 cm^3 vs SLE 18.2 ± 6.2 cm^3; p = 0.045<br><br>- PMA-corrected cerebellar volume: MLE 22.3 ± 6.5 cm^3 vs SLE 16.9 ± 7.2 cm^3; p = 0.027-<br><br>Other MRI brain volumes: No significant differences were found for the other brain volumes, including cerebellar volume corrected for total brain volume.<br><br>- Neurodevelopmental outcome/follow-up: No neurological or neurodevelopmental follow-up results were reported; a 24-month corrected-age follow-up was planned to determine whether the MRI differences corresponded to improved neurodevelopment.
**Country**	Italy

9. Weiming Niu

**First author**	Weiming Niu
**Year of publication**	
**Nutritional interventions**	- Nutritional intervention: Breast milk feeding versus formula feeding; infants with >75% breastfeeding were classified as breast-fed and those with <75% as formula-fed<br><br>- Timing: Daily breast milk and formula intake was recorded from postpartum until the scanning date, around term-equivalent age-<br><br>Number of sessions: Not mentioned (feeding was described by daily volume/percentage, not by intervention sessions).<br><br>- Groups: 30 breast-fed and 20 formula-fed infants; breast-fed percentages were 90.25 ± 8.02% and 28.05 ± 21.60%, respectively<br><br>- Mother's own milk versus donor milk: Not mentioned.<br><br>- Formula composition and fortification strategy: Not mentioned.
**Anthropometric features and growth trajectories**	- Anthropometric features:<br><br>- Weight at birth: breast-fed [1845.89 ± 351.42 g]; formula-fed [1807.90 ± 382.20 g]; between-group p = [0.661].<br><br>- Height at birth: breast-fed [42.71 ± 2.51 cm]; formula-fed [41.88 ± 2.87 cm]; between-group p = [0.206].<br><br>- Head circumference at birth: breast-fed [30.29 ± 1.61 cm]; formula-fed

	[29.83 ± 1.73 cm]; between-group p = [0.351].<br><br>- Weight at MRI: breast-fed [2408.39 ± 345.27 g]; formula-fed [2399.50 ± 381.40 g]; between-group p = [0.916].<br><br>- Height at MRI: breast-fed [45.36 ± 2.61 cm]; formula-fed [45.60 ± 2.64 cm]; between-group p = [0.843].<br><br>- Head circumference at MRI: breast-fed [32.07 ± 1.18 cm]; formula-fed [32.23 ± 1.11 cm]; between-group p = [0.604].<br><br>- Growth trajectories: Not mentioned; the paper reports measurements at birth and MRI rather than longitudinal trajectories.<br><br>- Growth-adjusted models interpretation: Not mentioned; the authors state that HCF and body weight showed no significant between-group differences at either birth or scanning.
**MRI results**	- MRI modality and sample: Resting-state fMRI was acquired at term-equivalent age in 50 preterm infants; 30 were breast-fed and 20 formula-fed, scanned at approximately 40 weeks PMA on a 1.5-T scanner<br><br>- Network organization: Both groups exhibited temporal small-world architecture<br><br>- Global functional-network result: Breast-fed infants had significantly higher temporal global efficiency than formula-fed infants (t_{48} = 2.418, p = 0.019, Cohen's d = 0.705)<br><br>- Local functional-network result: Temporal local efficiency did not differ significantly between groups (t_{48} = −0.597, p = 0.553)<br><br>- Regional findings: Breastfeeding was associated with higher temporal regional efficiency in the right middle temporal gyrus, right inferior temporal gyrus, and bilateral caudate nuclei<br><br>- Dose-related association: Temporal global efficiency correlated positively with breastfeeding percentage (r = 0.362, p = 0.014)<br><br>- Volumetrics/morphometrics: Not mentioned as outcomes; the reported MRI results concern dynamic functional connectivity and temporal network efficiency
**Key neurodevelopmental outcomes**	- TEA: MRI was acquired at term-equivalent age, approximately 40 weeks PMA (range 39–44 weeks)<br><br>- Neurodevelopment outcome: Breastfed infants had higher temporal global efficiency than formula-fed infants (t_{48} = 2.418; P = .019; Cohen's d = 0.705)<br><br>- Regional findings: Breastfeeding was associated with higher temporal regional efficiency in the right middle temporal gyrus, right inferior temporal gyrus, and bilateral caudate nuclei<br><br>- Dose association: Breastfeeding percentage was positively correlated with temporal global efficiency (r = 0.362; P = .014)

	- Follow-up: Not mentioned (the provided paper reports term-equivalent-age neuroimaging outcomes, not later neurodevelopmental follow-up).
**Country**	China

10. Katherine M Ottolini (2025)

**First author**	Katherine M Ottolini
**Year of publication**	2025
**Nutritional interventions**	- Intervention design: Observational nutritional exposure, categorized as mother's own milk (MOM), donor human milk (DHM), or preterm formula (PTF) when the feed type comprised >50% of cumulative enteral volume<br><br>- Participants: 67 MOM, 44 DHM, and 41 PTF infants<br><br>- Timing and duration: Parenteral nutrition began on NICU admission; trophic enteral feeds began within 24 hours of life or after clinical stabilization, and daily enteral volumes were recorded from admission until discharge or term-equivalent-age MRI, whichever occurred first<br><br>- Mother's own milk: Maternal, unpasteurized milk was one of the three primary enteral nutrition categories; the MOM group had a median 100% maternal-milk intake<br><br>- Donor milk: Pasteurized donor human milk was provided with parental consent when maternal supply was insufficient, beginning in June 2014, until approximately 34 weeks corrected age, followed by gradual transition to preterm formula; the DHM group had a median 67% donor-milk intake<br><br>- Formula: Preterm formula was the comparator enteral nutrition category; the PTF group had a median 91% formula intake<br><br>- Fortification: Both maternal and donor milk were fortified with a liquid bovine-based human milk fortifier to 22 kcal/oz at 80 ml/kg/day and 24 kcal/oz at 100 ml/kg/day<br><br>- Additional nutritional support: For growth faltering, caloric density was increased with additional formula supplementation to >24 kcal/oz or modular liquid protein/MCT oil was added<br><br>- Number of sessions: Not mentioned; the study reports daily feeding volumes and cumulative exposure rather than a discrete number of nutritional intervention sessions
**Anthropometric features and growth trajectories**	- Measurements: Weekly weight, length, and head circumference were extracted from medical records; growth faltering was defined as a decrease in weight z-score of >1 SD from birth to term-equivalent age.<br><br>- Birth weight: 976 ± 326 g overall; MOM 1033 ± 324 g, DHM 899 ± 303 g, and PTF 966 ± 340 g.<br><br>- Birth-weight z-score: MOM −0.24 ± 0.96, DHM −0.27 ± 0.80, and PTF −0.33

	± 0.97.<br><br>- Growth faltering: MOM 40/67 (60%), DHM 32/44 (73%), and PTF 22/41 (54%); the between-group comparison was not significant (P = 0.17).<br><br>- Growth trajectories: Not mentioned (weekly measures were collected, but longitudinal trajectories for weight, length, or head circumference were not reported).<br><br>- Growth-adjusted models: Not mentioned as anthropometric growth-adjusted models; MRI models adjusted for gestational age at birth and postmenstrual age at MRI, while additional models incorporated infant sex and SVI.
**MRI results**	- MRI cohort: 152 very preterm infants underwent volumetric MRI at term-equivalent age: 67 mother's own milk, 44 donor human milk, and 41 preterm formula<br><br>- Volumetrics:<br><br>- Donor human milk vs mother's own milk: No significant differences in adjusted brain volumes, including in the >70% donor-milk sensitivity analysis<br><br>- Preterm formula vs mother's own milk: Lower total brain volume (β = −17.2; p = 0.016), deep gray matter (β = −1.2; p = 0.024), and brainstem (β = −0.4; p = 0.002); cortical gray matter and white matter were also lower, but p values were 0.028 and 0.026, respectively<br><br>- Preterm formula vs donor human milk: No significant difference in brain volumes<br><br>- White-matter maturation by DTI:<br><br>- Donor human milk vs mother's own milk: Lower mean diffusivity in the right pons (β = −0.04; p = 0.008) and left pons (β = −0.06; p < 0.001), with no significant fractional-anisotropy differences<br><br>- Preterm formula vs mother's own milk: Higher mean diffusivity in the corpus-callosum genu (β = 0.11; p = 0.007), splenium (β = 0.13; p = 0.007), and bilateral PLIC (right β = 0.06; p = 0.002; left β = 0.06; p = 0.001), plus lower right PLIC fractional anisotropy (β = −0.02; p = 0.019)<br><br>- Overall MRI finding: Donor human milk showed brain volumes comparable to mother's own milk, whereas preterm formula was associated with smaller regional and total brain volumes and greater white-matter microstructural alterations
**Key neurodevelopmental outcomes**	- TEA neurodevelopmental/imaging cohort: 152 very preterm infants: 67 mother's-own-milk, 44 donor-human-milk, and 41 preterm-formula infants; outcomes were assessed with term-equivalent MRI-<br><br>Brain volumes: Donor human milk did not differ significantly from mother's own milk in total or regional brain volumes; preterm formula was associated with lower total brain volume (β = −17.2; P = .016), deep gray matter volume (β = −1.2; P = .024), and brainstem volume (β = −0.4; P = .002) versus mother's own milk<br><br>- White-matter microstructure: Donor human milk showed lower mean

	diffusivity in the right and left pons versus mother's own milk (β = −0.04, P = .008; β = −0.06, P < .001), with no significant FA differences; preterm formula showed higher mean diffusivity in the corpus callosum and posterior limb of the internal capsule and lower FA in the right posterior limb of the internal capsule<br><br>- Follow-up: Long-term neurodevelopmental follow-up was underway; no completed post-discharge neurodevelopmental outcomes were reported
**Country**	United States

11. Dennis Savaiano (2026)

**First author**	Dennis Savaiano
**Year of publication**	2026
**Nutritional interventions**	- Intervention: Prolonged exclusive human milk-based diet (EHMD) with continued human milk-based fortification until 36 weeks postmenstrual age<br><br>- Comparator: Earlier introduction of bovine milk-based fortifier or formula from 32 weeks postmenstrual age<br><br>- Mother's own milk: Used as part of the human-milk feeding strategy; the paper describes mothers' own milk as the preferred milk source but does not report an arm-specific dose or volume<br><br>- Donor human milk: Could form part of the base diet, but arm-specific exposure was not reported<br><br>- Formula: Permitted/introduced as a comparator component from 32 weeks postmenstrual age; only 42% of control infants were exposed to bovine milk before 32 weeks<br><br>- Fortification strategy: Human milk-based fortification was prolonged in the intervention group; the nutritional contrast primarily concerned the duration of exclusive human milk-based fortification rather than the initial enteral milk type<br><br>- Timing: Intervention continued until 36 weeks postmenstrual age; comparator exposure to bovine milk-based products began at 32 weeks postmenstrual age<br><br>- Number of nutritional sessions: Not mentioned; nutrition was described as an ongoing feeding strategy rather than a session-based intervention<br><br>- Assessment timing, not intervention sessions: GMOS assessments occurred at 34, 36, and 40 weeks postmenstrual age, and MOS at 52 weeks
**Anthropometric features and growth trajectories**	- Head circumference at birth: 26.0 cm (IQR 24.5–26.9) in the intervention group vs 26.5 cm (IQR 25.5–27.0) in controls; p = 0.426<br><br>- Growth status: Small-for-gestational-age infants were more frequent in the intervention group, 9/28 (32.1%), than in controls, 3/26 (11.5%), but the difference was not statistically significant (p = 0.069)

	- Growth trajectories: Not mentioned; the paper does not report serial weight, length, or head-circumference trajectories<br><br>- Quantitative brain growth metrics: No significant between-group differences were observed<br><br>- Growth-adjusted model interpretation: [After adjustment for small-for-gestational-age status, the right posterior limb of the internal capsule FA difference was no longer significant (adjusted $R^2 = 0.106$; $F[2.48] = 3.979$; $p = 0.101$), suggesting that the unadjusted finding may have reflected baseline growth differences rather than a true nutritional effect]
**MRI results**	- Brain injury: No significant between-group differences in white matter injury, cerebellar hemorrhage, or intraventricular hemorrhage prevalence or severity; MRI injury and quantitative brain-growth results are detailed in the table<br><br>- Volumetric/morphometric measures: No significant differences in biparietal width, interhemispheric distance, parietal extracerebral space, transcerebellar diameter, temporal lobe length, or temporal extracerebral space<br><br>- Fractional anisotropy: Unadjusted FA was lower in the right posterior limb of the internal capsule in the intervention group: 0.59 (IQR 0.56–0.61) versus 0.62 (IQR 0.60–0.64), P = 0.019; this was not significant after adjustment for SGA<br><br>- Apparent diffusion coefficient: Unadjusted ADC was lower in the left middle cerebellar peduncle in the intervention group: 1.00 (IQR 0.97–1.07) versus 1.05 (IQR 1.00–1.08), P = 0.027; this was not significant after adjustment for maternal smoking<br><br>- Overall MRI interpretation: MRI findings did not show consistent differences in early structural brain maturation between prolonged-EHMD and control groups
**Key neurodevelopmental outcomes**	- Structural brain maturation at term-equivalent age: No significant differences in brain injury prevalence/severity or quantitative MRI brain-growth metrics.<br><br>- Diffusion metrics at term-equivalent age: Unadjusted FA and ADC differences were no longer statistically significant after adjustment.<br><br>- GMOS at 40 weeks postmenstrual age: Higher with prolonged EHMD: median 33.0 vs 28.5; p = 0.011; adjusted p = 0.037.<br><br>- GMOS at 34 and 36 weeks postmenstrual age: No statistically significant differences: p = 0.271 and p = 0.059, respectively.<br><br>- MOS at 52 weeks postmenstrual age: No difference: median 26.0 vs 26.0; p = 0.732.<br><br>- Overall interpretation: The isolated GMOS difference at 40 weeks was not accompanied by consistent differences in brain maturation or motor performance and was unlikely to be clinically meaningful.
**Country**	Austria

12. Suvi M Manninen (2022)

**First author**	Suvi M Manninen
**Year of publication**	2022
**Nutritional interventions**	- Intervention: No assigned nutritional intervention was reported; this was a prospective cohort study in which daily nutrient intakes were registered.<br><br>- Timing and number of sessions: Nutrient intake was recorded at postnatal week 1 and week 5 (2 assessment time points; intervention sessions were not reported).<br><br>- Mother's own milk: Not mentioned.<br><br>- Donor milk: Not mentioned.<br><br>- Formula: Not mentioned.<br><br>- Fortification strategy: Not mentioned.<br><br>- Carnitine source: Enteral milk at weeks 1 and 5.
**Anthropometric features and growth trajectories**	- Head circumference: Carnitine intake and serum carnitine levels at W1, W5, and TEA were positively correlated with head-circumference Z-score change from birth to TEA<br><br>- Growth trajectories: Reported as weight, length, and head-circumference Z-score change from birth to term-equivalent age; detailed longitudinal trajectories are not provided in the abstract<br><br>- Growth-adjusted models: Linear models showed independent positive associations of carnitine intake and serum carnitine levels with length and head-circumference Z-score change; no independent weight association is stated
**MRI results**	- MRI brain size at TEA: Carnitine intake and serum carnitine levels at W1, W5, and TEA were positively associated with brain size at TEA<br><br>- Adjusted analysis: Independent positive associations of carnitine intake and serum carnitine levels with brain size at TEA were observed in linear models<br><br>- Specific volumetric, morphometric, or maturation results: Not mentioned (the abstract reports only brain size at TEA).
**Key neurodevelopmental outcomes**	- Brain size at TEA: Carnitine intake and serum carnitine levels at W1, W5, and TEA were positively associated with brain size at TEA; these associations remained independent in linear models<br><br>- Neurodevelopmental follow-up: Not mentioned; the abstract reports MRI brain size at TEA but no later neurodevelopmental assessment or follow-up outcome
**Country**	Finland

13. Mountasser M Al-Mouqdad

**First author**	Mountasser M Al-Mouqdad
**Year of publication**	
**Nutritional interventions**	- Parenteral lipid intervention: SMOFlipid (soybean oil 30%, medium-chain triglycerides 30%, olive oil 25%, fish oil 15%) or Intralipid 20% (soybean oil and MCT)<br><br>- Timing: TPN and lipid emulsion were initiated within the first 24 hours of life; enteral feeding began as soon as possible after birth<br><br>- Number of sessions/doses: One individualized TPN bag was infused over 24 hours and replaced daily; lipid dose was increased every 24 hours from 0.5–1.0 g/kg/day to a maximum of 3.0–3.5 g/kg/day<br><br>- Enteral milk: Expressed breast milk was used via orogastric tube; preterm formula was used when human milk was unavailable or insufficient<br><br>- Donor milk: Not mentioned<br><br>- Fortification: Not mentioned; no breast-milk or formula fortification strategy was reported<br><br>- TPN discontinuation: TPN was stopped when enteral intake reached 100 mL/kg/day<br><br>- Intervention groups: 64 infants received Intralipid and 86 received SMOFlipid
**Anthropometric features and growth trajectories**	- Measurement approach: Body weight was recorded daily and head circumference weekly; Fenton 2013 charts were used for growth Z-scores.<br><br>- Discharge weight: 2022 g (IQR 1847–2250) with Intralipid vs 2305 g (1925–2880) with SMOFlipid; P = 0.12<br><br>- Weight Z-score at discharge: −2.61 (−5.01 to −1.52) vs −2.15 (−3.03 to −1.43); P = 0.33<br><br>- Length at discharge: 41.5 cm (39–45) vs 45 cm (41–48); P = 0.09<br><br>- Length Z-score at discharge: −4.49 (−5.96 to −1.49) vs −2.93 (−4.15 to −0.66); P = 0.20<br><br>- Head circumference at discharge: 30.5 cm (27.25–32.5) with Intralipid vs 32 cm (30.5–34.5) with SMOFlipid; P = 0.05<br><br>- Head-circumference Z-score at discharge: −2.59 (−6.78 to −1.16) vs −1.08 (−2.97 to −0.39); P = 0.08<br><br>- Weight-growth trajectory: Change in weight Z-score was −1.20 (−2.54 to −0.76) with Intralipid vs −2.19 (−3.10 to −1.50) with SMOFlipid; P = 0.03<br><br>- Growth-adjusted models: No adjusted effect estimate for weight, length, or

	growth trajectory was reported; after adjustment, the authors stated that lipid type did not affect head circumference.
**MRI results**	- Sample: 148 infants underwent term-equivalent-age MRI: 84 received SMOFlipid and 64 received Intralipid<br><br>- White-matter focal signal abnormality: higher with SMOFlipid; adjusted coefficient 1.73 (95% CI 1.10–2.72), P = 0.02<br><br>- White-matter myelination delay: greater with Intralipid than SMOFlipid; adjusted coefficient for SMOFlipid 0.76 (95% CI 0.63–0.93), P = 0.007<br><br>- Cortical gray-matter extracerebral space: increased with SMOFlipid; adjusted coefficient 2.97 (95% CI 1.26–7.02), P = 0.01<br><br>- Cerebellar volume reduction: higher with SMOFlipid; adjusted coefficient 1.22 (95% CI 1.03–1.44), P = 0.02<br><br>- Other<br><br>MRI findings: no significant adjusted differences were reported for cystic lesions, corpus-callosum thinning, ventricular dilatation, white-matter volume reduction, cortical signal abnormality, gyral maturation, deep-gray-matter abnormalities, cerebellar signal abnormality, or total cerebellar score
**Key neurodevelopmental outcomes**	- TEA MRI/neurodevelopmental assessment: 148 very preterm infants were assessed at term-equivalent age using brain MRI and the Kidokoro brain-growth/morphology score<br><br>- Focal white-matter signal abnormality: higher with SMOFlipid than Intralipid after adjustment; adjusted coefficient 1.73 (95% CI 1.10–2.72), P = 0.02<br><br>- White-matter myelination delay: associated with lipid-emulsion group differences after adjustment; adjusted coefficient 0.76 (95% CI 0.63–0.93), P = 0.007<br><br>- Increased cortical extracerebral space: higher with SMOFlipid; adjusted coefficient 2.97 (95% CI 1.26–7.02), P = 0.01<br><br>- Cerebellar volume reduction: higher with SMOFlipid; adjusted coefficient 1.22 (95% CI 1.03–1.44), P = 0.02<br><br>- Global brain abnormality score: not significantly different after adjustment; adjusted coefficient 1.21 (95% CI 0.98–1.50), P = 0.09<br><br>- Later neurodevelopmental follow-up: not reported; the authors state that longitudinal follow-up across childhood is needed
**Country**	Saudi Arabia

14. Yao Zhang (2022)

**First author**	Yao Zhang

**Year of publication**	2022
**Nutritional interventions**	- Mother's own milk: Gavage-fed breast milk for a minimum of 90 days; group allocation was based on whether mothers had adequate breast milk<br><br>- Formula: The formula-fed group received a mixture of breast milk and formula, with formula volume comprising 80% more than breast milk<br><br>- Timing: Feeding was assessed during the first year of life, with breastmilk feeding specified for a minimum of 90 days<br><br>- Number of sessions: Not mentioned<br><br>- Donor milk: Not mentioned<br><br>- Fortification strategy: Not mentioned
**Anthropometric features and growth trajectories**	- Birth weight: breastmilk-fed 1839.8 ± 345.9 g vs formula-fed 1800.1 ± 364.7 g; P = 0.683<br><br>- Weight after discharge: breastmilk-fed 2367.7 ± 350.6 g vs formula-fed 2437.8 ± 427.9 g; P = 0.493<br><br>- Head circumference at birth: breastmilk-fed 30.27 ± 1.65 cm vs formula-fed 29.80 ± 1.72 cm; P = 0.287<br><br>- Head circumference after discharge: breastmilk-fed 31.88 ± 1.33 cm vs formula-fed 32.47 ± 1.26 cm; P = 0.086<br><br>- Growth trajectories: [Not reported; the study provides measurements at birth and after discharge but no longitudinal trajectory analysis]<br><br>- Growth-adjusted models: [Weight and head circumference were analyzed using ANCOVA, and weight after discharge and head circumference after discharge were considered potential confounders; their effects on the MRI–feeding relationship were not identified]
**MRI results**	- Gray-matter volume: Breastmilk-fed infants had greater regional gray-matter volume than formula-fed infants in the bilateral frontal lobes (BA11, BA46), left caudate nucleus, and right temporal lobe (BA37); no regions had lower gray-matter volume (p < 0.01, AlphaSim-corrected; cluster size >40 voxels)<br><br>- Functional MRI: Breastmilk-fed infants showed greater activation, including in the right superior temporal gyrus/right temporal lobe, with a significantly larger activation cluster than formula-fed infants-<br><br>Overall MRI finding: Around term-equivalent age, breastmilk-fed infants showed greater regional gray-matter volume and greater right temporal-lobe brain activation than formula-fed infants
**Key neurodevelopmental outcomes**	- TEA neuroimaging: At approximately term-equivalent age, breastmilk-fed infants had greater gray matter volume in the bilateral frontal lobe, left caudate nucleus, and right temporal lobe, with no regions of lower gray matter volume<br><br>- Functional result: Breastmilk-fed infants had a higher BOLD response in the right superior temporal gyrus and were described as having potentially better

	early functional development<br><br>- Neurodevelopmental outcome/follow-up: No direct neurodevelopmental outcome or completed follow-up result was reported; the study only investigated the short-term impact, and early-childhood follow-up was proposed for future studies
**Country**	China

15. Ottolini KM (2020)

**First author**	Ottolini KM
**Year of publication**	2020
**Nutritional interventions**	- Intervention scope: Ongoing enteral nutritional feeding during the NICU admission, classified as primarily breast milk or formula when the feed type constituted >50% of total enteral volume for the admission.<br><br>- Timing: Trophic enteral feeds were initiated within 24 hours of life or once clinically stable; daily intake was recorded retrospectively from admission until the term-equivalent MRI.<br><br>- Maternal own milk: 30 infants received primarily maternal breast milk; most breast-milk-fed infants had exclusive breast milk throughout admission, with mean intake 86% of total enteral feeds and median 90%.<br><br>- Donor milk: 14 infants received primarily donor breast milk; pasteurised donor milk was used when maternal supply was insufficient, with parental consent, until 34 weeks corrected age.<br><br>- Formula: 24 infants received primarily preterm formula; donor-milk infants were transitioned to formula at 34 weeks corrected age and had mean breast-milk exposure of 69% (median 64%) during the NICU admission.<br><br>- Fortification: Maternal and donor breast milk were fortified identically with a cow's-milk-based human milk fortifier.<br><br>- Number of sessions: Not mentioned; the paper describes continuous NICU enteral feeding and daily nutritional-intake recording rather than a reported number of intervention sessions.
**Anthropometric features and growth trajectories**	- Birth head circumference: 24.3 cm (IQR 21.8–26.4) in the breast-milk group vs 24.3 cm (IQR 23.0–27.0) in the formula group; P = .50<br><br>- Other baseline anthropometrics: Median gestational age was 26.9 vs 28.0 weeks, median birth weight was 0.90 vs 0.96 kg, and small-for-gestational-age status was 9.1% vs 12.5% in the breast-milk and formula groups, respectively-<br><br>Growth trajectories: Not mentioned; the paper reports baseline head circumference but no serial head-circumference measurements or head-circumference growth trajectories<br><br>- Growth-adjusted models: Brain volumes were compared using models

	adjusted for gestational age at birth, postmenstrual age at MRI, and average weight gain; total brain volume, amygdala-hippocampus, cerebellum, and brainstem volumes remained significantly larger in the breast-milk group<br><br>- Interpretation of additional adjustment: After adjustment for total brain volume, only amygdala-hippocampus and cerebellar volumes remained significantly larger in the breast-milk group
**MRI results**	- Volumetric MRI (67 infants; 43 breast milk, 24 formula): breast milk-fed infants had larger total brain volume (322 vs 305 cm^3; P = .04), amygdala-hippocampus volume (2.3 vs 2.1 cm^3; P < .01), cerebellar volume (22 vs 19 cm^3; P < .01), and brainstem volume (5.3 vs 5.0 cm^3; P = .02) than formula-fed infants. The amygdala-hippocampus and cerebellum remained significantly larger after adjustment for total brain volume. Adjusted brain volumes were reported from least-squares models accounting for gestational age, postmenstrual age at MRI, and average weight gain<br><br>- DTI/microstructural MRI (65 infants; 42 breast milk, 23 formula): breast milk-fed infants had lower mean diffusivity in the corpus callosum (1.28 vs 1.38 × 10^{-3} mm^2/s; P = .03), left PLIC (0.92 vs 0.99; P < .01), right PLIC (0.94 vs 0.98; P = .03), and middle cerebellar peduncle (0.80 vs 0.86; P = .02), plus lower fractional anisotropy in the cerebellar vermis (0.19 vs 0.23; P < .01). These findings were interpreted as greater white-matter microstructural organisation/maturation, although corpus-callosum and right-PLIC mean-diffusivity differences were no longer significant after infection adjustment<br><br>- Overall MRI result: early breast milk feeding was associated with greater cerebral/cerebellar growth and regional differences in white-matter microstructural organisation by term-equivalent age. The study's volumetric and DTI findings support this conclusion
**Key neurodevelopmental outcomes**	- TEA assessment:<br><br>Neurodevelopmental outcome at TEA: Compared with formula feeding, primarily breast milk feeding was associated with larger total brain, amygdala-hippocampus, cerebellar and brainstem volumes, and lower mean diffusivity in the corpus callosum, bilateral PLIC and middle cerebellar peduncle, with lower vermis fractional anisotropy<br><br>- Interpretation: Findings indicated improved early brain growth and white-matter microstructural development by term-corrected age; similar benefits were observed in secondary analyses of donor breast milk<br><br>- Follow-up: No long-term neurodevelopmental outcomes were reported; developmental follow-up was ongoing and required to determine the functional significance of the MRI findings
**Country**	United States of America

16. Rebecca D Henkel

**First author**	Rebecca D Henkel

**Year of publication**	
**Nutritional interventions**	- Intervention type: Observational nutritional exposure—not a scheduled intervention with discrete sessions—comparing the relative proportion of enteral versus parenteral protein received during the first 28 days after birth<br><br>- Timing and duration: Enteral feeds started within 48 hours of birth; infants typically reached full enteral feeding volume by 10–14 days; parenteral protein started on the day of birth; mean TPN exposure was 14.1 days (SD 5.2), with 76% receiving TPN for less than 2 weeks<br><br>- Number of sessions: Not mentioned; nutrition was recorded as daily intake over the first 28 days rather than as discrete treatment sessions<br><br>- Mother's own milk: Included as a source of enteral feeding, together with donor breast milk; the paper does not report a separate percentage for maternal milk alone<br><br>- Donor milk: Included as a source of enteral feeding; the paper does not report a separate percentage for donor milk alone<br><br>- Formula: Among infants with any formula exposure, 66% received formula only on day 26 or later<br><br>- Fortification strategy: Feeds advanced to 160 mL/kg/day, with a holding day at 75 mL/kg/day to fortify to 24 kcal/ounce; fortification products and addition of liquid protein fortifier to donor milk varied between centers<br><br>- Reported nutritional exposure: Mean E:P ratio 2.72 (SD 1.31), total protein intake 4.01 (0.42) g/kg/day, enteral protein intake 2.60 (0.88) g/kg/day, total energy intake 106.38 (11.95) kcal/kg/day, and enteral energy intake 76.61 (23.35) kcal/kg/day
**Anthropometric features and growth trajectories**	- Anthropometric assessment: Weight, length, and head circumference were recorded at birth and 28 days; z scores used Fenton growth curves, and weight-gain velocity was calculated by the exponential method.<br><br>- Mean growth trajectory over the first 28 days: weight z score −0.53 (SD 0.53); length z score −0.84 (SD 0.66); head-circumference z score −0.87 (SD 0.90); weight-gain velocity 14.08 (SD 3.93) g/kg/d.<br><br>- Growth-adjusted model: Models adjusted for CRIB-II score, hypertensive disorders of pregnancy, smoking in pregnancy, birth hospital, and total protein intake; growth z scores were based on Fenton charts.<br><br>- E:P ratio: Associated with increased weight-gain velocity, β = 0.74 (95% CI 0.24 to 1.24; P < .01), but not with change in weight z score, length z score, or head-circumference z score.<br><br>- Enteral protein: Associated with increased length z score, β = 0.13 (95% CI 0.03 to 0.24; P = .01).<br><br>- Head circumference interpretation: No significant E:P-ratio association was found for head-circumference z-score change; the negative total-protein association was unexpected and may reflect retrospective, nonstandardized measurement variability.

**MRI results**	- Total brain tissue volume: Higher E:P ratio was associated with greater volume (β = 5.61 mm³; 95% CI, 0.13 to 11.09; P = .05) in the multivariable model<br><br>- Total brain tissue volume with enteral protein exposure: β = 7.58 mm³ per g/kg/d; 95% CI, 1.30 to 13.86; P = .02<br><br>- Regional brain volumes: No significant associations with E:P ratio were reported<br><br>- MRI timing/measurement: Brain MRI was obtained at term-equivalent age, and the primary outcome was total brain tissue volume
**Key neurodevelopmental outcomes**	- At TEA, higher E:P ratio was associated with greater total brain tissue volume on MRI (β = 5.61 mm³; 95% CI, 0.13 to 11.09; P = .05) tea_brain_volume<br><br>- Enteral protein intake was associated with greater total brain tissue volume at TEA (β = 7.58 mm³ per g/kg/d; 95% CI, 1.30 to 13.86; P = .02) enteral_brain_volume<br><br>- Regional brain volumes: no significant associations with E:P ratio regional_brain_results<br><br>- Neurodevelopmental outcome follow-up: Not mentioned (2-year corrected-age neurodevelopmental outcomes were planned for future analyses) future_neurodevelopment_followup
**Country**	United States

17. Bell K.A. (2022)

**First author**	Bell K.A.
**Year of publication**	2022
**Nutritional interventions**	- Intervention design: No assigned nutritional intervention; this was a prospective observational study of routine NICU nutrition<br><br>- Parenteral nutrition: Provided to all infants with birthweight <1800 g<br><br>- Enteral feeding: Advanced to a target of 150–160 mL/kg/day<br><br>- Mother's own milk: Primary enteral milk source; 46/50 infants received exclusively maternal milk at 28 days, 2 received ≥75% maternal milk, and 2 received ≥50% donor milk<br><br>- Donor milk: Used in 2 infants at ≥50% of feeds at 28 days; these infants had received exclusively human milk through 21 days<br><br>- Formula: Not mentioned as a specific component of the feeding regimen; the reported cohort was predominantly or exclusively human-milk fed<br><br>- Fortification: Standard fortification with a multicomponent bovine-based

	human milk fortifier; liquid protein and/or medium-chain triglycerides were added when growth faltering indicated them<br><br>- Timing of milk analysis/intervention monitoring: 5 mL samples of unfortified maternal milk were collected once daily on weekdays from attainment of 100 mL/kg/day enteral feeds through 36 weeks' postmenstrual age<br><br>- Number of sessions/samples: Mean 38.7 samples per infant (SD 17.1; range 14–84)
**Anthropometric features and growth trajectories**	- Head circumference: Not mentioned; the anthropometric table reports gestational age, birth weight, birthweight Z-score, and weight Z-score at term-equivalent age, but no head-circumference measure.<br><br>- Growth trajectories: Daily weight was abstracted, but no longitudinal weight-growth trajectory or trajectory analysis was reported; the study reports weight Z-score at term-equivalent age.<br><br>- Growth-adjusted models interpretation: Brain-volume estimates compare infants in the top nutrient-intake quintile with those in the lower four quintiles, adjusted for gestational age, sex, birthweight Z-score, postmenstrual age at MRI, prematurity comorbidities, and twin relatedness.
**MRI results**	- Volumetrics at term-equivalent age: Compared with the lower four intake quintiles, the highest energy-intake quintile was associated with larger total brain volume by 30.9 cc (95% CI, 5.5 to 56.4), cortical gray matter by 15.3 cc (95% CI, 0.8 to 29.9), and white matter by 22.9 cc (95% CI, 12.2 to 33.4); higher protein intake was associated with larger total brain volume by 36.0 cc (95% CI, 7.1 to 64.8), cortical gray matter by 22.2 cc (95% CI, 6.7 to 37.8), and deep gray matter by 1.5 cc (95% CI, 0.1 to 2.9)<br><br>- Other volumetric regions: Fat and carbohydrate intake were not significantly associated with brain volumes, and energy intake was not significantly associated with deep gray matter, hippocampal, or cerebellar volume<br><br>- White-matter maturation: No statistically significant associations were found between protein, fat, carbohydrate, or energy intake and fractional anisotropy in any of the 15 assessed white-matter tracts; effect estimates were generally positive, but all confidence intervals included the null<br><br>- MRI sample and technique: MRI was obtained at term-equivalent age using a 3-Tesla scanner; automated segmentation quantified total, cortical gray-matter, deep gray-matter, white-matter, hippocampal, and cerebellar volumes, and eight infants lacked volumetric data while one lacked diffusion data
**Key neurodevelopmental outcomes**	- TEA assessment: Brain MRI quantified total/regional brain volumes and fractional anisotropy of white-matter tracts at term-equivalent age<br><br>- Brain volumes: Higher energy intake was associated with larger total brain volume (+30.9 cc), cortical gray matter (+15.3 cc), and white matter (+22.9 cc); higher protein intake was associated with larger total brain volume (+36.0 cc), cortical gray matter (+22.2 cc), and deep gray matter (+1.5 cc), comparing the top with the lower four intake quintiles<br><br>- White-matter microstructure: No fractional-anisotropy association reached statistical significance; confidence intervals did not exclude the null

	- Neurodevelopmental outcome/follow-up: Direct neurodevelopmental testing and later follow-up were not available; TEA brain volume was noted as an independent predictor of cognitive outcomes in early childhood and school age
**Country**	Not mentioned (the supplied paper does not specify the country).

18. Naseh N (2025)

**First author**	Naseh N
**Year of publication**	2025
**Nutritional interventions**	- Type and timing: Standardized protocol beginning within 2 hours after birth, with enteral feeds every 2 hours and remaining nutritional requirements provided parenterally-<br><br>Enteral advancement: Daily increases of 20–40 mL/kg/day toward 150–170 mL/kg/day within 1–2 weeks, depending on gestational age and enteral tolerance<br><br>- Mother's own milk: Used as one of the exclusive human-milk sources from initiation of feeds<br><br>- Donor milk: Used as the alternative exclusive human-milk source from initiation of feeds<br><br>- Formula: Not used or reported; the protocol states that infants exclusively received mother's own or donor human milk<br><br>- Fortification: Initiated at postnatal day 4 or after removal of the umbilical artery catheter<br><br>- Fortifier and targets: PreNAN FM 85; 2.4 g protein/100 mL until 28 weeks PMA, then 2.1 g/100 mL thereafter<br><br>- Monitoring: Maternal milk was analyzed every 2 weeks, and each donor-milk batch was tested and fortified accordingly<br><br>- Number of sessions: Not specified as a total number of sessions; feeds were administered every 2 hours
**Anthropometric features and growth trajectories**	- Head circumference: Not mentioned (no head-circumference measurements or trajectories are reported).<br><br>- Birth anthropometrics:<br><br>- Birth weight: 1190 ± 401 g overall; 1401 ± 328 g in very preterm infants; 824 ± 201 g in extremely preterm infants<br><br>- Birth-weight z-score: −0.98 ± 1.2 overall; −1.29 ± 1.4 in very preterm infants; −0.44 ± 0.7 in extremely preterm infants-<br><br>Early growth trajectory:

	- Maximum weight loss: 9 ± 5% overall; 8 ± 3% in very preterm infants; 12 ± 7% in extremely preterm infants<br><br>- Exceeded birth weight by day 7: 28% overall; 20% of very preterm infants; 42% of extremely preterm infants<br><br>- Exceeded birth weight by day 14: 78% overall; 72% of very preterm infants; 88% of extremely preterm infants<br><br>- Growth metric: body-weight changes were calculated as z-scores and percentages<br><br>- Growth-adjusted interpretation: Additional regression analyses found no associations between nutrient intakes or growth and brain volumes, brain morphology, or neurodevelopment.
**MRI results**	- MRI at term-equivalent age: performed at 40.0 ± 1.7 weeks PMA using T2-, T2*-, volumetric T1-weighted, and diffusion-weighted sequences; ADC was measured in periventricular white matter, basal ganglia, and pons<br><br>- MRI cohort: 69 infants had MRI data; 49 (71%) had no IVH, 23 (33%) had grade 1–2 IVH, none had grade 3–4 IVH, and 9 (13%) had white matter injury<br><br>- Volumetry:<br><br>- Total brain volume: 377 ± 45 mL overall; 382 ± 47 mL in VPT infants and 368 ± 41 mL in EPT infants<br><br>- Cerebellar volume: 26 ± 4 mL overall; 26 ± 3 mL in VPT infants and 26 ± 5 mL in EPT infants<br><br>- White matter volume: 113 ± 23 mL overall; 121 ± 22 mL in VPT infants versus 101 ± 20 mL in EPT infants, the reported significant subgroup difference<br><br>- Diffusion/maturation measures: ADC was 1423 ± 159 × 10^{-6} mm^2/s in white matter, 987 ± 132 × 10^{-6} mm^2/s in the pons, and 1077 ± 62 × 10^{-6} mm^2/s in the basal ganglia overall<br><br>- Morphometric comparison: no significant differences in brain volumes or Kidokoro scores were observed between EPT and VPT subgroups<br><br>- Nutrition–MRI associations: unadjusted positive correlations were observed between cumulative 28-day nutrient intake and white matter volume, and between enteral nutrient intake and white matter ADC, but none remained significant after covariate adjustment<br><br>- Adjusted MRI findings: no significant associations were found between nutrient intake and white matter, total brain, cortical gray matter, ADC, or brain morphology outcomes
**Key neurodevelopmental outcomes**	- Follow-up: 59/72 (82%) had 2-year corrected-age follow-up; 49/72 (68%) had BSID-III scores, and 10/72 (14%) were assessed through child habilitation services<br><br>- BSID-III scores: cognitive index 103 ± 14 overall, motor index 101 ± 12, and language index 101 ± 17

	- Neurodevelopmental impairment: 11/72 (19%) overall; 4/approximately 11% in VPT infants and 7/29% in EPT infants<br><br>- Nutrition and neurodevelopment: unadjusted correlations were observed between BSID-III language scores and mean daily fluid, cumulative caloric, and carbohydrate intake, but these associations did not persist after covariate adjustment<br><br>- Overall result: early postnatal nutrient intake was not consistently associated with neurodevelopmental outcomes at 2 years
**Country**	Sweden

19. Nicole Bando

**First author**	Nicole Bando
**Year of publication**	
**Nutritional interventions**	- Intervention: In-hospital feeding with mother's milk provided first, supplemented as necessary with pasteurized donor milk or preterm formula under the randomized protocol<br><br>- Timing and duration: Parenteral nutrition began within 24 postnatal hours; enteral nutrition commenced after birth as directed by the medical team<br><br>- Supplementation duration: continued until 90 study days or hospital discharge<br><br>- Number of sessions: Not mentioned; the paper describes continuous nutritional provision rather than discrete sessions<br><br>- Mother's own milk: provided first; exposure was quantified as the percentage of total enteral feeds during the first 28 postnatal days<br><br>- Donor milk: pasteurized donor milk was used as necessary for supplementation; a Beneprotein module was added to donor milk to provide an average protein concentration of 1.2 g/dL<br><br>- Formula: preterm formulas had caloric strengths of 20 or 24 kcal/oz<br><br>- Fortification: human milk feeds, including mother's milk or donor milk, were fortified with a powder bovine-based human milk fortifier beginning at 120 mL/kg/day<br><br>- Fortification adjustment: enteral fortification was further increased if weight gain fell below 15 g/kg/day<br><br>- Feed advancement: volumes increased by 10–25 mL/kg/day, with full feeds defined as 160 mL/kg/day

**Anthropometric features and growth trajectories**	- Head circumference: Not mentioned.<br><br>- Growth trajectories: Not mentioned; no longitudinal anthropometric trajectories for weight, length, or head circumference were reported.<br><br>- Growth-adjusted models interpretation: Not applicable; models were adjusted for sex and birth gestational age, not growth measures.<br><br>- Reported anthropometric features: mean birth weight [1004 (267) g] and mean birth gestational age [27.8 (1.8) weeks].
**MRI results**	- MRI sample and acquisition: T1-weighted structural MRI was obtained in 40 five-year-old children born preterm with very low birth weight, using 3D-MPRAGE at 0.8-mm isotropic resolution<br><br>- MRI measures: 48 cortical, subcortical, and global brain volumes were analyzed, with bilateral hemispheres summed<br><br>- Volumes associated with Processing Speed Index: Total intracranial volume; total cortical gray matter; mid and posterior cingulate gyri; cunei; superior, middle, and inferior occipital gyri; fusiform gyri; and postcentral gyri were positively associated with processing speed<br><br>- Macronutrient and energy associations: No significant associations with the processing-speed-related MRI regions, except carbohydrate intake was negatively associated with posterior cingulate volume: −0.18 cm^3 (95% CI, −0.34 to −0.02; P = 0.03)<br><br>- Mother's milk associations: Per 10% increase in mother's milk intake, total intracranial volume increased by 11.91 cm^3 (95% CI, 5.27 to 18.55; P = 0.0009) and total cortical gray matter by 6.80 cm^3 (95% CI, 2.26 to 11.34; P = 0.004)<br><br>- Regional mother's milk associations: Positive associations were observed for mid cingulate gyri (0.16 cm^3; 95% CI, 0.06 to 0.25; P = 0.002), posterior cingulate gyri (0.06 cm^3; 95% CI, 0.01 to 0.10; P = 0.01), superior occipital gyri (0.17 cm^3; 95% CI, 0.01 to 0.33; P = 0.04), and postcentral gyri (0.46 cm^3; 95% CI, 0.22 to 0.70; P = 0.0004) per 10% increase in mother's milk<br><br>- Overall MRI interpretation: Early mother's milk provision was positively associated with total and regional cortical gray matter volumes related to processing speed, whereas early macronutrient intakes were largely unrelated to these MRI volumetric outcomes
**Key neurodevelopmental outcomes**	- TEA: Not mentioned; the follow-up assessment was performed at age 5 years, not at term-equivalent age<br><br>- Neurodevelopment outcome: WPPSI-IV Processing Speed Index, assessing timed visual processing and motor speed; mean score 102.2 (SD 11.4)<br><br>- Follow-up: MRI and WPPSI-IV assessment at approximately 5 years; mean MRI age 5.7 (SD 0.2) years<br><br>- Key neurodevelopmental results:<br><br>- Brain regions related to processing speed included total intracranial volume, total cortical gray matter, cingulate, occipital, fusiform, cuneus, and postcentral

	gyri]<br><br>- A 10% increase in mother's milk intake was associated with larger total intracranial volume, total cortical gray matter, mid/posterior cingulate gyri, superior occipital gyri, and postcentral gyri volumes<br><br>- Macronutrient and energy intakes were generally not associated with these brain regions, except for a negative association between carbohydrate intake and posterior cingulate volume
**Country**	Canada

20. Ottolini KM (2024)

**First author**	Ottolini KM
**Year of publication**	2024
**Nutritional interventions**	- Intravenous lipid emulsion: Started within 24 hours of NICU admission at 1 g/kg/day, then increased by 1 g/kg/day to a target of 3 g/kg/day. The intervention was administered daily; the paper does not report a separate number of feeding "sessions."<br><br>- Soybean-oil-only arm: 100% soybean-oil ILE (Intralipid), used for infants born before January 2019.<br><br>- Multicomponent arm: 30% soybean oil, 30% medium-chain triglycerides, 25% olive oil, and 15% fish oil (SMOFlipid), used for infants born after January 2019.<br><br>- Enteral nutrition: Primarily maternal or donor human milk, with at least two weeks of enteral nutrition before term-equivalent age. Intake was recorded daily from NICU admission until term-equivalent age.<br><br>- Feeding advancement: Standardized advancement based on birth weight and feeding tolerance toward 160 mL/kg/day.<br><br>- Fortification: Human milk was fortified with liquid bovine-based fortifier to 24 kcal/oz once feeding volume reached 100 mL/kg/day.<br><br>- Formula: Not reported; the cohort is described as receiving a human milk diet.
**Anthropometric features and growth trajectories**	- Anthropometric measures: Weekly weight, length, and head circumference were collected; growth failure was defined as a decrease in weight z-score of ≥1 SD from birth to term-equivalent age<br><br>- Growth failure: 77% (47/61) in the soybean-oil-only group vs 39% (12/31) in the multicomponent-emulsion group; P = .001<br><br>- Growth trajectories: Not mentioned (the paper reports a binary weight-based growth-failure measure rather than longitudinal trajectories; no head-circumference trajectory results are provided).

	- Growth-adjusted models: Growth failure was not included because it was interdependent with parenteral-nutrition duration; only parenteral-nutrition duration was incorporated into the models. Models adjusted for birth gestational age, MRI postmenstrual age, and clinically significant covariates, not for a reported growth trajectory or head circumference.
**MRI results**	- Volumetrics: [Among all 92 infants, soybean-oil-only (SO) exposure was associated with smaller brainstem volume after full covariate adjustment (least-squares mean 5.2 vs 5.7 cm^3 for multicomponent lipid emulsion [MLE]; β = −0.5, 95% CI −0.8 to −0.1; P = .007). Cortical gray-matter volume was smaller before full adjustment (β = −8.2, 95% CI −14.9 to −1.5; P = .02), but only borderline after adjustment (β = −7.5, 95% CI −15.2 to 0.3; P = .06); total brain, white-matter, deep-gray-matter, amygdala-hippocampal, and cerebellar volumes were not significantly different after adjustment.]<br><br>- White-matter maturation/microstructure: DTI was available for 81 infants. Compared with MLE, SO was associated with higher mean diffusivity in the corpus-callosum genu and splenium and both sides of the posterior limb of the internal capsule, lower fractional anisotropy in the right posterior limb of the internal capsule, and higher fractional anisotropy in the right and left pons; these findings were adjusted for birth gestational age, MRI postmenstrual age, parenteral-nutrition days, antenatal corticosteroids, and patent ductus arteriosus.<br><br>- MRI acquisition and measures: Non-sedated term-equivalent 3T MRI included T1/T2 structural imaging, automated plus manually corrected regional volumetry, and DTI-based FA and MD measurements in the corpus callosum, posterior limb of the internal capsule, and pons.
**Key neurodevelopmental outcomes**	- Assessment: At term-equivalent age, NNNS data were available for 67 infants (43 SO; 24 MLE)<br><br>- Quality of movement: higher with MLE than SO (LSM 5.2 vs 4.7; β = −0.54, 95% CI −0.97 to −0.11; P = .02)<br><br>- State-related stress: lower with MLE than SO (25% vs 51%; β = 1.41, 95% CI 0.14 to 2.83; P = .04)<br><br>- Lethargy: mean score was higher with MLE (6.0 vs 4.7; β = −1.35, 95% CI −2.46 to −0.25; P = .02), but lethargy incidence did not differ significantly (21% vs 14%; P = .62)<br><br>- Attention: higher in SO among infants assessed (LSM 4.7 vs 3.9; β = 0.79, 95% CI 0.16 to 1.42; P = .02), but 22% were not assessed because they did not achieve an alert state<br><br>- Follow-up: No long-term neurodevelopmental results were available; ongoing follow-up was underway<br><br>- Interpretation: The findings are associations, not evidence of direct causality
**Country**	United States

21. Sullivan G (2022)

**First author**	Sullivan G
**Year of publication**	2022
**Nutritional interventions**	- Scope: Preterm infants were categorized as having low breast milk exposure (n=68) or high breast milk exposure (n=67), with term controls (n=77)<br><br>- Feeding at MRI:<br><br>- Low-exposure group: breast milk 1 (1.5%), formula milk 42 (61.8%), mixed 22 (32.4%), unknown 3 (4.4%)<br><br>- High-exposure group: breast milk 25 (37.3%), formula milk 22 (32.8%), mixed 13 (19.4%), unknown 7 (10.4%)<br><br>- Term controls: breast milk 46 (59.7%), formula milk 10 (13.0%), mixed 17 (22.1%), unknown 4 (5.2%)<br><br>- Exclusive exposure during the neonatal period:<br><br>- Low-exposure group: 44% exclusive breast milk days and 16% exclusive formula days<br><br>- High-exposure group: 97% exclusive breast milk days and 0% exclusive formula days<br><br>- Mother's own milk: Not mentioned<br><br>- Donor milk: Not mentioned<br><br>- Formula: Reported as formula milk, including exclusive formula exposure<br><br>- Fortification strategy: Not mentioned<br><br>- Timing of nutritional intervention: Not mentioned; feeding status is reported at MRI and as percentages of exclusive milk days<br><br>- Number of intervention sessions: Not mentioned
**Anthropometric features and growth trajectories**	Not mentioned (the provided paper excerpt reports birthweight and birthweight z-score, but no head circumference, growth trajectories, or growth-adjusted model interpretation).
**MRI results**	- Cortical gray matter volume: $\beta = -0.192$; 95% CI, −0.342 to −0.042; adjusted $p = 0.017$<br><br>- Cortical thickness: $\beta = -0.132$; 95% CI, −0.284 to 0.019; adjusted $p = 0.087$; not statistically significant<br><br>- MRI sample: 68 low-breast-milk-exposure preterm infants, 67 high-exposure preterm infants, and 77 term controls

**Key neurodevelopmental outcomes**	- TEA neurodevelopmental outcome: Not mentioned (the supplied material reports cortical MRI morphology at term-equivalent age, but no neurodevelopmental test or outcome measure).<br><br>- Key imaging result: Cortic β al gray matter volume was associated with breast milk exposure (β = −0.192; 95% CI, −0.342 to −0.042; adjusted p = 0.017)<br><br>-<br><br>Neurodevelopmental follow-up: Not mentioned (no post-TEA neurodevelopmental follow-up results are included).
**Country**	Not mentioned (the provided text does not report the country).

22. William Hellström (2026)

**First author**	William Hellström
**Year of publication**	2026
**Nutritional interventions**	- Nutritional intervention: Enteral triglyceride oil (Formulaid®) providing arachidonic acid 100 mg/kg/day and docosahexaenoic acid 50 mg/kg/day, in addition to standard nutrition<br><br>- Timing: Started within 3 days after birth and continued until term-equivalent age; the abstract specifies until 40 weeks PMA<br><br>- Administration frequency: Daily supplementation, with the dose adjusted weekly according to body weight<br><br>- Number of sessions: Not mentioned (the study reports daily administration rather than a discrete number of sessions).<br><br>- Control nutrition: Standard nutrition according to national guidelines; no placebo was administered<br><br>- Mother's own milk, donor milk, formula and fortification: Not mentioned in the supplied paper text; only aggregate energy, lipid, protein and carbohydrate intakes during postnatal days 2–28 are reported in the nutritional-support table.
**Anthropometric features and growth trajectories**	- Head circumference: Not mentioned (the supplied paper reports MRI-derived brain volumes but no head-circumference measurements).<br><br>- Birth anthropometrics: Mean gestational age was 25.5 vs 25.8 weeks and mean birth weight was 787 vs 830 g in the standard-of-care and AA+DHA groups, respectively; birth-weight SD scores were 0.05 vs 0.16.<br><br>- Growth trajectories: There were no between-group differences in body weight at 36 or 40 weeks PMA or in change in weight z-score between birth and 36 or 40 weeks PMA.<br><br>- Growth-adjusted models: Adjusting the white-matter model for birth-weight

	SD score left the intervention association essentially unchanged: β=6.4 cm³, 95% CI 0.4 to 12.4, p=0.038.
**MRI results**	-<br><br>White-matter volume: 147.6 (22.1) cm³ with AA+DHA versus 138.3 (18.0) cm³ with standard care; adjusted β = 6.8 cm³, 95% CI 0.7 to 12.9, P = 0.028<br><br>- Total brain volume: higher in univariable analysis (β = 16.4 cm³; 95% CI 0.8 to 32.1; P = 0.04), but not significant after multivariable adjustment<br><br>- Cortical grey matter, central structures and cerebellum: no significant adjusted intervention differences<br><br>- Robustness: the white-matter effect persisted after adjustment for severe IVH (β = 7.2 cm³; 95% CI 1.0 to 13.3; P = 0.022)<br><br>- MRI timing: similar between groups, approximately 41.4 versus 41.3 weeks PMA
**Key neurodevelopmental outcomes**	- MRI brain volumetry at TEA (117 infants: 59 standard of care, 58 AA+DHA): AA+DHA supplementation was associated with significantly larger adjusted white-matter volume: β = 6.8 cm³; 95% CI, 0.7 to 12.9; P = 0.028, corresponding to approximately 10 days of prolonged gestation<br><br>- White-matter robustness: The association persisted after adjustment for severe IVH: β = 7.2 cm³; 95% CI, 1.0 to 13.3; P = 0.022<br><br>- Total brain volume: Higher with intervention in univariable analysis, β = 16.4 cm³; 95% CI, 0.8 to 32.1; P = 0.04, but not significant after multivariable adjustment<br><br>- Later neurodevelopmental follow-up: Not mentioned (the provided report evaluates MRI volumetry at TEA and does not report a later neurodevelopmental assessment).
**Country**	Sweden

23. F Patches (2023)

**First author**	F Patches
**Year of publication**	2023
**Nutritional interventions**	- Intervention type: Retrospective documentation and analysis of neonatal nutrition; no separately assigned nutritional intervention was reported<br><br>- Mother's own milk: Breast milk was used, and infants were classified as predominantly breast-milk fed or as receiving both breast milk and formula; the paper does not identify whether the breast milk was exclusively the mother's own milk<br><br>- Donor milk: Not mentioned

	- Formula: Formula milk was included; infants were classified as predominantly formula-fed or as receiving both breast milk and formula<br><br>- Fortification: A fortifier was part of the documented diet, but the type, timing, dose, and fortification strategy were not reported<br><br>- Enteral feeding: Started in the neonatal period via gastric tube and increased by 20–30 mL/kg/day; residuals up to 25% of the meal volume were tolerated in the absence of abdominal signs<br><br>- Parenteral nutrition: Used to complement enteral feeding, targeting 40–60 kcal/kg/day during the first days and 110–135 kcal/kg/day thereafter<br><br>- Protein strategy: Increased from 1.5–2.0 to 4.0–4.5 g/kg/day for infants <1000 g, or to 3.5–4.0 g/kg/day for infants 1000–1800 g, with subsequent adjustment according to growth<br><br>- Fat strategy: Started at 1.0 g/kg/day after birth and increased by approximately 0.5 g/kg/day to a maximum of 2.0–3.0 g/kg/day<br><br>- Timing: Nutrition was evaluated during postnatal weeks 1–4, cumulatively through postnatal days 14 and 28, and through corrected gestational age of 35 weeks<br><br>- Number of sessions: Not a fixed intervention schedule; infants <1500 g received 12 meals/day, changing to 8 meals/day at ≥1800 g and 6 meals/day at 2000 g, then ad libitum feeding before discharge
**Anthropometric features and growth trajectories**	- Cohort: 67 preterm infants; gestational age 28.9 ± 2.6 weeks and birth weight 1143 ± 364 g<br><br>- Head circumference: 26.06 ± 2.78 cm at birth, 30.60 ± 1.79 cm at 35 weeks' corrected gestational age, and 33.52 ± 1.89 cm at discharge<br><br>- Growth trajectory: weekly head-circumference gain rose from 0.27 ± 0.33 cm in week 1 to 0.87 ± 0.42 cm in week 4; mean gain to 35 weeks was 0.73 ± 0.20 cm/week<br><br>- Percentile trajectory: median head-circumference percentile declined from 40 at birth to 20 at discharge, suggesting limited catch-up growth during hospitalization<br><br>- Growth-adjusted DGM interpretation: head circumference at 35 weeks' corrected gestation was an independent positive correlate of DGM volume, together with positive fat intake and negative third-week protein intake; the model explained approximately 50% of DGM-volume variance<br><br>- Growth-adjusted LV interpretation: the independent predictors were first-week carbohydrate intake and intraventricular hemorrhage; together they explained approximately 19% of LV-volume variance
**MRI results**	- MRI at term-equivalent age showed a mean DGM volume of 18,865.55 ± 2,980.31 mm³; median 18,980.50 mm³; range 10,901.70–35,029.26 mm³.<br><br>- Left lateral ventricular volume: 3,508.18 ± 1,958.03 mm³; right lateral ventricular volume: 2,957.15 ± 1,252.15 mm³; combined LV volume: 6,465.32

	± 2,898.08 mm³; range 2,697.64–16,860.80 mm³.<br><br>- DGM volume associations:<br><br>- Head circumference at corrected 35 weeks: positive, $P < 0.001$<br><br>- Mean daily fat intake in postnatal week 4: positive, $P = 0.004$<br><br>- Mean daily protein intake in postnatal week 3: negative, $P = 0.007$<br><br>- The model explained $R^2 = 0.4978$, approximately 50% of DGM-volume variance.<br><br>- LV volume associations:<br><br>- Intraventricular hemorrhage: independent association, $P = 0.003$<br><br>- Mean daily carbohydrate intake in postnatal week 1: negative association, $P = 0.038$<br><br>- The model explained $R^2 = 0.1919$, approximately 19% of LV-volume variance.<br><br>- The univariable nutrition correlations included fourth-week fat intake with DGM volume ($r = 0.40918$), third-week protein intake with DGM volume ($r = -0.30878$), and first-week carbohydrate intake with LV volume ($r = -0.31418$).
**Key neurodevelopmental outcomes**	- Scope: 67 preterm infants were assessed with MRI at term-equivalent age (TEA), using DGM and lateral ventricular volumes as neurodevelopment-related structural outcomes<br><br>- DGM volume: positively associated with head circumference at 35 weeks' corrected gestational age and mean daily fat intake in postnatal week 4, and negatively associated with mean daily protein intake in postnatal week 3<br><br>- DGM explained variance: the three predictors explained nearly 50% of the variance in DGM volume ($R^2 = 0.4978$)<br><br>- Lateral ventricular volume: independently associated with intraventricular hemorrhage and mean daily carbohydrate intake in postnatal week 1<br><br>- Lateral ventricular direction: lower first-week carbohydrate supply was associated with enlarged lateral ventricles<br><br>- Neurodevelopmental follow-up: No post-TEA clinical neurodevelopmental follow-up outcome was reported; the authors indicated that the relevance of early nutrition requires confirmation in randomized controlled studies
**Country**	Germany

24. Gianluigi Laccet (2024)

**First author**	Gianluigi Laccetta

**Year of publication**	2024
**Nutritional interventions**	- Enteral nutrition: Started as soon as possible after birth, within 48 hours in stable infants; started after clinical stabilization in unstable infants. Began at 10–20 mL/kg/day and increased by 20–30 mL/kg each day according to tolerance<br><br>- Mother's own milk: Preferred; fresh maternal milk was administered enterally when available, without fortification-<br><br>Donor milk: Not available during the study period<br><br>- Formula: Preterm formula was given when maternal milk was unavailable or insufficient<br><br>- Fortification: No fortification of maternal milk was used; no separate fortification strategy was reported<br><br>- Parenteral nutrition: Started from birth and continued until exclusive enteral feeding of 100–120 kcal/kg/day was achieved<br><br>- Parenteral macronutrient regimen: Amino acids started at 2.5 g on day 1 and increased to 3.2 g/kg/day; glucose started at 6 and increased to 13 g/kg/day; lipids started at 1 and increased to 3.5 g/kg/day<br><br>- Intervention timing assessed: Enteral and parenteral energy and macronutrient intakes were quantified over the first 7 days of life<br><br>- Number of sessions: Not mentioned; the protocol reports daily volume advancement rather than a number of feeds or sessions per day
**Anthropometric features and growth trajectories**	- Scope: 42 preterm neonates<br><br>- Gestational age at birth: 29.14 ± 2.31 weeks<br><br>- Birth weight: 1266.60 ± 425.83 g<br><br>- Head circumference: Not mentioned<br><br>- Growth trajectories: Not mentioned<br><br>- Growth-adjusted models interpretation: Not mentioned
**MRI results**	Not mentioned (the study reports CUS findings rather than MRI results)
**Key neurodevelopmental outcomes**	- Population: 42 preterm neonates<br><br>- TEA outcome: Quantitative parieto-occipital periventricular white-matter echogenicity (RECP); mean right RECP 0.83 ± 0.12 and left RECP 0.79 ± 0.12<br><br>- Neurodevelopment outcome: No standardized neurodevelopmental test outcome was reported; the study assessed brain development only up to TEA, when designated neurodevelopmental testing was considered unreliable<br><br>- Key finding: Higher parenteral energy and amino-acid intake in the first week was positively associated with bilateral RECP at TEA

	- Adjusted results: Parenteral energy intake remained significant for right RECP (β = 0.544; p = 0.002) and left RECP (β = 0.562; p = 0.001); parenteral amino-acid intake remained significant for right RECP (β = 0.515; p = 0.005) and left RECP (β = 0.531; p = 0.004)<br><br>- Follow-up: No long-term neurodevelopmental follow-up was reported; the authors identified it as a future research need
**Country**	Italy

25. Tanya Poppe (2025)

**First author**	Tanya Poppe
**Year of publication**	2025
**Nutritional interventions**	- Intervention: NICU nutritional-protocol change increasing protein, energy, fat, and mineral intakes and decreasing total fluid intake<br><br>- Timing: Implemented in January 2007; the new-protocol group comprised infants born January 2007 to October 2008<br><br>- Nutritional exposure period: Measured over postnatal days 1–7 and 1–14; growth velocity was assessed through postnatal day 28<br><br>- Protein strategy: Higher intake in the new-protocol group: 21 (2) vs 17 (2) g/kg−1 on days 1–7 and 45 (7) vs 41 (6) g/kg−1 on days 1–14<br><br>- Number of sessions: Not reported; this was a continuous NICU protocol exposure rather than discrete sessions<br><br>- Mother's own milk: Not distinguished or specified; breast-milk intake was measured, and overall intake was similar between groups<br><br>- Donor milk: Not mentioned<br><br>- Formula: Not mentioned<br><br>- Fortification strategy: Not mentioned
**Anthropometric features and growth trajectories**	- Head circumference:<br><br>- At birth: 25 (2) cm in the old-protocol group vs 24 (2) cm in the new-protocol group; z score 0 (1) vs 0 (1)<br><br>- At 36 weeks: 31 (1) cm vs 31 (1) cm; z score −1 (1) vs −1 (1)<br><br>- At 7 years: 52 (2) cm vs 52 (2) cm; z score −1 (1) vs −1 (2)<br><br>- Other anthropometric features at 7 years:<br><br>- Weight: 24 (6) vs 26 (8) kg; weight z score 0 (1) vs 0 (1)

	- Height: 123 (6) vs 125 (6) cm; height z score 0 (1) vs 0 (1)<br><br>- Interpretation: no anthropometric differences were observed between protocol groups at 7 years<br><br>- Growth trajectories: not reported as longitudinal trajectories; only growth velocity at postnatal day 28 was reported, 11 (4) g/kg/d in both groups<br><br>- Growth-adjusted models: not mentioned; brain-structure analyses were adjusted for sex, birth-weight z score, gestational age, socioeconomic status, and clinical risk factors, with brain-volume adjustment for applicable outcomes rather than adjustment for growth trajectories
**MRI results**	- Brain volume: The new-protocol group had a lower brain-volume-to-intracranial-volume percentage than the old-protocol group (adjusted mean difference −5.75 percentage points; 95% CI, −7.77 to −3.73; FDR-corrected P < .001), but absolute brain volume did not differ significantly<br><br>- Cortical thickness: The new-protocol group had thinner bilateral lateral-occipital cortices and thinner right lateral-parietal cortex; the left lateral-parietal difference did not survive multiple-comparisons correction<br><br>- White-matter diffusion: There were no consistent between-group differences in diffusivity metrics, although the new-protocol group showed lower mean/radial diffusivity in selected cingulum angular bundles and the left uncinate fasciculus, and lower axial diffusivity in selected tracts<br><br>- White-matter maturation: Across both groups, greater protein, fat, energy, and breast-milk intake was associated with more mature diffusion metrics across almost every tract studied—higher fractional anisotropy and lower mean, axial, and radial diffusivity<br><br>- Nutrition–volume and cortical associations: Lower protein intake was associated with smaller brain volume relative to intracranial volume and thinner cortex, while greater fat, energy, and breast-milk intake was generally associated with greater relative brain volume and cortical thickness<br><br>- Deep gray matter: The new-protocol group had a larger pallidum before correction, but this was not significant after false-discovery-rate correction; higher protein intake was associated with greater thalamic volume, while fat, energy, and breast-milk intake were associated with larger nucleus accumbens and putamen volumes
**Key neurodevelopmental outcomes**	- Follow-up: At 7 years, there were no differences in neurodevelopmental outcomes between the old and new nutritional protocol groups.<br><br>- Neurodevelopmental impairment: 40% (17/42) in the old protocol group vs 37% (21/57) in the new protocol group; P = .71.<br><br>- Cerebral palsy: 0 in the old protocol group vs 11% (6/57) in the new protocol group; P = .09.
**Country**	New Zealand

26. Sissel J Moltu

**First author**	Sissel J Moltu
**Year of publication**	
**Nutritional interventions**	- ARA:DHA group: Enteral arachidonic acid 100 mg/kg/day plus docosahexaenoic acid 50 mg/kg/day (Formulaid™)<br><br>- Control group: Medium-chain triglyceride oil (MCT-oil™)<br><br>- Timing: Started on the second day of life at half dose (0.2 mL/kg), increased to the target dose (0.4 mL/kg) on day 4, and continued until 36 weeks PMA<br><br>- Number of sessions: Not reported as discrete sessions; supplementation was provided continuously/daily from day 2 of life until 36 weeks PMA<br><br>- Common nutritional care: Both groups received the same standardized protocol including parenteral nutrition, human milk, human-milk fortification, and transition to exclusive enteral nutrition<br><br>- Mother's own milk: Median intake from birth to 36 weeks PMA was similar between groups; Table 2 reports 133 ml/kg in control and 138 ml/kg in the ARA:DHA group<br><br>- Donor milk: Not mentioned<br><br>- Formula: Not mentioned as a milk source or specified by product/type<br><br>- Fortification strategy: Human milk was fortified within the standardized nutritional protocol, but the fortifier, dose, timing, and composition were not reported in the provided text
**Anthropometric features and growth trajectories**	- Baseline: Mean birth weight was 833 g in controls and 879 g in the ARA:DHA group; birth-weight z-score −1.25 vs −1.22. Mean birth head circumference was 23.9 vs 24.4 cm; head-circumference z-score −0.68 vs −0.64. Mean birth length was 33.5 vs 33.7 cm; length z-score −1.66 vs −1.86.<br><br>- Weight trajectory: Both groups regained their birth weight centiles by TEA. The adjusted between-group difference in change in weight z-score was −0.03 (95% CI −0.37 to 0.30) in the Kidokoro subgroup and 0.03 (95% CI −0.34 to 0.41) in the DTI subgroup.<br><br>- Head circumference trajectory: Both groups regained their birth head-circumference centiles by TEA. Head circumference was numerically larger in the ARA:DHA group at MRI, but the adjusted change in head-circumference z-score was not statistically significant: 0.25 (95% CI −0.15 to 0.65) in the Kidokoro subgroup and 0.34 (95% CI −0.10 to 0.79) in the DTI subgroup.<br><br>- Linear growth trajectory: Length growth was significantly greater in the ARA:DHA group. The adjusted difference in change in length z-score was 0.85 (95% CI 0.24 to 1.45; P = 0.006) in the Kidokoro subgroup and 0.91 (95% CI 0.18 to 1.63) in the DTI subgroup.<br><br>- Measurements at MRI: In the Kidokoro subgroup, mean weight was [3609 vs

	3536 g], head circumference [35.8 vs 36.2 cm], and length [49.7 vs 50.6 cm] in control versus ARA:DHA infants, respectively].
**MRI results**	- MRI population: 92 infants underwent MRI at term-equivalent age; 90 had successful T1/T2 imaging and 74 had acceptable-quality DTI for TBSS-<br><br>Brain morphology: Almost 95% had no or only mild Kidokoro abnormalities; global scores were 3.69 (2.39) in controls versus 3.18 (1.89) with ARA:DHA, adjusted mean difference −0.53 (95% CI, −1.41 to 0.35; P = 0.24)<br><br>- Mean and axial diffusivity: No significant between-group differences in MD or AD-<br><br>Fractional anisotropy and radial diffusivity: ARA:DHA supplementation produced significantly higher FA and lower RD in several major white-matter tracts, including the corpus callosum, anterior and posterior limbs of the internal capsule, cerebral peduncles, inferior fronto-occipital fasciculus, uncinate fasciculus, and inferior longitudinal fasciculus<br><br>- Robustness: The higher-FA finding was unchanged after adjustment for early-onset septicemia and protein intake
**Key neurodevelopmental outcomes**	- At TEA, among 74 infants with evaluable DTI, ARA:DHA supplementation was associated with significantly higher fractional anisotropy and lower radial diffusivity in several major white-matter tracts, including the corpus callosum, internal capsule, cerebral peduncles, inferior fronto-occipital, uncinate and inferior longitudinal fasciculi; mean and axial diffusivity did not differ significantly.<br><br>- Global brain abnormality was not significantly different: adjusted Kidokoro-score difference −0.53 (95% CI −1.41 to 0.35; P = 0.24).<br><br>- No post-TEA neurodevelopmental or functional follow-up outcome was reported; the authors state that long-term functional effects require further study.<br><br>- The ARA:DHA group had greater linear growth to TEA in the DTI subgroup: adjusted difference in change in length z-score 0.91 (95% CI 0.18 to 1.63).
**Country**	Norway

27. Duerden EG (2021)

**First author**	Duerden EG
**Year of publication**	2021
**Nutritional interventions**	- Nutritional intervention: No distinct randomized or session-based nutritional intervention was described; the study prospectively recorded actual parenteral and enteral intake. A planned change in clinical care increased protein intake to better meet international recommendations, but the protocol and number of sessions were not reported planned_protein_change.

	- Timing: Nutritional intake was recorded daily for the first 28 days and summarized for the first 7 days and first 28 days.<br><br>- Number of sessions: Not mentioned (nutrition was recorded as daily intake rather than as discrete intervention sessions).<br><br>- Mother's own milk: Breast milk was included in the intake assessment.<br><br>- Donor milk: Not mentioned.<br><br>- Formula: Formula was included in the intake assessment, with composition based on commercial notifications.<br><br>- Fortification strategy: Not mentioned.
**Anthropometric features and growth trajectories**	Not mentioned (head circumference, growth trajectories, and growth-adjusted model interpretations are not reported)
**MRI results**	- MRI results were based on resting-state functional connectivity in 56 very preterm-born children at 7 years; no volumetric or morphometric MRI outcomes were reported. Robust anterior default-mode and thalamic networks were identified, with connectivity strength ranging from 0–1 resting_state_networks,mri_sample<br><br>- Thalamic–aDMN connectivity:<br><br>- First-week protein intake: β = 0.17; 95% CI 0.11–0.23; p < 0.001<br><br>- First-week fat intake: β = −0.06; 95% CI −0.09 to −0.02; p = 0.001<br><br>- First-week carbohydrate intake: β = −0.03; 95% CI −0.04 to −0.01; p = 0.003<br><br>- First-28-day protein intake: β = 0.22; 95% CI 0.06–0.37; p = 0.006<br><br>- Associations with 7-year outcomes:<br><br>- Processing speed index: β = 26.9; 95% CI 4.21–49.49; p = 0.02<br><br>- Beery visual perception: β = 9.03; 95% CI 2.27–15.79; p = 0.009<br><br>- No significant associations with working memory, perceptual reasoning, verbal comprehension, full-scale IQ, Beery visuomotor integration, or motor scores connectivity_cognitive_results,connectivity_visual_results
**Key neurodevelopmental outcomes**	- At 7 years, most children scored within the typical range on the WISC-V; median scores were FSIQ 95, VCI 98, WMI 94, PSI 97, PRI 90, with Beery medians of 94 for visuomotor integration, 101 for visual perception, and 92 for motor ability. Most children were in the typical range, and outcome medians are reported in Table 1<br><br>- Processing speed: greater thalamocortical connectivity was associated with higher WISC processing speed scores (β = 26.9; 95% CI 4.21–49.49; p = 0.02). processing_speed_association<br><br>- Visual perception: greater thalamocortical connectivity was associated with higher Beery visual perceptual scores (β = 9.03; 95% CI 2.27–15.79; p =

	0.009). visual_perception_association<br><br>- Other outcomes: no significant associations were reported for working memory, perceptual reasoning, verbal comprehension, full-scale IQ, Beery visuomotor integration, or motor scores.
**Country**	New Zealand

28. Julie Sato (2021)

**First author**	Julie Sato
**Year of publication**	2021
**Nutritional interventions**	- Intervention: Donor milk or preterm formula was provided as a supplement to mother's own milk; a third group received mother's own milk exclusively.<br><br>- Mother's own milk: Exclusive mother's breastmilk was one feeding group, defined as no donor milk or preterm formula supplementation.<br><br>- Donor milk: Donor milk supplementation comprised at least 20% of total enteral feeds in the donor-milk group.<br><br>- Formula: Preterm formula supplementation comprised at least 20% of total enteral feeds in the preterm-formula group.<br><br>- Timing: The feeding intervention began during the initial hospitalization and lasted 90 days or until hospital discharge, whichever came first.<br><br>- Feeding frequency/sessions: Not mentioned; nutrition was recorded as daily parenteral and enteral volumes and compositions rather than as a specified number of sessions.<br><br>- Fortification strategy: Nutrient fortification and full enteral feeding were largely established during postnatal days 9–29, but the fortifier type and dosing strategy were not reported in the supplied text.<br><br>- Macronutrient assessment: Daily nutrition was summarized for postnatal days 1–8 and 9–29; analyses focused on days 9–29 because fewer than 10% achieved recommendations during days 1–8.<br><br>- Comparative outcome: No significant white-matter differences were found between donor-milk and preterm-formula supplementation, or between either supplementation group and exclusive mother's breastmilk.
**Anthropometric features and growth trajectories**	- Anthropometric features: Mean age at scan was 5.8 years, mean gestational age was 28.1 weeks, and mean birth weight was 1028.6 g<br><br>- Head circumference: Not measured or reported for this cohort; it is discussed only as a prior-study indicator correlated with brain growth and later cognitive and motor outcomes<br><br>- Growth trajectories: Not mentioned (the paper does not present longitudinal anthropometric growth trajectories).

	- Growth-adjusted models interpretation: Not applicable (the reported models adjusted for sex and birth gestational age or birth weight in imaging analyses, not for anthropometric growth trajectories).
**MRI results**	- MRI sample and measures: 41 children with complete multi-shell scans; diffusion MRI measures included fractional anisotropy (FA), radial diffusivity (RD), orientation dispersion index (ODI) and neurite density index (NDI)<br><br>- Protein intake: Meeting enteral protein recommendations during postnatal days 9–29 was associated with increased FA and lower RD across widespread tracts, including the corpus callosum, corona radiata, anterior limb of the internal capsule and posterior thalamic radiation<br><br>- Energy intake: Meeting enteral energy recommendations was associated with increased FA and reduced RD<br><br>- Lipid intake: Higher lipid intake was associated with higher FA and lower RD, with less widespread effects concentrated in posterior tracts including the posterior thalamic radiation and corpus callosum<br><br>- Breastmilk, breastfeeding duration and carbohydrate intake: No significant associations with DTI or NODDI metrics<br><br>- Donor milk versus preterm formula or exclusive maternal breastmilk: No significant differences in DTI or NODDI metrics<br><br>- MRI–cognition associations: Higher FA and NDI, and lower RD, were associated with better processing speed in overlapping regions including the corpus callosum and posterior thalamic radiation; lower ODI was associated with processing speed in a corpus-callosum cluster<br><br>- Other cognitive outcomes: No significant associations with DTI or NODDI metrics
**Key neurodevelopmental outcomes**	- At approximately 5.8 years, mean WPPSI-IV cognitive scores were in the average range; full-scale IQ was 102.56 and processing speed was 102.34, while 7/41 children (17.1%) had full-scale IQ scores below 90.<br><br>- Children meeting enteral protein, lipid, and energy recommendations during postnatal days 9–29 had improved white matter microstructure, characterized by increased fractional anisotropy and reduced radial diffusivity; protein intake showed the most widespread associations.<br><br>- Better processing speed was associated with higher fractional anisotropy and neurite density index, and lower radial diffusivity and orientation dispersion index.<br><br>- No significant associations were found with other cognitive outcomes, breastmilk intake, breastfeeding duration, or donor-milk versus preterm-formula supplementation.
**Country**	Canada

29. Samuel Caruggi (2026)

**First author**	Samuele Caruggi
**Year of publication**	2026
**Nutritional interventions**	- Intervention: parenteral nutrition containing either soybean lipid emulsion (SLE; Intralipid® 20%) or multicomponent lipid emulsion (MLE; SMOFlipid® 20%).<br><br>- Timing: Intralipid® was used for infants born from 2017 through December 2018; from January 2019 through December 2021, all parenteral nutrition contained SMOFlipid®.<br><br>- Duration: median parenteral-nutrition exposure was 17 days (IQR 10) with Intralipid® and 21 days (IQR 14) with SMOFlipid®.<br><br>- Number of sessions/doses: Not mentioned.<br><br>- Mother's own milk, donor milk, formula, and fortification strategy: Not mentioned; detailed nutritional intake was unavailable.
**Anthropometric features and growth trajectories**	- Baseline anthropometrics:<br><br>- Gestational age: 29.5 weeks in Intralipid vs 29.8 weeks in SMOFlipid; adjusted p = 0.65<br><br>- Birth weight: 1,160 g vs 1,190 g; adjusted p = 0.75<br><br>- Anthropometrics at MRI:<br><br>- Weight: 2,970 g vs 2,870 g; adjusted p = 0.50<br><br>- Weight Z-score: −1.13 vs −1.10; adjusted p = 0.50<br><br>- Head circumference: Not mentioned<br><br>- Growth trajectories: Not mentioned; only baseline and MRI-time anthropometric measures were reported<br><br>- Growth-adjusted models interpretation: Not applicable; no growth-adjusted model was reported
**MRI results**	- MRI timing: Brain MRI was performed at term-equivalent age, between 39 and 41 weeks of corrected age<br><br>- Total maturation score: Intralipid 13 (IQR 3) vs SMOFlipid 12 (IQR 2); adjusted p = 0.09<br><br>- PLIC visibility, T2-weighted MRI: Intralipid median 2 vs SMOFlipid median 1; adjusted p = 0.07<br><br>- PLIC visibility, T1-weighted MRI: median 2 in both groups; adjusted p = 0.50<br><br>- MRI inter-rater reliability: Cohen's κ = 0.87, indicating almost-perfect agreement

	- TMS composition: myelination, cortical convolutions, germinal matrix, and bands of migrating glial cells; total range 4–21 points
**Key neurodevelopmental outcomes**	- TEA brain maturation: TMS was numerically higher with Intralipid than SMOFlipid (median 13 vs. 12), but not statistically significant after adjustment (adjusted p = 0.09); PLIC T2 visibility also favored Intralipid (median 2 vs. 1) but was not significant after adjustment (adjusted p = 0.07)<br><br>- Neurodevelopment at 24 months corrected age: Griffiths III total scores were identical (median 92 vs. 92; adjusted p = 0.87), with no significant differences in locomotor, personal-social, hearing and speech, or performance domains<br><br>- Eye and hand coordination: higher with SMOFlipid than Intralipid (median 93 vs. 85; adjusted p = 0.02)<br><br>- Overall interpretation: the lipid emulsion type did not significantly influence overall neurodevelopmental outcomes
**Country**	Italy

30. Zhenzhen Qing (2025)

**First author**	Zhenzhen Qing
**Year of publication**	2025
**Nutritional interventions**	Not mentioned (no assigned nutritional intervention or number of intervention sessions was reported; the study retrospectively analyzed first-week intake and feeding records)
**Anthropometric features and growth trajectories**	- Study population: 192 preterm infants—74 with IUGR and 118 without IUGR<br><br>- Anthropometric features:<br><br>- At birth, IUGR infants had lower birth weight, head circumference, length, and corresponding z-scores than non-IUGR infants; for head circumference, values were 27.5 vs 29.0 cm and z-scores −1.20 vs −0.13. At discharge, head circumference was 31.6 vs 32.4 cm and head-circumference z-score −1.53 vs −0.36; length was 44.7 vs 46.4 cm and length z-score −1.81 vs −0.46.<br><br>- Growth trajectories: IUGR infants remained smaller at discharge, indicating persistent postnatal growth restriction and abnormal growth trajectories after birth-<br><br>Head circumference: Discharge head-circumference z-score was positively associated with BFD/CI and FH-R/CI at TEA<br><br>- Other growth associations: Discharge length z-score was positively associated with BFD/CI, FH-L/CI, and TCD/CI; discharge length was positively associated with FH-R/CI<br><br>- Growth-adjusted model interpretation: The models identify postnatal growth indicators—especially discharge length and head-circumference z-scores—as

	independent correlates of regional brain measurements, rather than treating IUGR status alone as the complete explanation<br><br>- Head-size adjustment: All MRI brain metrics were adjusted using the cephalic index calculated from biparietal and occipitofrontal diameters<br><br>- Brain-development pattern after adjustment: IUGR infants had smaller adjusted BFD, frontal-lobe heights, transverse cerebellar diameter, and genu/body of the corpus callosum, while the splenium did not differ significantly
**MRI results**	- MRI modality and timing: T1-weighted cerebral MRI at term-equivalent age, using linear measurements of the bifrontal diameter, bilateral frontal-lobe heights, transverse cerebellar diameter, and genu, body, and splenium of the corpus callosum; measurements were adjusted for cephalic index<br><br>- Group comparison: IUGR infants had smaller BFD/CI (7.40 vs 7.87 mm; P=0.004), FH-L/CI (5.33 vs 5.64 mm; P=0.04), FH-R/CI (5.36 vs 5.72 mm; P=0.04), TCD/CI (median 5.59 vs 5.83 mm; P=0.049), genu of CC/CI (median 0.0240 vs 0.0266 mm; P=0.02), and body of CC/CI (median 0.0161 vs 0.0188 mm; P=0.02) than non-IUGR infants; splenium of CC/CI did not differ significantly (P=0.65)<br><br>- Measurement reliability: Interobserver ICCs were excellent, ranging from 0.862 to 0.964<br><br>- Nutrition associations: In non-IUGR infants, first-week protein intake was positively correlated with BFD/CI (r=0.269; P=0.04), FH-L/CI (r=0.302; P=0.02), and FH-R/CI (r=0.286; P=0.03); no significant nutrition–MRI associations were observed in IUGR infants<br><br>- Independent clinical associations: MRI measurements were associated with postnatal growth, breastfeeding duration, gestational age, birth weight, gestational diabetes, respiratory distress syndrome, and intraventricular hemorrhage, depending on the brain region
**Key neurodevelopmental outcomes**	- TEA neurodevelopment outcome: IUGR infants had smaller normalized BFD, left and right frontal lobe heights, transverse cerebellar diameter, and genu and body of the corpus callosum; splenium measurements did not differ significantly from non-IUGR infants<br><br>- Early nutrition: In non-IUGR infants, first-week protein intake was positively associated with BFD/CI, FH-L/CI, and FH-R/CI; no significant nutrition–brain measurement associations were found in IUGR infants<br><br>- Other associated factors: Discharge length and head-circumference measures, breastfeeding duration, gestational age, birth weight, GDM, RDS, and IVH were independently associated with different cerebral measurements at TEA<br><br>- Follow-up: No post-discharge neurodevelopmental follow-up outcome was reported; the study evaluated MRI brain measurements at TEA in 192 infants
**Country**	China

31. L. M. Hortensius (2021)

**First author**	L. M. Hortensius
**Year of publication**	2021
**Nutritional interventions**	- Intervention: Cohort B received a new regimen with more rapidly increased, higher protein intake than cohort A; protein, lipid, and caloric intake were assessed during the first 28 postnatal days<br><br>- Timing and administration:<br><br>- Cohort A: parenteral nutrition began on postnatal day 2, with protein and lipids increased gradually until day 5<br><br>- Cohort B: parenteral nutrition began as soon as possible after admission, starting at 1 g/kg protein and lipid intake and increasing to maximum protein 3.5 g/kg and lipids 4 g/kg on postnatal day 3<br><br>- Both cohorts: minimal enteral feeding began shortly after birth, at 24–48 hours, and was increased daily if tolerated<br><br>- Number of sessions: Not mentioned; the regimen is described by postnatal timing and daily intake escalation rather than discrete intervention sessions<br><br>- Mother's own milk: Not distinguishable; the authors state that they had no information on breast-milk intake<br><br>- Donor milk: Not mentioned<br><br>- Formula: Not distinguishable; the authors state that they had no information on formula intake<br><br>- Fortification strategy: Not mentioned; breast-milk composition was assumed to be 68 kcal, 1.0 g protein, and 4.0 g lipid per 100 mL for calculation purposes<br><br>- Macronutrient contrast: Cohort B had higher protein and protein/energy ratio, whereas lipid intake was similar between cohorts
**Anthropometric features and growth trajectories**	- Head circumference: Not mentioned; the reported baseline anthropometric variables include gestational age, birth weight, birth-weight Z-score, and SGA status, but not head circumference-<br><br>Growth trajectories: Not reported; postnatal growth is mentioned only as background in relation to brain development, with no serial growth-trajectory analysis<br><br>- Growth-adjusted models interpretation: The multivariable models adjust for gestational age and birth-weight Z-score, along with clinical and maternal covariates; they do not model head circumference or growth trajectories
**MRI results**	- MRI modality and outcome: DTI at term-equivalent age assessed white matter integrity using fractional anisotropy; 123 infants were included in the DTI analysis<br><br>- Cohort comparison: Cohort B had significantly higher FA than cohort A in the body of the corpus callosum, superior and posterior corona radiata, superior

	longitudinal fasciculus, retrolenticular internal capsule, left sagittal stratum and posterior thalamic radiation, and right posterior limb of the internal capsule and superior cerebral peduncle<br><br>- Protein and FA: Higher total protein intake during the first 28 postnatal days was significantly positively associated with FA in similar white matter tracts<br><br>- Enteral protein: Cumulative enteral protein intake was positively associated with FA in the left posterior thalamic radiation; parenteral protein intake was not significantly associated with FA<br><br>- Lipids and calories: Total, enteral, and parenteral lipid and caloric intake were not significantly associated with FA<br><br>- Volumetrics: The current study did not report a new volumetric MRI effect at TEA; an earlier study found globally increased brain volumes at 30 weeks PMA, but not at TEA, after the higher-protein regimen
**Key neurodevelopmental outcomes**	- TEA white matter integrity: Cohort B and higher early protein intake were associated with higher fractional anisotropy in several white matter tracts, including the corpus callosum, corona radiata, superior longitudinal fasciculus, internal capsule, sagittal stratum, posterior thalamic radiation, and superior cerebral peduncle.<br><br>- Motor outcome at 2 years CA: Cohort B had lower total motor scores than cohort A (103 (12) vs. 109 (12); P = 0.005), mainly due to lower fine motor scores (11 (2.6) vs. 13 (2.2); P = 0.002), although scores remained within the normal range.<br><br>- Adjusted cohort effect at 2 years CA: The nutritional cohort was associated with lower motor scores (B = −5.2; 95% CI, −8.9 to −1.5), but not with cognition.<br><br>- Protein–motor association at 2 years CA: Higher daily protein intake was associated with lower motor scores (B = −6.7; 95% CI, −10.8 to −2.7), with no significant association with cognition.<br><br>- Follow-up at 5.9 years: Cognitive and motor outcomes were similar between cohorts and were within the normal range.<br><br>- Overall: Higher early protein intake was associated with improved white matter integrity but did not improve long-term neurodevelopmental outcomes.
**Country**	Netherlands

32. Valdes C. (2024)

**First author**	Valdes C.
**Year of publication**	2024
**Nutritional interventions**	- Intervention design: A unit nutrition protocol was described; the study did not report a discrete trial intervention or a fixed number of intervention sessions

	- Parenteral nutrition: Started on day 0; anticipated duration 1–2 weeks. Lipids started at 3 g/kg/day, protein was advanced to 3.5 g/kg/day, and glucose infusion rate was titrated from 4–5 to 10–12 g/kg/min<br><br>- Enteral feeding: Small-volume enteral/gavage feeds started on day 0–1 if medically stable, with advancement of 10–30 mL/kg/day as tolerated<br><br>- Colostrum: For infants <1250 g, 0.2 mL buccal priming every 6 hours for 7 days<br><br>- Mother's own milk: Preferred unless contraindicated<br><br>- Donor milk: Alternative to mother's milk; indicated for infants <30 weeks' gestation<br><br>- Formula: Used when breast milk was unavailable: special care formula 24 kcal/oz at 30–34 weeks, Neosure 22 kcal/oz at 34–36 weeks, and Similac 19 kcal/oz after 37 weeks<br><br>- Fortification: Mother's milk or donor milk fortified with human milk fortifier to 22 kcal/oz at 60 mL/kg/day and to 24 kcal/oz at 100 mL/kg/day<br><br>- Number of sessions: Not mentioned for nutritional interventions; the protocol reports continuous/daily feeding management rather than session counts. Scheduled monitoring occurred weekly from week 2, with daily weight and weekly length/head-circumference measurements<br><br>- Feed monitoring: After transition from parenteral to enteral feeding, the amount and type of milk or formula were routinely recorded as percentages by the dietitian
**Anthropometric features and growth trajectories**	- Head circumference: Measured weekly, alongside weekly length measurements; no head-circumference growth trajectory or head-circumference-adjusted brain-volume result was reported<br><br>- Growth trajectories: Weight and length were evaluated using maximum changes in z-scores; an uncomplicated postnatal weight trajectory was described as 0.8 SD below birth at day 21<br><br>- Malnutrition classification: A decline of 0.8–1.2 SD was mild, >1.2–2 SD was moderate, and >2 SD was severe malnutrition<br><br>- Cohort growth: Mean maximum declines were −1.33 for weight z-score and −1.51 for length z-score, both indicating moderate malnutrition<br><br>- Growth-adjusted models: Linear regression examined marginal associations and associations conditional on potential confounders, using additive and interaction models with sequential removal of nonsignificant higher-order terms<br><br>- Interpretation: Maximum weight- and length-z-score changes were positively associated with brain tissue volumes at term equivalent age, and length z-score was positively associated with brain volume across gestational ages
**MRI results**	- MRI volumetry: Manual MRI segmentation measured total supratentorial brain tissue volume, including cerebral cortex, white and grey matter, basal ganglia, and thalami; mean volume was 235,059 ± 37,045 mm^3 in 198 infants

	- Gestational-age volumetrics: Mean total brain volumes ranged from 205,882 mm³ at 27 weeks' gestation to 244,605 mm³ at 25 weeks' gestation-<br><br>Nutritional/maturation associations: Greater maximum weight and length z-scores were significantly positively associated with total brain tissue volume at term-equivalent age; length z-score was also positively associated with brain volume across gestational ages ($p < 0.05$)<br><br>- Clinical associations: Brain tissue volumes were significantly lower with bronchopulmonary dysplasia and clinical sepsis, while no association was found with several other maternal or infant factors, including antenatal or postnatal steroids, IUGR, mechanical ventilation, hsPDA, gastrointestinal injury, or sedation<br><br>- Birth-size subgroups: Mean brain volumes were 227,595.5 mm³ in SGA infants, 236,135.1 mm³ in AGA infants, and 231,100.0 mm³ in LGA infants
**Key neurodevelopmental outcomes**	- TEA brain outcome: Maximum changes in weight and length z-scores were significantly positively associated with total brain tissue volumes at TEA ($p < 0.05$); mean brain volume was 235,059 ± 37,045 mm³ in 198 infants<br><br>- Neurodevelopmental outcome: No direct neurodevelopmental assessment or follow-up outcome was reported; neonatal MRI brain volume was presented as a surrogate measure of early brain development<br><br>- Follow-up: Prospective sequential MRI and subsequent neurodevelopmental outcomes were proposed for future studies rather than reported in this cohort
**Country**	United States

33. Giovanni Boscarino

**First author**	Giovanni Boscarino
**Year of publication**	
**Nutritional interventions**	- Enteral nutrition: Started as soon as possible after birth; minimal enteral feeding began at 10–20 ml/kg/day and was increased by 20–30 ml/kg/day when tolerated. The study table reports initiation at 1 ± 1 days of life and full enteral feeding at 14 ± 13 days of life.<br><br>- Mother's own milk: Human milk from the infant's own mother was administered as soon as possible after birth.<br><br>- Donor milk: Not mentioned.<br><br>- Preterm formula: Used when mother's milk was unavailable or insufficient; the formula was Pre-Nidina Nestlé, and its macronutrient content was calculated from the manufacturer's label.<br><br>- Fortification: Not mentioned; no human-milk fortification strategy was reported.<br><br>- Parenteral nutrition: Started early after birth via central vascular access and

	continued until full enteral feeding at 120 kcal/kg/day; the table reports a PN duration of 14 ± 13 days.<br><br>- Timing of assessment: Daily EN and PN intakes were collected during the first week of life, and cerebral growth was evaluated over the first 28 days of life.<br><br>- Number of sessions: Not mentioned; the protocol describes daily nutritional intake rather than a fixed number of sessions.
**Anthropometric features and growth trajectories**	- Anthropometric features: Gestational age was 29 ± 2 weeks and birth weight was 1288 ± 362 g; head circumference was not reported.<br><br>- Growth trajectories: Cerebral growth was assessed from 3–7 days of life (T0) to 28 days (T1), calculated as (T1−T0)/T0.<br><br>- Growth-adjusted model interpretation: Multivariable analyses adjusted for birth weight, gender, cord-blood pH, morbidity, and EN or PN energy intake; birth weight remained positively associated with selected cerebral-growth measures.<br><br>- Nutrition-related trajectories: PN energy intake was negatively correlated with right caudate-head growth (r = −0.243), whereas total energy intake was positively correlated with transverse cerebellar-diameter growth (r = 0.254).
**MRI results**	Not mentioned (the study used cUS rather than MRI for cerebral measurements)
**Key neurodevelopmental outcomes**	- TEA: Not mentioned; the study did not report a TEA outcome.<br><br>- Neurodevelopment outcome: Not assessed in this study; the authors could not establish whether cerebral measurements at 28 days had later neurodevelopmental consequences<br><br>- Follow-up: cUS assessments were performed at 3–7 days and 28 days of life<br><br>- Prior related finding: A previous study in a similar population reported that high PN energy intake was associated with reduced neurodevelopment
**Country**	Italy

To visualize Table S1, click on the following icon (Microsoft Excel's file xlsx):

Foglio di lavoro di Microsoft Excel
